# Visual and genetic stock identification of a test fishery to forecast Columbia River spring Chinook salmon stocks 2 weeks into the future

**DOI:** 10.1111/eva.13667

**Published:** 2024-03-08

**Authors:** Jon E. Hess, Bethany M. Deacy, Michelle W. Rub, Donald M. Van Doornik, John M. Whiteaker, Jeffrey K. Fryer, Shawn R. Narum

**Affiliations:** ^1^ Columbia River Inter‐Tribal Fish Commission Portland Oregon USA; ^2^ Washington Department of Fish and Wildlife Ridgefield Washington USA; ^3^ Fish Ecology Division, Northwest Fisheries Science Center National Marine Fisheries Service, National Oceanic and Atmospheric Administration Seattle Washington USA; ^4^ Conservation Biology Division, Northwest Fisheries Science Center National Marine Fisheries Service, National Oceanic and Atmospheric Administration Port Orchard Washington USA; ^5^ Columbia River Inter‐Tribal Fish Commission Hagerman Idaho USA

**Keywords:** fisheries management, migratory species, parentage, population genetics – empirical

## Abstract

Modern fisheries management strives to balance opposing goals of protection for weak stocks and opportunity for harvesting healthy stocks. Test fisheries can aid management of anadromous fishes if they can forecast the strength and timing of an annual run with adequate time to allow fisheries planning. Integration of genetic stock identification (GSI) can further maximize utility of test fisheries by resolving run forecasts into weak‐ and healthy‐stock subcomponents. Using 5 years (2017–2022) of test fishery data, our study evaluated accuracy, resolution, and lead time of predictions for stock‐specific run timing and abundance of Columbia River spring Chinook salmon (*Oncorhynchus tshawytscha*). We determined if this test fishery (1) could use visual stock identification (VSI) to forecast at the coarse stock resolution (i.e., classification of “lower” vs. “upriver” stocks) upon which current management is based and (2) could be enhanced with GSI to forecast at higher stock resolution. VSI accurately identified coarse stocks (83.3% GSI concordance), and estimated a proxy for abundance (catch per unit effort, CPUE) of the upriver stock in the test fishery that was correlated (*R* ^2^ = 0.90) with spring Chinook salmon abundance at Bonneville dam (Rkm 235). Salmon travel rates (~8.6 Rkm/day) provided predictions with 2‐week lead time prior to dam passage. Importantly, GSI resolved this predictive ability as finely as the hatchery broodstock level. Lower river stock CPUE in the test fishery was correlated with abundance at Willamette Falls (Rkm 196, *R* ^2^ = 0.62), but could not be as finely resolved as achieved for upriver stocks. We described steps to combine VSI and GSI to provide timely in‐season information and with prediction accuracy of ~12.4 mean absolute percentage error and high stock resolution to help plan Columbia River mainstem fisheries.

## 1 INTRODUCTION

Test fisheries can provide data on strength and timing and estimates of stock composition of an annual run of anadromous fishes in advance of scheduled fisheries to inform management. Examples of test fisheries include those for Sockeye salmon (*Oncorhynchus nerka*) in the Port Moller Test Fishery conducted in Bristol Bay, Alaska (since 1967, Tiernan et al., 2021) and the Skeena Tyee Test Fishery that occurs on the Skeena River (since 1955, Beacham et al., 2014; Labelle, 2009); Chum salmon (*Oncorhynchus kisutch*) test fishing in Puget Sound near Kingston, WA (operated by Northwest Indian Fisheries Commission and Washington Department of Fish and Wildlife since 1982, Matthews, 2012, https://nwifc.org); and Smelt (*Osmerus eperlanus*) test fishing in Lake Tuusulanjärvi in Finland (since 1998, Rask et al., 2020). The greatest benefits of test fisheries are realized when predictions comprise the highest levels of resolution and accuracy of stock‐specific data and with adequate timing to allow for fisheries planning (Flynn & Hilborn, 2004). The period of time deemed as “adequate timing” may depend on each management application, but in the case of the Port Moller Test Fishery, delivery of estimates for strength, timing, and age, size and stock composition of the run just 6–9 days in advance of the arrival of fish at inshore fishing districts has been adequate lead time to manage fisheries (Tiernan et al., 2021).

The Spring Chinook Salmon Test Fishery (SCTF) on the lower Columbia River mainstem was initiated in 2004 and has served to measure a number of coarse‐level stock composition characteristics to inform fisheries management of non‐treaty fisheries (i.e., Oregon and Washington state‐managed fisheries) conducted within the mainstem from the river mouth at the Pacific Ocean to Bonneville dam (first major dam on the mainstem Columbia River, 235 Rkm). These coarse‐level stock characteristics include estimates of the relative proportions of two species (steelhead, *Oncorhynchus mykiss*; and Chinook salmon, *Oncorhynchus tshawytscha*), relative proportions of two stocks of Chinook salmon (lower river vs. upriver), and relative proportions of adipose‐clipped (hatchery origin) and adipose‐intact (putative natural origin) Chinook salmon. These characteristics are all tied to management objectives for stocks that are regulated in mainstem fisheries during the spring management period (January 1–June 15), and include avoidance of steelhead (comprising winter run components that are ESA listed; lower Columbia and upper Willamette DPS), meeting a *U.S. v OR* Management Agreement provision for the upriver stock to not exceed the total allowable catch available for treaty fisheries (i.e., fisheries managed by Columbia River Inter‐Tribal Fish Commission's four member tribes; the Confederated Tribes and Bands of the Yakama Nation, the Confederated Tribes of the Umatilla Indian Reservation, the Confederated Tribes of Warm Springs Reservation of Oregon, and the Nez Perce Tribe), and minimizing impacts to natural origin stocks of either upriver (comprising ESA‐listed Snake River spring/summer and upper Columbia spring stock subcomponents as well as stock subcomponents that are not ESA listed) and lower river (comprising ESA‐listed lower Columbia and upper Willamette River stock subcomponents).

Although the SCTF performs useful roles to estimate coarse‐level stock characteristics to help Oregon and Washington state managers plan non‐treaty fisheries, the metrics it provides do not include abundance and the information is too coarse to offer details on ESA‐listed stocks and other stock subcomponents that are of interest to a wider audience of regional managers (e.g., managers of fisheries activities above Bonneville dam and hatchery programs distributed across the Columbia River Basin). We examined the following two ways the SCTF's utility for fisheries management could potentially be improved and expanded: (1) utilize a proxy for abundance (catch per unit effort, CPUE) to predict the strength and timing of lower river and upriver stocks in advance of their arrival at upstream destinations and (2) utilize modern tools (genetic analysis) to resolve its coarse scale data into a greater number of stock subcomponents. These two concepts for expanding its utility were used to shape the objectives for this study.

The spring management period fisheries (January 1–June 15) in the mainstem Columbia River are managed by the states (Oregon and Washington) and the Columbia River Inter‐Tribal Fish Commission's member tribes under the *U.S. v OR* Management Agreement. The allocation of “allowable” fish is split 50:50 between the states and the tribes and these numbers of allowed fish are generated from a preseason forecast which gets revised in‐season as more information becomes available to the *U.S. v OR* Technical Advisory Committee (a group that includes state and tribal technical staff who provide information to policy staff who make management decisions for the fisheries). Allowed limits of fish are set based on a rate applied to the run of upriver fish that enter the Columbia River and are destined to pass above Bonneville dam. The spring Chinook harvest rates are affected by the abundance of the upriver run (average 150,485, range 73,101–288,994 in past 10 years) which is combined with the abundance of lower river run to make up the total spring Chinook run (average 227,927, range 110,144–421,411 in past 10 years). Harvest of the upriver stock is typically limited by two stock subcomponents that have ESA protection (natural origin upper Columbia spring Chinook and Snake River spring Chinook). The management goal for the states has been to constrain the fishery with enough buffer to allow fishing opportunity on a portion of their allowed share of spring Chinook, while not exceeding the allowable harvest share for the treaty fishery. The target escapement of spring Chinook through the mainstem fishery is established to provide hatchery programs, tributary fisheries, and natural spawning grounds with sustainable numbers of fish. Therefore, the value of the test fishery could be increased if it were useful in determining whether the strength and timing of the run were in line with preseason forecasts of lower and upriver stocks so the non‐treaty fisheries (mostly recreational hook and line) downstream of Bonneville dam can proceed with a scheduled set of openings that meet their objectives. These objectives include not exceeding the total allowable catch for treaty fisheries that occur mostly upstream of Bonneville dam (typically in the form of platform hook and line and gill net fisheries that provide fish for ceremonial and subsistence use).

The SCTF has ideal characteristics with potentially adequate lead time to produce useful applications for fisheries management. First, this drift net fishery located near the Columbia River mouth intercepts a mixture of “lower” and “upriver” stocks of spring Chinook salmon before they complete migration to one of two major destinations, either to Willamette Falls for the “lower” stock (Oregon City, OR; 196 Rkm upstream of the Columbia River mouth) or to Bonneville dam for the “upriver” stock (235 Rkm). This interception point thus provides data potentially days to weeks in advance depending on travel rates (e.g., range of 18.7–42 km/day for Chinook salmon between dams, Fryer et al., 2019; average of 17.7 days for spring Chinook salmon to travel from Columbia River estuary to Bonneville dam for 2010–2015, Wargo Rub et al., 2019). Second, data collected in this test fishery can be further refined using visual stock identification (VSI), which is a real‐time method of immediately distinguishing spring Chinook salmon into “lower” versus “upriver” stocks (lower river fish have a white belly and upriver fish have a black lower jaw; e.g., Figure S1) that primarily migrate either to the Willamette River or upstream of Bonneville dam, respectively. VSI requires basic routine handling and its stock classifications generally align with information from coded wire tag recoveries well enough to be useful for management applications (e.g., Joint Columbia River Management Report, 2022). VSI can be conducted in minutes and analysis of CPUE data in the test fishery can be used to generate predictions in the same day that the test fishery is executed. Third, similar to the Port Moller Test Fishery (Dann et al., 2013), this SCTF can be enhanced using expedited genotyping analysis to provide high accuracy and resolution of stocks. The genetic analyses, genetic stock identification (GSI) and parentage‐based tagging (PBT), provide an accurate means to identify both the origin of Chinook salmon (hatchery vs. natural origin without relying on adipose clips exclusively, Hargrove et al., 2021) as well as to determine stocks of Chinook salmon at fine‐scale resolution. GSI resolves stocks to the reporting group level (population groups, Hess et al., 2014), while PBT resolves stocks to hatchery groups (“broodstocks,” a brood year of a particular hatchery group) and even to the level of a pair of hatchery parents (Steele et al., 2019). Unlike VSI, utilization of GSI requires steps that would extend the timeline to a point when results can be interpreted to make predictions.

We had the following three objectives in this study: (1) determine the ability of the test fishery to predict abundance and timing of upriver adult Chinook salmon at Bonneville dam, (2) determine concordance between VSI and GSI to distinguish stocks, (3) determine whether integration of PBT/GSI with test fishery CPUE can predict abundance of upriver Chinook salmon stocks at fine spatial scales. Specifically, we used 5 years (2017–2019, 2021–2022) of data to evaluate the level of accuracy, resolution, and advance timing of predictions for the stock‐specific run timing and abundance of spring Chinook salmon arriving at Willamette Falls (“lower” stock) and Bonneville dam (“upriver” stock).

## 2 METHODS

### 2.1 SCTF data collection

**FIGURE 1:**
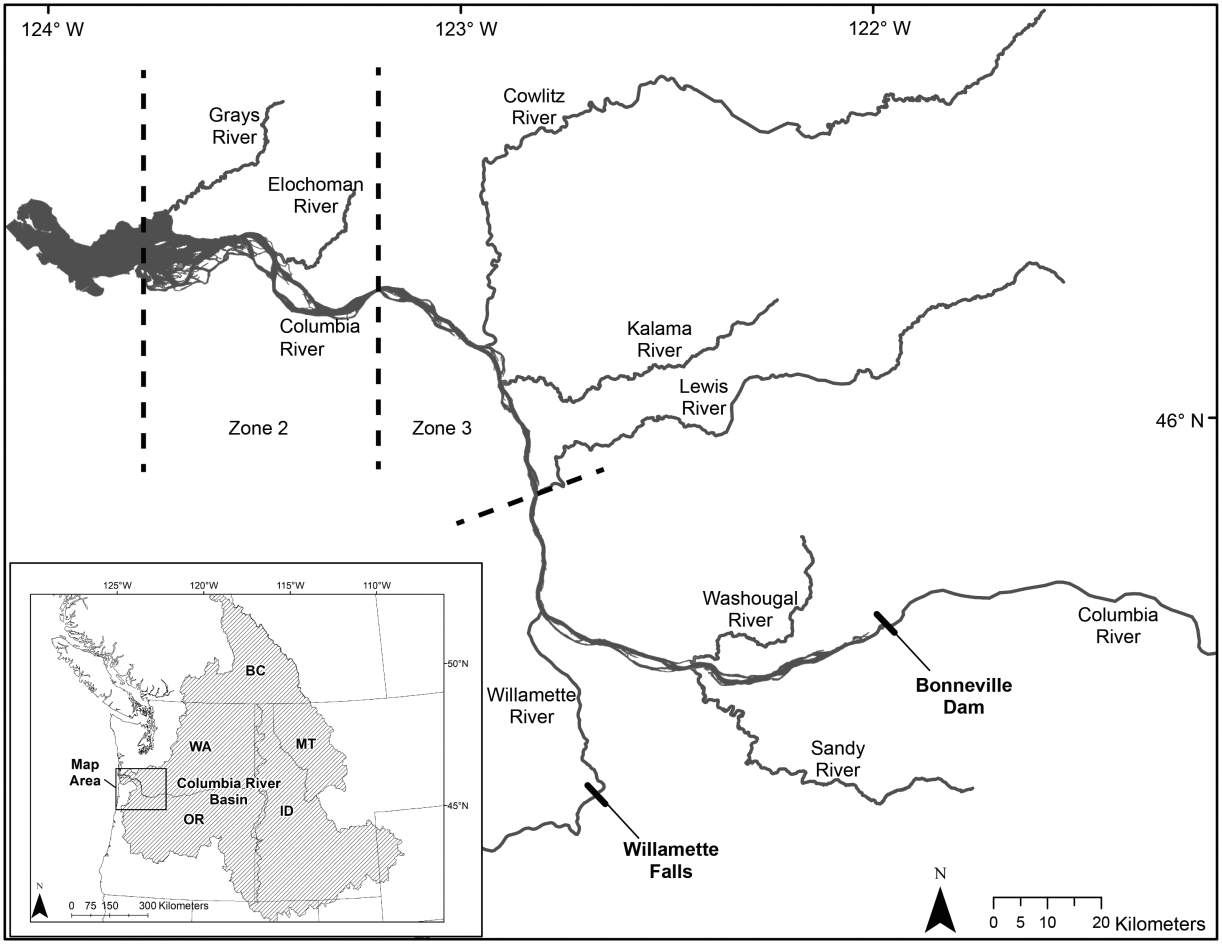
Map of the study area within the Columbia River Basin (inset). The Spring Chinook Salmon Test Fishery is conducted on the mainstem Columbia River within commercial fishing zones 2 and 3. The stock that is visually identified as “lower” will primarily migrate upstream to Willamette Falls and the “upriver” stock will primarily migrate upstream to Bonneville dam.

The SCTF is operated under the guiding fishery management objectives adopted by the Oregon and Washington Fish and Wildlife (ODFW and WDFW) Commissions to provide fisheries managers with information to shape and manage the spring period mainstem fisheries which primarily consist of a series of non‐treaty recreational hook and line openings, but historically also included non‐treaty commercial drift net. spring fisheries. The SCTF comprised a fleet of three contracted commercial fishing vessels that typically execute five drifts within commercial fishing zones 2 and 3 (Figure 1) using 150–175 fathoms (274–320 m) long tangle nets with 4.25 inch (10.8 cm) mesh which is conducted on a single day (Sunday or Monday) on a weekly schedule from as early as February and as late as June during the spring Chinook salmon management period (January 1–June 15). Each drift is typically conducted using a 45‐min soak time (the time elapsed from when the first of the web is deployed into the water until the web is fully retrieved from the water). The number of weeks that test fishing was scheduled increased through the years (4 weeks in 2017 up to 13 weeks in 2022) and is dependent on available funding and data collection needs on a yearly basis. All contracted vessels are required to carry a WDFW or ODFW staff observer during all fishing activities to allow for data collection on fish encountered. Staff observers record information on fishing duration such as net layout and retrieval time and examine all fish captured for several identification characteristics.

VSI is used to categorize Chinook salmon into two major stocks, “lower” river and “upriver” stocks based primarily on coloration distinctions consisting of having either a white belly or black lower jaw for lower and upriver stocks, respectively (Figure S1). Lower and upriver stocks are destined for tributaries below Bonneville dam or above Bonneville dam, respectively. Chinook salmon are also visually assessed for the absence or presence of an adipose fin (i.e., “AD,” adipose clipped; or “AI,” adipose intact) which provides a classification of being either hatchery origin or putatively natural origin, respectively. Chinook salmon were measured to classify them as adult sized (fork length >560 mm, proxy for age classes 4+ years) versus jack sized (fork length ≤560 mm, proxy for age class 3 year; Army Corps of Engineers classifications, https://www.nwp.usace.army.mil/Missions/Environmental‐Stewardship/Fish/Counts/). Harvest management specifies allocation rules primarily for adult Chinook salmon, and so jack‐sized fish were not included in our analyses. Finally, fish were scanned for the presence of coded wire tags (CWT) and passive integrated transponder (PIT) tags, and a small fin clip sample was removed for genetic analysis. Due to time constraints not every biosampled fish in the SCTF collections could be sampled for GSI, but we obtained GSI samples from an average of 85% of all adult‐sized fish captured in the 5 years between 2017 and 2022, excluding 2020 (*n* = 189, 323, 197, 135, and 360, respectively, Table S1). Limited samples were collected during 2020 due to the Covid‐19 pandemic, therefore this year was not included in this study.

The adipose fin status (“AD” and “AI”) for the total number of adult‐sized Chinook salmon captured by drift net was estimated based on the biodata from the sampled fish. Beginning in 2021, numbers of fish observed to be taken from the net by sea lions were also accounted for (average of 30% of samples, Table 1). The biosampled AD adult‐sized Chinook salmon were used to estimate the proportion of AD fish that were VSI lower and VSI upriver stock; these proportions were applied to the total number of AD adult Chinook to estimate total AD‐VSI lower and AD‐VSI upriver fish. These steps were repeated for AI adult Chinook salmon to estimate the total numbers of AI‐VSI lower and AI‐VSI upriver fish. We calculated a catch per unit effort (CPUE) based on the estimated number of adult Chinook salmon captured per drift for each weekly opening of the SCTF. CPUE was used for estimating all categories of adult Chinook salmon including AD and AI split by VSI lower and VSI upriver stocks (Table 1).

**TABLE 1:** Spring Chinook Salmon Test Fishery data (2017–2022).

Year	Week	Month	Day	# of Drifts	Chinook adult data										
					Lower river			Upriver			No VSI			Sea lion	Total
					AD	AI	Unk	AD	AI	Unk	AD	AI	Unk	Take	Chinook
2017	12	March	19	17	14	3	0	10	2	0	0	0	0		29
2017	13	March	26	17	14	1	0	32	8	0	0	0	0		55
2017	14	April	2	17	27	3	0	66	7	0	2	0	0		105
2017	15	April	9	14	14	8	0	75	12	0	0	0	0		109
2018	12	March	18	15	3	0	0	1	0	0	0	0	0		4
2018	13	March	25	17	3	1	0	8	4	0	0	0	0		16
2018	14	April	1	15	4	0	0	8	2	0	0	0	0		14
2018	15	April	8	16	16	2	0	12	6	0	0	0	0		36
2018	16	April	15	16	26	5	0	67	10	0	0	0	0		108
2018	17	April	22	16	40	3	0	73	13	0	0	0	0		129
2018	18	April	29	12	18	3	0	43	13	0	0	0	0		77
2019	12	March	18	15	1	2	0	0	1	0	0	0	0		4
2019	13	March	25	15	4	3	0	4	0	0	0	0	0		11
2019	14	April	1	15	3	2	0	5	2	0	1	0	0		13
2019	15	April	8	15	2	3	0	2	0	0	0	0	0		7
2019	16	April	15	15	27	12	0	26	9	0	0	0	0		74
2019	17	April	21, 22	12	7	2	0	24	9	0	0	0	0		42
2019	18	April	28, 29	14	25	5	0	19	3	0	0	0	0		52
2019	19	May	5, 6	14	7	1	0	6	5	0	0	0	0		19
2021	12	March	15	15	0	0	0	1	0	0	2.00	0.00	2.00	2	3
2021	13	March	22	15	0	0	0	5	1	0	0.83	0.17	1.00	1	7
2021	14	March	29	14	3	0	0	5	1	0	3.56	0.44	4.00	4	13
2021	15	April	5	16	9	0	0	5	1	0	12.13	0.87	13.00	13	28
2021	16	April	11, 12	17	6	2	0	19	5	0	13.28	3.72	17.00	17	49
2021	17	April	18, 19	16	8	1	0	12	6	0	17.04	5.96	23.00	23	50
2021	18	April	25, 26	15	13	5	0	15	3	0	11.67	3.33	15.00	15	51
2021	19	May	2, 3	15	3	0	0	5	3	0	17.45	6.55	24.00	24	35
2021	20	May	9, 10	15	4	2	0	2	1	0	5.33	2.67	8.00	8	17
2022	12	March	14	13	2	0	0	6	1	0	1.78	0.22	2.00	2	11
2022	13	March	21, 22	15	4	0	0	4	1	0	0.89	0.11	1.00	1	10
2022	14	March	28	15	4	0	0	11	2	0	15.88	2.12	18.00	18	35
2022	15	April	5	11	8	1	0	14	3	0	9.31	1.69	11.00	11	37
2022	16	April	11	15	4	0	0	25	7	0	35.44	8.56	44.00	44	80
2022	17	April	18, 19	15	16	3	0	89	14	0	18.07	2.93	21.00	21	143
2022	18	April	25	14	9	0	0	29	4	0	19.90	2.10	22.00	22	64
2022	19	May	2	15	14	2	0	29	6	0	10.12	1.88	12.00	12	63
2022	20	May	9	13	17	2	0	31	7	0	10.11	1.89	12.00	12	69
2022	21	May	16	11	17	2	0	7	8	0	2.12	0.88	3.00	3	37
2022	22	May	22	7	1	0	0	3	0	0	2.00	0.00	2.00	2	6
2022	23	May	29, 31	8	9	0	0	4	1	0	0.00	0.00	0.00	0	14
2022	24	June	9	3	7	0	0	0	0	0	0.00	0.00	0.00	0	7

*Note*: Beginning in 2021, sea lion predation was taken into account by using numbers of fish that were taken by sea lions out of the drift net and breaking those numbers down by AD and AI Chinook salmon based on the biodata from the sampled fish. The drift net mesh size was 4.25 inches and drifts of this Test Fishery were conducted in commercial fishing zones 2 and 3 (Figure 1).

### 2.2 Bonneville dam data collection

Chinook salmon composed of several upriver mixed stocks were nonlethally sampled at the adult fish facility (AFF) located on the northernmost “Washington shore” ladder of Bonneville dam (Figure 1) in the 5 years between 2017 and 2021, excluding 2020, (*n* = 1044, 1433, 1310, 1471, and 1224, respectively). The spring Chinook salmon management period occurs January 1–June 15, and sampling occurred at the AFF usually by April when there were sufficient Chinook salmon numbers that provided a rate of about 1000 fish per day that were counted across all passage routes at Bonneville dam. This delay of the start of sampling each year was estimated to miss <1% of the run of spring Chinook salmon (Hess et al., 2023) and was necessary to use staff time efficiently. The entire run of spring Chinook salmon is enumerated as two categories (adult sized and jack sized, defined previously) passing Bonneville dam on a daily basis using the fish counting windows (https://www.fpc.org). These adult‐sized fish abundance estimates are also split into “AD” and “AI” using weekly samples of fish surveyed by video in the Bonneville dam fish ladders that estimate weekly proportions of each fin clip category; these estimates are made by staff from the *U.S. v OR* Technical Advisory Committee (Table 2).

**TABLE 2:** Stratification of Bonneville dam adult‐sized spring Chinook salmon estimates and adult fish facility sample sizes for adipose‐clipped (AD) and adipose‐intact (AI) Chinook salmon.

Year	Week(s)	Strata	Bonneville dam counts			AFF sample		
			Adult sized			Adult sized		
			AD	AI	Total	AD	AI	Total
2017	1–18	1	17,666	2102	19,768	132	14	146
	19–20	2	21,825	3262	25,087	255	25	280
	21	3	23,625	6098	29,723	129	43	172
	22	4	9676	4175	13,851	123	60	183
	23	5	6692	4213	10,905	80	57	137
	24	6	4862	3328	8190	59	67	126
2018	1–18	1	17,473	2910	20,383	207	56	263
	19	2	20,576	4011	24,587	197	64	261
	20	3	12,988	3397	16,385	168	56	224
	21	4	15,044	5457	20,501	208	104	312
	22	5	5659	2852	8511	79	48	127
	23–24	6	12,050	5628	17,678	162	84	246
2019	1–18	1	13,607	2835	16,442	268	63	331
	19	2	14,090	4357	18,447	212	71	283
	20	3	4715	4822	9537	115	57	172
	21	4	3671	2485	6156	105	61	166
	22	5	3316	1621	4937	66	35	101
	23	6	4922	1618	6540	82	34	116
	24	7	7111	2065	9176	105	36	141
2021	1–17	1	3248	607	3855	100	9	109
	18	2	13,396	2439	15,835	149	29	178
	19	3	12,219	2779	14,998	247	50	297
	20	4	11,593	4422	16,015	171	71	242
	21	5	5093	2728	7821	87	47	134
	22	6	3798	2529	6327	53	40	93
	23	7	5102	2574	7676	100	64	164
	24	8	6527	2937	9464	117	67	184
	25	9	3701	1541	5242	43	27	70
2022	1–18	1	32,634	5353	37,987	192	44	236
	19	2	38,627	7978	46,605	147	44	191
	20	3	19,711	6370	26,081	116	45	161
	21	4	12,299	6123	18,422	90	64	154
	22	5	7514	5432	12,946	80	107	187
	23	6	6949	5360	12,309	76	63	139
	24–25	7	12,036	7351	19,387	94	62	156

The AFF sampled 4–5 days per statistical week (except when reduced due to restrictions on trap use or low run size at the beginning and end of the run) and for 4–6 h per day. A picket weir was used to divert migrating fish ascending the Washington shore fish ladder into the AFF collection pool. An attraction flow was used to draw fish through a false weir where they were selected for sampling. After sampling was completed and fish were recovered from an anesthetic, they were returned to the Washington shore fish ladder above the picket weir. From each fish, caudal fin tissue was taken for genetic analysis and scales for aging; associated metadata, including capture date, fin clip category (“AD” or “AI”), and fork length to the nearest 0.5 cm, were recorded. Fish were categorized by length into adult‐sized and jack‐sized fish as described for the SCTF.

### 2.3 Genetic assignments using GSI and PBT

Tissue samples were dried on Whatman filter paper, and DNA was extracted using the same methods described by Hess et al. (2013) before applying protocols for genotyping‐in‐thousands by sequencing (GT‐seq) custom amplicon methods (Campbell et al., 2015) on an Illumina sequencer. The primers for all GT‐seq loci were published previously and publicly available (Janowitz‐Koch et al., 2019). Genotypes of all individuals were organized using the R package EFGLmh (https://github.com/delomast/EFGLmh/) to create input formats required for all analytical programs used in this study. A baseline of reference collections was compiled from a set of 61 reference collections that were classified into 19 reporting groups to use GSI to assign the most likely reporting group of origin without a minimum threshold for assignment probabilities (observed genetic stock, “GenStock_obs”) using the R package, rubias (https://github.com/eriqande/rubias). “Columbia River Basin Chinook Salmon GSI baseline version 3.1” is the dataset archived on FishGen (http://www.fishgen.net/) that was formatted for rubias and pared down to a set of 176 SNP loci known to have high genotyping success. The baseline consisted of 7081 fish across 61 reference collections and 19 reporting groups that had <10% missing data for this set of loci. This GSI baseline has been shown to provide an average of 85% correct assignment to the 19 GSI reporting groups based on leave‐1‐out analysis (Hasselman et al., 2017; Table S3).

PBT was performed with the several different baselines of different spawn year ranges for the different years of test fishery and Bonneville dam mixture sample data. PBT assignments of offspring to parent pairs (trios) were performed using the program SNPPIT (Anderson, 2012) and the threshold for confident assignments was set to a log of odds (LOD) ≥ 14 which has been shown to minimize false positives and false negatives and achieve high concordance with hatchery records (Hess et al., 2016). Genotyping per locus error rates were assumed to be 0.5%, which is considered conservative given the observed average error rate of 0.2% in our laboratory. Different baselines were required due to the change from SNP markers (92 SNPs in legacy baselines dating back to SY2008, and 254 SNPs available since SY2012 for a portion of collections but as standard for all collections by SY2015). The 2017–2019 “mixture samples” were analyzed together using the legacy baseline “SY2008‐SY2017/18* Combined Snake/Columbia CHNK PBT Hatchery Baseline rev. 7/2020” (http://www.fishgen.net/), which included SY2008–SY2017. For the 2021 mixture sample, we used “SY2014–SY2019 Combined Snake/Columbia CHNK PBT Hatchery Baseline rev. 5/2021” and for the 2022 mixture sample, we used “SY2015–SY2020 Combined Snake/Columbia CHNK PBT Hatchery Baseline rev. 05/2022” (http://www.fishgen.net/). This PBT baseline has been shown to produce >95% accurate assignments with a subset of approximately 100 SNPs from our marker panels using standard PBT methods to identify spring Chinook salmon to parents and genetic stock (Steele et al., 2013).

Mixture samples were analyzed using a window of spawn years that allowed PBT assignments of fish aged 2–7 years, for example, for the mixture from collection year 2022, we used a baseline of parents including SY2015 (age 7) to SY2020 (age 2). PBT broodstocks (hatchery and broodyear) were named using a consistent coding convention and all broodstocks were also categorized into expected reporting groups (“GenStock_exp”) similar to the spatial scales of the GSI baseline and based on the geographic location of the hatchery and the source of genetic stock spawned at the hatchery (Table S2). Every fish that was genotyped had a “GenStock_obs” classification from GSI (i.e., 19 reporting groups, Table S3), and a portion of fish (those with PBT assignments) had a “GenStock_exp” category that was classified by its PBT stock such that, when available, the GenStock was informed by the more accurate PBT information.

### 2.4 SCTF genetic stock abundance estimation

The test fishery obtained sample sizes that ranged from 135 to 360 fish and averaged 240 fish annually, sampled across 4–13 weeks (average 8.2 weeks per year). We stratified each sample year (Table 3) using the following guidelines to pool samples across weeks for consistency: (1) use same strata for both lower and upriver VSI groups, (2) ensure that there were no less than 20 fish for each of the VSI stock strata (lower vs. upriver) and no less than 2 fish per stratum within the low‐abundance AI category of each stock, (3) create as many strata as possible. These guidelines balanced the competing needs for high numbers of strata required to detect temporal variation in stock composition across weeks and high sample numbers per strata to attain accuracy for estimating stock proportions.

**TABLE 3:** Stratification of the Spring Chinook Salmon Test Fishery sample sizes for adipose‐clipped (AD) and adipose‐intact (AI) Chinook salmon in the VSI lower river and upriver stocks.

Year	Strata	Week(s)	VSI: Lower river			VSI: Upriver			Grand
			AD	AI	Total	AD	AI	Total	Total
2017	1	12–13	22	3	25	34	4	38	63
	2	14–15	24	8	32	85	9	94	126
2017 Total			46	11	57	119	13	132	189
2018	1	12–15	26	3	29	25	9	34	63
	2	16	22	5	27	52	7	59	86
	3	17–18	53	3	56	108	10	118	174
2018 Total			101	11	112	185	26	211	323
2019	1	12–16	34	20	54	35	9	44	98
	2	17–19	40	4	44	46	9	55	99
2019 Total			74	24	98	81	18	99	197
2021	1	12–16	25	2	27	43	11	54	81
	2	17–20	19	7	26	21	7	28	54
2021 Total			44	9	53	64	18	82	135
2022	1	12–17	28	4	32	97	26	123	155
	2	18–20	37	4	41	86	19	105	146
	3	21–24	34	2	36	14	9	23	59
2022 Total			99	10	109	197	54	251	360
Grand total			364	65	429	646	129	775	1204

We used parentage assignments, genetic stock assignments, and abundance from total CPUE and automated the estimation of stock‐specific abundance and 90% confidence intervals (based on 1000 bootstraps; *α* = 0.10) using the fishCompTools package in R (https://github.com/delomast/fishCompTools). Similar to the methods for Pacific Lamprey described in Hess et al. (2022, 2023), we used three input files for these stock‐specific abundance estimates (individual sample data with GSI and PBT assignments for each fish, CPUE abundance data, and PBT tag rates). The fishCompTools package (Delomas & Hess, 2021) was used to estimate abundance with the following three hierarchical levels of assignment: PBT assignment in units of broodstock (i.e., hatchery stock + broodyear), “GenStock_obs,” and “GenStock_ exp.” Each broodstock has an associated tag rate (calculated by equation 1 from Hess et al., 2022; Table S2).

Not all hatchery produced fish were tagged (tagging rates ranged from 18% to 100%, Table S2). To account for untagged hatchery‐origin fish, the “spibetr” (Salmonid Prior Information for Balancing Expansions with Tag Rates) function within fishCompTools was used to balance expansion of each PBT assignment by the tag rate by concordantly reducing the assignments of the remaining PBT‐unassigned individuals from the same “GenStock_obs” category. This avoided potentially “double‐counting” (Delomas & Hess, 2021) the abundance of natural‐origin stocks that could have occurred if we had not balanced the expansion of PBT‐assigned fish (hatchery‐origin) using a proportional method for subtraction of PBT‐unassigned (putative natural‐origin fish). In this way we estimated abundance of GenStock (in units of CPUE) for the following six categories of fish defined by VSI‐stock (lower vs. upriver), fin clip (AD vs. AI), and PBT assignment (assigned vs. unassigned): (1) lower river AD (“L‐H,” lower river hatchery clipped), (2) lower river AI with PBT (“L‐HNC,” lower river hatchery no clip), (3) lower river AI PBT‐unassigned (“L‐W,” lower river natural‐origin), (4) upriver AD (“U‐H”), (5) upriver AI with PBT (“U‐HNC”), and (6) upriver AI PBT‐unassigned (“U‐W”). For the hatchery‐clipped (“H”) and hatchery‐unclipped (“HNC”) fish, we also estimated abundance to finer resolution provided by PBT hatchery broodstocks.

### 2.5 Bonneville dam genetic stock abundance estimation

For Bonneville dam, the abundance estimation methods were similar to those described earlier for the SCTF, however the abundance input file in this case was created using the estimates of total adult‐sized Chinook salmon passing Bonneville dam that were in the AD or AI fin clip categories (Table 2). The samples obtained from the adult fish facility representing the AD and AI adult‐sized Chinook salmon were stratified and the general guideline to maintain 100 samples per stratum was used to pool samples across weeks to create as many sample strata as possible.

### 2.6 Predictive ability of the Spring Chinook Salmon Test Fishery for the upriver stock at Bonneville dam

#### 2.6.1 Linear regressions with Bonneville dam data lags

Our first objective was to determine whether trends in VSI upriver stock CPUE in the SCTF could predict the timing and abundance of upriver adult‐sized Chinook salmon at Bonneville dam. We plotted abundance estimates of adult‐sized VSI upriver (CPUE) and weekly sums of estimated abundance of adult Chinook salmon passing Bonneville dam (total counts across fish ladders) by statistical week for each year. Typically, spring Chinook salmon exhibit a single peak in weekly counts at Bonneville dam in early May which approximates when nearly 50% of the run of Chinook salmon will have passed for the spring management period (www.fpc.org). We used the difference in weeks between the statistical week in which we observed a peak in CPUE of VSI upriver adult‐sized Chinook salmon in the SCTF and the statistical week of peak counts of adult‐sized Chinook salmon at Bonneville dam to estimate the average travel time of Chinook salmon between these locations. We lagged the Bonneville dam adult‐sized Chinook salmon counts by this average travel time to fit a linear regression of CPUE of the VSI upriver stock and the Bonneville dam counts. Linear regressions were fit to the fin clip categories “AD,” “AI,” and “total” (i.e., AD and AI combined) for the weekly VSI upriver stock CPUE and Bonneville dam abundance estimates.

#### 2.6.2 Estuary PIT tag recapture study to estimate spring Chinook salmon travel time to Bonneville dam

We estimated travel time to Bonneville dam by using an independent dataset of spring Chinook salmon that were captured and tagged within the Columbia River estuary in the manner of Wargo Rub et al. (2019). These fish were captured by experienced commercial fishers between Rkm 30 and 50 (located at the western boundary of the test fishery zone 2, Figure 1) using a tangle net (4.25‐inch stretch mesh) from March through May. Both hatchery‐clipped (AD) and putatively natural‐origin (AI) adult‐sized Chinook salmon with no visible abnormalities were tagged for this study.

Upon capture, Chinook salmon were placed individually into custom PVC fish tubes and suspended in the river until they could be transferred to a research vessel for sampling and tagging. Once aboard the research vessel, fish were physically restrained in dorsal recumbency using a custom aluminum restraint, measured, scanned for PIT tags, and a pelvic fin clip was obtained for GSI. All untagged fish were injected subcutaneously with a 12‐mm PIT tag (2.0 mm diameter; 0.1 g in air) in the region of the pelvic girdle and the tag id was recorded. Fish identified as having been PIT tagged as juveniles were included in the study without subjecting them to additional tagging.

To estimate survival by reach within the Columbia River below Bonneville dam, a temporally representative subsample of the PIT‐tagged fish was also implanted with 30 MHz VHF radio transmitters (17.0 mm diameter × 44.0 mm length; 14.0 g in air) via gastric insertion using a small ruminant‐sized balling gun. After sampling and tagging, all study fish were placed back into their tubes and held in flow through river water for a minimum of 5 min before being released back into the river to resume their migration. Survival and travel times to Bonneville dam for fish implanted only with PIT tags and those implanted with both a PIT tag and a radio transmitter were compared and found to be similar.

Genotyping of fin tissues was performed with the same marker set and PBT and GSI baseline described earlier, and assignments were also conducted using the same methods described previously. GSI assignments determined whether the fish belonged to upriver versus lower river stock, and the fish with assignments to the upriver stock were used to estimate the average travel time to Bonneville dam (time until first PIT detection at the dam) for each statistical week of capture in the estuary. These estimates allowed visualization of trends in average travel time across the statistical weeks of fish entering the Columbia River at the estuary for 3 years (2017, 2018, and 2021). Fish that had travel data were combined across years (*n* = 212, average 30.3 per week and range 8–69 per week) and allowed estimation of travel time for 7 consecutive statistical weeks (weeks 13–19) with sample sizes of *n* > 5 fish each week.

### 2.7 Concordance of VSI and GSI

#### 2.7.1 VSI versus GSI individual assignments

We compared the classification of fish into lower and upriver stocks using VSI versus GSI. Among the 23 GenStock categories in the GSI and PBT baselines, there are 17 potentially encountered in the spring management period, including three that are classified as lower river stock (01_YOUNGS, 02_WCASSP, and 04_WILLAM), and 14 that are classified as upriver stock (06_KLICKR, 07_DESCSP, 08_JOHNDR, 09_YAKIMA, 10_UCOLSP, 11_TUCANO, 12_HELLSC, 13_SFSALM, 14_CHMBLN, 15_MFSALM, 16_UPSALM, 18_UCOLSF, 20_BONPOOLSP, and 21_UMATILLASP). We calculated the proportion of adult‐sized fish that were determined to be GSI lower stock out of the total number of adult‐sized fish identified as VSI lower stock. Similarly, we calculated the concordance of the GSI and VSI upriver stock for each year. We also calculated the “total” number of fish that were concordantly (VSI and GSI) classified into either lower or upriver stock.

#### 2.7.2 Linear fit of SCTF and Bonneville dam abundance data using VSI versus GSI

We tested whether the level of concordance between VSI and GSI affected the linear fit of the SCTF and Bonneville dam abundance data. Using the SCTF genetic stock abundance estimates, we calculated the total sum of all upriver stock (summed the 14 upriver GSI stocks described earlier) according to AD, AI, and total adult‐sized Chinook salmon in the SCTF for each week and year. These weekly CPUE estimates were then regressed with the Bonneville dam data to generate a similar set of linear regressions that had been produced with VSI‐only classifications of upriver stock: fin clip categories “AD,” “AI,” and “total” (i.e., AD and AI combined) using the same time lag as implemented previously. The linear regressions for the “VSI‐only” CPUE SCTF data were compared to those using GSI based on the *R* ^2^ and slopes of the linear fits.

### 2.8 Predictive ability of the Spring Chinook Salmon Test Fishery at fine resolution of genetic stocks

#### 2.8.1 Upriver GenStock level of resolution

Finally, we used weekly estimates of CPUE for each of the 14 GenStocks within the upriver stock to estimate test the fit linear regressions of the weekly counts of these same stock abundance estimates at Bonneville dam. Linear trends were compared to the level of fit that the coarse level of upriver stock was able to attain in terms of *R* ^2^ and slope.

#### 2.8.2 Upriver Hatchery broodstock level of resolution

We further tested what level of stock resolution was possible to retain good predictive ability by fitting linear relationships of CPUE in the SCTF and Bonneville dam using the broodstock level of the hatchery‐origin stocks (clipped hatchery “H” and unclipped hatchery PBT‐assigned “HNC”).

#### 2.8.3 Lower river GenStock prediction

We examined whether the CPUE of GenStocks identified as lower river stock, specifically the 04_WILLAM stock that is destined primarily for the Willamette River could predict the abundance of spring Chinook salmon in the Willamette River as reported by the dam counts at Willamette Falls (https://myodfw.com/willamette‐falls‐fish‐counts). For this objective, we used the same time lag as had been applied to perform regressions of the upriver stock CPUE in the SCTF and the Bonneville dam counts. Use of the same time lag was an appropriate starting point for comparisons of regressions given the observed travel rates from the estuary to Bonneville dam and the similar distance fish traverse from the estuary to either Bonneville dam (235 Rkm) or Willamette Falls (196 Rkm). We examined three different levels of stocks to estimate CPUE for these regressions: (1) a coarse level using the VSI “lower” stock, (2) a GSI‐based “lower” stock using the combined GenStocks classified as “lower” stock (i.e., 01_YOUNGS, 02_WCASSP, and 04_WILLAM), and (3) only a single GenStock (04_WILLAM). The regressions from these three stock levels were compared (based on slope and *R* ^2^) to the upriver stock regressions described previously.

### 2.9 In‐season management application

Finally, we developed one approach that could utilize the SCTF data on a timely basis in‐season to predict the future abundance and timing of the run. We determined how many data points were required for the slope of a linear regression of CPUE in the SCTF versus Bonneville dam counts to converge for each year of data. For this application, we assumed the same consistent time lag (2 weeks) that would pass between an observed upriver CPUE value and its corresponding weekly count of Chinook salmon at Bonneville dam. We used the first three openings of the SCTF to regress with the first 3 weeks Bonneville dam counts to build the first linear regression; afterward, we added in each subsequent SCTF CPUE data point and its corresponding week of Bonneville dam counts to establish a new linear regression (the datasets of each subsequent linear regression would grow by a single data point). We examined whether years with sufficient data points (>4 test fishery openings, 2018–2022) showed a consistent number of data points were required before the value of the slope of the regression converged with the final slope value of the regression that utilized the total dataset of a given year (“convergence point”).

Once we determined what minimum number of data points were required for the “convergence point,” we used this number of data points to predict the future abundance and timing of the run. We used the minimum number of weekly openings of the SCTF (“convergence point,” known *x* values) to regress with same number of weeks of Bonneville dam counts (i.e., known *y* values) to build the first linear regression; then we interpolated up to three future weeks of abundances (unknown *y* values) using the next three data points (*x* values) of test fishery CPUE (if available; some datasets had fewer total openings than others). We calculated the absolute percentage error (APE) using the predicted cumulative abundances compared to the observed cumulative abundance at Bonneville dam (absolute value of (observed − predicted)/observed × 100) and averaged APE across years (MAPE).

## 3 RESULTS

### 3.1 SCTF GSI/PBT assignments and CPUE estimates

The calculations of CPUE in the SCTF varied by week, year, VSI stock, and fin clip status (Table 4). The genetic analysis of samples for each collection year provided individual assignments for reporting groups (GenStock, Table 5) and for the PBT assigned fish the GenStock could be further divided into hatchery broodstocks (Table S4).

**TABLE 4:** The estimated catch per unit effort (CPUE) of visually stock‐identified (VSI) lower river and upriver adult Chinook salmon stocks caught in the Spring Chinook Salmon Test Fishery split into adipose‐clipped (AD) and adipose‐intact (AI) fin‐clip categories.

		Lower river							Upriver						
		AD			AI			Total	AD			AI			Total
Year	Week	%	Est. total	CPUE	%	Est. total	CPUE	CPUE	%	Est. total	CPUE	%	Est. total	CPUE	CPUE
2017	12	0.58	14.00	0.82	0.60	3.00	0.18	1.00	0.42	10.00	0.59	0.40	2.00	0.12	0.71
2017	13	0.30	14.00	0.82	0.11	1.00	0.06	0.88	0.70	32.00	1.88	0.89	8.00	0.47	2.35
2017	14	0.29	27.58	1.62	0.30	3.00	0.18	1.80	0.71	67.42	3.97	0.70	7.00	0.41	4.38
2017	15	0.16	14.00	1.00	0.40	8.00	0.57	1.57	0.84	75.00	5.36	0.60	12.00	0.86	6.21
2018	12	0.75	3.00	0.20	0.00	0.00	0.00	0.20	0.25	1.00	0.07	0.00	0.00	0.00	0.07
2018	13	0.27	3.00	0.18	0.20	1.00	0.06	0.24	0.73	8.00	0.47	0.80	4.00	0.24	0.71
2018	14	0.33	4.00	0.27	0.00	0.00	0.00	0.27	0.67	8.00	0.53	1.00	2.00	0.13	0.67
2018	15	0.57	16.00	1.00	0.25	2.00	0.13	1.13	0.43	12.00	0.75	0.75	6.00	0.38	1.13
2018	16	0.28	26.00	1.63	0.33	5.00	0.31	1.94	0.72	67.00	4.19	0.67	10.00	0.63	4.81
2018	17	0.35	40.00	2.50	0.19	3.00	0.19	2.69	0.65	73.00	4.56	0.81	13.00	0.81	5.38
2018	18	0.30	18.00	1.50	0.19	3.00	0.25	1.75	0.70	43.00	3.58	0.81	13.00	1.08	4.67
2019	12	1.00	1.00	0.07	0.67	2.00	0.13	0.20	0.00	0.00	0.00	0.33	1.00	0.07	0.07
2019	13	0.50	4.00	0.27	1.00	3.00	0.20	0.47	0.50	4.00	0.27	0.00	0.00	0.00	0.27
2019	14	0.38	3.38	0.23	0.50	2.00	0.13	0.36	0.63	5.63	0.38	0.50	2.00	0.13	0.51
2019	15	0.50	2.00	0.13	1.00	3.00	0.20	0.33	0.50	2.00	0.13	0.00	0.00	0.00	0.13
2019	16	0.51	27.00	1.80	0.57	12.00	0.80	2.60	0.49	26.00	1.73	0.43	9.00	0.60	2.33
2019	17	0.23	7.00	0.58	0.18	2.00	0.17	0.75	0.77	24.00	2.00	0.82	9.00	0.75	2.75
2019	18	0.57	25.00	1.79	0.63	5.00	0.36	2.14	0.43	19.00	1.36	0.38	3.00	0.21	1.57
2019	19	0.54	7.00	0.50	0.17	1.00	0.07	0.57	0.46	6.00	0.43	0.83	5.00	0.36	0.79
2021	12	0.00	0.00	0.00	0.00	0.00	0.00	0.00	1.00	3.00	0.20	0.00	0.00	0.00	0.20
2021	13	0.00	0.00	0.00	0.00	0.00	0.00	0.00	1.00	5.83	0.39	1.00	1.17	0.08	0.47
2021	14	0.38	4.33	0.31	0.00	0.00	0.00	0.31	0.63	7.22	0.52	1.00	1.44	0.10	0.62
2021	15	0.64	16.80	1.05	0.00	0.00	0.00	1.05	0.36	9.33	0.58	1.00	1.87	0.12	0.70
2021	16	0.24	9.19	0.54	0.29	3.06	0.18	0.72	0.76	29.09	1.71	0.71	7.66	0.45	2.16
2021	17	0.40	14.81	0.93	0.14	1.85	0.12	1.04	0.60	22.22	1.39	0.86	11.11	0.69	2.08
2021	18	0.46	18.42	1.23	0.63	7.08	0.47	1.70	0.54	21.25	1.42	0.38	4.25	0.28	1.70
2021	19	0.38	9.55	0.64	0.00	0.00	0.00	0.64	0.63	15.91	1.06	1.00	9.55	0.64	1.70
2021	20	0.67	7.56	0.50	0.67	3.78	0.25	0.76	0.33	3.78	0.25	0.33	1.89	0.13	0.38
2022	12	0.25	2.44	0.19	0.00	0.00	0.00	0.19	0.75	7.33	0.56	1.00	1.22	0.09	0.66
2022	13	0.50	4.44	0.30	0.00	0.00	0.00	0.30	0.50	4.44	0.30	1.00	1.11	0.07	0.37
2022	14	0.27	8.24	0.55	0.00	0.00	0.00	0.55	0.73	22.65	1.51	1.00	4.12	0.27	1.78
2022	15	0.36	11.38	1.03	0.25	1.42	0.13	1.16	0.64	19.92	1.81	0.75	4.27	0.39	2.20
2022	16	0.14	8.89	0.59	0.00	0.00	0.00	0.59	0.86	55.56	3.70	1.00	15.56	1.04	4.74
2022	17	0.15	18.75	1.25	0.18	3.52	0.23	1.48	0.85	104.32	6.95	0.82	16.41	1.09	8.05
2022	18	0.24	13.71	0.98	0.00	0.00	0.00	0.98	0.76	44.19	3.16	1.00	6.10	0.44	3.59
2022	19	0.33	17.29	1.15	0.25	2.47	0.16	1.32	0.67	35.82	2.39	0.75	7.41	0.49	2.88
2022	20	0.35	20.58	1.58	0.22	2.42	0.19	1.77	0.65	37.53	2.89	0.78	8.47	0.65	3.54
2022	21	0.71	18.50	1.68	0.20	2.18	0.20	1.88	0.29	7.62	0.69	0.80	8.71	0.79	1.48
2022	22	0.25	1.50	0.21	0.00	0.00	0.00	0.21	0.75	4.50	0.64	0.00	0.00	0.00	0.64
2022	23	0.69	9.00	1.13	0.00	0.00	0.00	1.13	0.31	4.00	0.50	1.00	1.00	0.13	0.63
2022	24	1.00	7.00	2.33	0.00	0.00	0.00	2.33	0.00	0.00	0.00	0.00	0.00	0.00	0.00

*Note*: “Week” is the statistical week that the test fishery was conducted (typically a Sunday or Monday, which is the first or second day of the statistical week). Percent of the sample (“%”) is shown as a proportion of all estimated AD clipped fish that were either lower or upriver; and proportion of all AI fish that were either lower or upriver. Catch per unit effort (“CPUE”) is the number of adult Chinook salmon caught per drift.

**TABLE 5:** Spring Chinook Salmon Test Fishery assignments to Genetic Stock “GenStock” (2017–2022).

	GenStock	2017							2018							2019						
		Lower (VSI)			Upriver (VSI)			Total	Lower (VSI)			Upriver (VSI)			Total	Lower (VSI)			Upriver (VSI)			Total
		H	HNC	W	H	HNC	W		H	HNC	W	H	HNC	W		H	HNC	W	H	HNC	W	
Lower	01_YOUNGS							0							0				1			1
	02_WCASSP	26			10			36	6		1	8			15	9			2			11
	03_WCASFA							0							0							0
	04_WILLAM	15		4	9		3	31	61		7	16			84	48		19	10		2	79
	Subtotal lower GenStock	41	0	4	19	0	3	67	67	0	8	24	0	0	99	57	0	19	13	0	2	91
	05_SPCRTU							0							0							0
Upriver	06_KLICKR	1						1	2			1			3	1						1
	07_DESCSP				2		1	3	3			3		3	9				3			3
	08_JOHNDR							0						1	1							0
	09_YAKIMA			2	1		4	7			1	4		3	8	2			9		4	15
	10_UCOLSP	1		2	12		1	16	4			11	2	4	21	5	1	1	7		5	19
	11_TUCANO					1		1					2		2			2				2
	12_HELLSC	3	2		54	2	1	62	11	1		66	4	3	85	4		1	29	3	3	40
	13_SFSALM				1			1			1	2			3				1			1
	14_CHMBLN							0							0							0
	15_MFSALM							0							0						1	1
	16_UPSALM				1			1				7			7				2			2
	17_DESCFA							0							0							0
	18_UCOLSF							0							0							0
	19_SRFALL							0							0							0
	20_BONPOOLSP		1		14			15	13			53	2		68	5			16			21
	21_UMATILLASP				15			15	1			14	2		17				1			1
	Subtotal upriver GenStock	5	3	4	100	3	7	122	34	1	2	161	12	14	224	17	1	4	68	3	13	106
	Concordance	78.9%			83.3%			82.0%	67.0%			88.6%			81.1%	77.6%			84.8%			81.2%

	GenStock	2021							2022							Grand total						
		Lower (VSI)			Upriver (VSI)			Total	Lower (VSI)			Upriver (VSI)			Total	Lower (VSI)			Upriver (VSI)			Total
		H	HNC	W	H	HNC	W		H	HNC	W	H	HNC	W		H	HNC	W	H	HNC	W	
Lower	01_YOUNGS							0							0	0	0	0	1	0	0	0
	02_WCASSP	3		1	4			8	16			10			26	60	0	2	34	0	0	85
	03_WCASFA							0							0	0	0	0	0	0	0	0
	04_WILLAM	22		1			1	24	65		8	9		2	84	211	0	39	44	0	8	223
	Subtotal lower GenStock	25	0	2	4	0	1	32	81	0	8	19	0	2	110	271	0	41	79	0	8	308
	05_SPCRTU							0							0	0	0	0	0	0	0	0
Upriver	06_KLICKR	1			1			2				4			4	5	0	0	6	0	0	10
	07_DESCSP	1			3	1		5				5			5	4	0	0	16	1	4	22
	08_JOHNDR			1	1			2							0	0	0	1	1	0	1	3
	09_YAKIMA	1		1			2	4	1		1	6		3	11	4	0	5	20	0	16	30
	10_UCOLSP	7	3	1	15	1	1	28	1			29	4	12	46	18	4	4	74	7	23	111
	11_TUCANO							0							0	0	0	2	0	3	0	3
	12_HELLSC	3			20	5	1	29	6		1	85	8	13	113	27	3	2	254	22	21	289
	13_SFSALM			1			1	2				10	2	3	15	0	0	2	14	2	4	21
	14_CHMBLN							0							0	0	0	0	0	0	0	0
	15_MFSALM						1	1						2	2	0	0	0	0	0	4	3
	16_UPSALM	1			1			2				10	1	2	13	1	0	0	21	1	2	23
	17_DESCFA							0							0	0	0	0	0	0	0	0
	18_UCOLSF							0	6						6	6	0	0	0	0	0	6
	19_SRFALL							0							0	0	0	0	0	0	0	0
	20_BONPOOLSP				11			11	4			28	1		33	22	1	0	122	3	0	127
	21_UMATILLASP				1	1		2				1	1		2	1	0	0	32	4	0	36
	Subtotal upriver GenStock	14	3	4	53	8	6	88	18	0	2	178	17	35	250	88	8	16	560	43	75	684
	Concordance	56.3%			93.1%			78.3%	81.7%			91.6%			88.6%	73.6%			88.6%			83.3%

*Note*: Shaded “GenStock” names are reporting groups with run timing that does not typically overlap the spring management period and are expected to have minimal contribution to the upriver spring stock.

Using the genetic assignments to GenStock, we estimated the CPUE by breaking down the VSI stock (lower vs. upriver) in the six categories: L‐H, L‐HNC, L‐W, U‐H, U‐HNC, U‐W (Table S5). All CPUE estimates for GenStock belonging to one of the lower river stocks were summed into one total lower river GenStocks (Table S5), and similarly, a total sum was calculated for all upriver GenStocks so that they could be directly compared to the CPUE estimates generated by VSI (Table 4). The “H” and “HNC” groups were hatchery‐clipped and hatchery‐no‐clip groups that could more finely be broken down to estimate CPUE for each of the broodstocks based on the individual assignments (Table S4).

### 3.2 Bonneville dam adult‐sized GSI/PBT assignments and abundance estimates

The run size varied by year and fin clip status for estimated numbers of adult‐sized spring Chinook salmon passing Bonneville dam (Table 2). The genetic analysis of samples at the adult fish facility each year allowed further division of AD and AI adult‐sized Chinook salmon into individual assignments to GenStock (Table S6) and hatchery broodstocks (Table S7). The GenStock abundance estimation at Bonneville dam provided H, HNC, and W estimates of each GenStock (Table 6), and the H and HNC categories were further split into hatchery broodstocks (Table S8).

**TABLE 6:** Estimated abundance of adult‐sized Chinook Salmon passing Bonneville dam (2017–2022) in units of genetic stock (“GenStock”).

	GenStock	2017				2018				2019			
		H	HNC	W	Total	H	HNC	W	Total	H	HNC	W	Total
Lower	01_YOUNGS	0		0	0	76		0	76	0		0	0
	02_WCASSP	836		140	976	2126	55	0	2181	825	43	0	867
	03_WCASFA	0		130	130			0	0	0		0	0
	04_WILLAM	239		130	370	203		0	203	312	0	0	312
	Subtotal lower GenStock	1075	0	401	1476	2406	55	0	2461	1137	43	0	1179
	05_SPCRTU	0	0	0	0	0	0	0	0	0	0	0	0
Upriver	06_KLICKR	3054		279	3334	820		52	873	124		0	124
	07_DESCSP	7096	335	130	7561	1915	261	596	2772	1314	109	477	1900
	08_JOHNDR	0		499	499			567	567	0		474	474
	09_YAKIMA	2622		2494	5115	3159		2124	5283	775	42	884	1700
	10_UCOLSP	5506	561	1610	7676	5167	1189	2582	8938	5510	2272	2513	10,295
	11_TUCANO	0	564	0	564		498	163	661	0	268	91	359
	12_HELLSC	29,584	1756	2413	33,754	32,047	2285	5173	39,505	19,024	1596	4142	24,762
	13_SFSALM	3333	1261	486	5080	2673	718	1276	4667	2054	799	1054	3907
	14_CHMBLN	0		0	0			225	225	0		0	0
	15_MFSALM	0		300	300			644	644	0		308	308
	16_UPSALM	3127	50	1096	4273	7072	329	632	8033	1724		880	2604
	17_DESCFA	0		0	0	0		0	0	0		0	0
	18_UCOLSF	8121	409	7089	15,619	9242	782	2801	12,825	12,709	483	2300	15,492
	19_SRFALL	157	0	143	300	73	0	65	138	0	0	105	105
	20_BONPOOLSP	12,975	888		13,863	12,510	564	0	13,074	6781	184	0	6965
	21_UMATILLASP	7696	414		8110	6706	671	0	7377	281	779	0	1060
	Subtotal upriver GenStock	83,270	6238	16,540	106,048	81,384	7297	16,902	105,584	50,296	6531	13,229	70,056

	GenStock	2021				2022			
		H	HNC	W	Total	H	HNC	W	Total
Lower	01_YOUNGS	0	0	0	0	0	0	0	0
	02_WCASSP	530	0	0	530	2615	235	0	2850
	03_WCASFA	0	0	0	0	0	0	0	0
	04_WILLAM	52	0	0	52	9	0	378	388
	Subtotal lower GenStock	582	0	0	582	2624	235	378	3237
	05_SPCRTU	0	0	0	0	0	0	0	0
Upriver	06_KLICKR	1230	0	121	1351	1772	123	237	2132
	07_DESCSP	4388	201	275	4864	5761	0	771	6532
	08_JOHNDR	44	0	267	311	0	0	484	484
	09_YAKIMA	1258	103	1544	2905	2055	121	1764	3940
	10_UCOLSP	7291	3129	2068	12,488	18,126	3666	5777	27,569
	11_TUCANO	0	0	0	0	0	0	122	122
	12_HELLSC	24,204	2383	2277	28,865	49,901	4253	8118	62,273
	13_SFSALM	3580	1163	1000	5743	3390	743	3539	7672
	14_CHMBLN	0	0	98	98	9	0	350	359
	15_MFSALM	0	0	588	588	0	0	2140	2140
	16_UPSALM	1282	499	1457	3238	5857	538	2522	8918
	17_DESCFA	0	0	0	0	0	0	0	0
	18_UCOLSF	12,250	936	3933	17,118	13,724	1630	4591	19,944
	19_SRFALL	0	0	44	44	0	0	0	0
	20_BONPOOLSP	7015	301		7316	22,423	1238		23,661
	21_UMATILLASP	1553	168		1721	4126	630		4756
	Subtotal upriver GenStock	64,097	8882	13,673	86,651	127,145	12,942	30,413	170,500

*Note*: Shaded “GenStock” names are reporting groups with run timing that does not typically overlap the spring management period and are expected to have minimal contribution to the upriver spring stock. Blank cells are reporting groups that had no representation in any of the samples.

### 3.3 Predictive ability of the Spring Chinook Salmon Test Fishery for the upriver stock at Bonneville dam

#### 3.3.1 Linear regressions with Bonneville dam data lags

**FIGURE 2:**
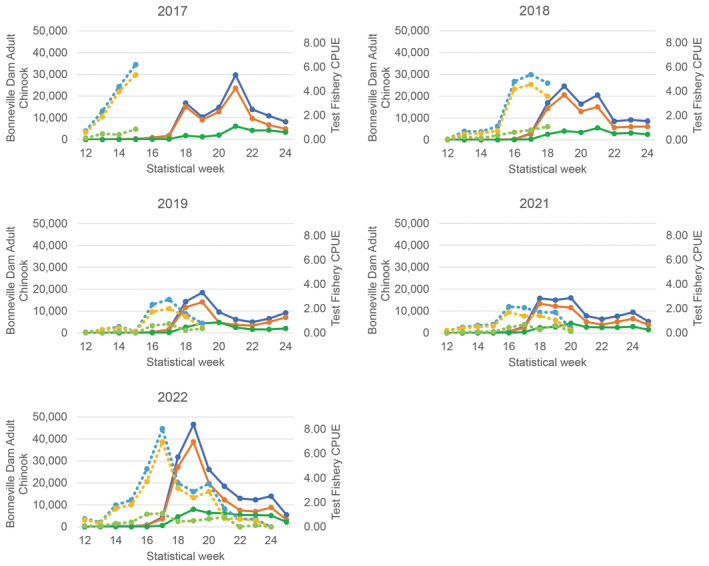
The catch per unit effort (CPUE, adult sized Chinook salmon caught per drift) of visual stock identified (VSI) upriver fish (secondary *y*‐axis) and estimated abundance of adult‐sized Chinook salmon passing Bonneville dam (primary *y*‐axis) plotted by statistical week for years 2017–2022. The adipose‐clipped (AD), adipose‐intact (AI), and “total” Chinook (AD + AI) are represented by line colors of orange, green, and blue, respectively, for the test fishery (dashed) and Bonneville dam counts (solid).

The VSI upriver stock showed a high primary peak in CPUE (total = AD + AI upriver stock) in the SCTF consistently on statistical week 17 for four of the years (2018, 2019, 2021, and 2022), and a peak on week 15 for the year 2017 (Figure 2). The initial peak of upriver CPUE in 2021 was on statistical week 16, but plateaued through week 17. The highest peaks in CPUE each year were higher than 2.0 VSI upriver adult‐sized Chinook salmon per drift in the SCTF and the highest peaks in adult‐sized counts were greater than 15,000 total Chinook salmon at Bonneville dam. All years excluding 2017 showed that the initial peak in CPUE of VSI upriver stock was consistently 2 weeks prior to the initial peak in adult‐sized Chinook salmon at Bonneville dam (Figure 2). For 2017, the peak in CPUE of the upriver stock in the SCTF on week 15 was separated by 3 weeks from an initial peak (week 18) in adult‐sized Chinook salmon at Bonneville dam and separated by 7 weeks from the highest peak (week 21).

We regressed the weekly VSI upriver stock CPUE with the adult‐sized Chinook salmon counts using a 2‐week lag for all years (Figure S2). For the four most recent years (2018–2022) these regressions of “total” Chinook salmon (i.e., AD + AI) had slopes that ranged from 4317 to 7877 adult‐sized Chinook salmon counted at Bonneville dam per 1.0 CPUE of upriver stock in the SCTF (average regression 6191). For 2017, the slope of the regression (288) was an order of magnitude smaller than the average for the recent 4 years. For all years, the *R* ^2^ of the regressions were high (average *R* ^2^ = 0.90, range 0.81–0.97). The regressions for AD Chinook salmon were similar to “total” (average slope = 6394, range 4215–9051; average *R* ^2^ = 0.90, range 0.84–0.97). However, for AI Chinook salmon, the regressions had lower slopes and *R* ^2^ (average slope = 4602, range 4007–5246; average *R* ^2^ = 0.60, range 0.41–0.85).

#### 3.3.2 Estuary PIT tag recapture study to estimate spring Chinook salmon travel time to Bonneville dam

**FIGURE 3:**
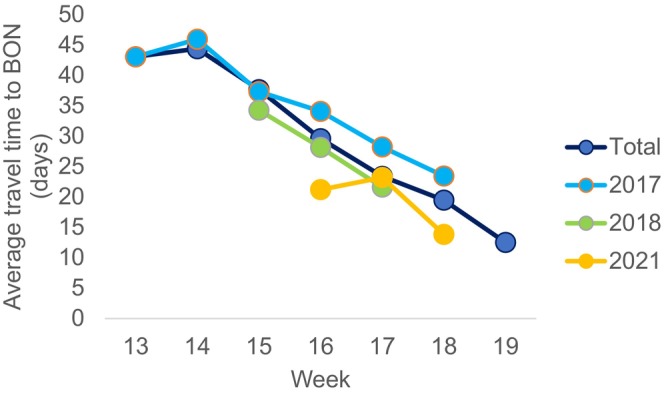
Estuary PIT‐tag mark recapture to estimate travel times to Bonneville dam (2017–2021).

There were enough fish that had travel data once combined across years (*n* = 212, average 30.3 per week and range 8–69 per week) to allow estimation of average travel time for 7 consecutive statistical weeks (weeks 13–19). Average travel time was observed to range from 12.5 to 44.4 days (mean of 30.0 days averaged across the 7 weeks). Average travel time decreased steadily across weeks (Figure 3), such that, in the first two statistical weeks (13–14) average travel time was above 40 days, by statistical week 17 travel time was 23.4 days, and by week 19 travel time was lowest at 12.5 days. Although travel time exhibited a consistent decreasing trend across weeks within each year, the 2017 recapture data exhibited the slowest travel times for any given week (Figure 3).

Because average travel time was observed to decrease across weeks, the week 15 in which CPUE was observed to peak in the SCTF for the year 2017 was expected to have a slower average travel time (37.7 days) compared to the week 17 (average travel time 23.4), which was the peak CPUE for the recent 4 years. The difference in travel time between weeks 17 and 15 was 14.3 days (2 weeks). Therefore, we revised the regression of the 2017 data by extending the lag time by an additional 2 weeks (total lag = 4 weeks; Figure S3) and observed slopes of the linear trend that increased by an order of magnitude (total slope = 2381; AD slope = 2497; AI slope = 1295), but with decreased *R* ^2^ values (total *R* ^2^ = 0.57; AD *R* ^2^ = 0.61; AI *R* ^2^ = 0.24).

Fish must travel up to 200 Rkm after passing through the test fishery (downstream point of the test fishing area is located at Rkm 34 on the Columbia River) to arrive at Bonneville dam which is located at Rkm 235. The estuary PIT tagging study estimated that average travel time to Bonneville dam during week 17 which coincides with the typical peak CPUE in upriver spring Chinook salmon is 23.4 days. Therefore, at the peak of the run through the test fishery the travel rate of these fish was estimated to be 201 Rkm/23.4 days = 8.6 Rkm/day.

### 3.4 Concordance of VSI and GSI

#### 3.4.1 VSI versus GSI individual assignments

The concordance between VSI and GSI for identification of adult‐sized lower river stock was 73.6% for all 5 years combined (Table 5, range 56.3%–81.7% concordance range across years). The concordance between VSI and GSI for identification of adult‐sized upriver stock was 88.6% for all 5 years combined (range 83.3%–93.1% concordance range across years), and total concordance between VSI and GSI was 83.3% for all 5 years combined (range 78.3%–88.6% across years). The year in which overall concordance was lowest was in 2021 (78.3%), which was largely affected by the extremely low concordance for the lower river stock (56.3%).

#### 3.4.2 Linear fit of SCTF and Bonneville dam abundance data using VSI versus GSI

Given that the concordance between VSI and GSI was less than 100%, we expected that the regressions using VSI data compared to GSI data would produce slopes and *R* ^2^ that differ between methods. However, GSI data produced similar results (GSI average *R* ^2^ = 0.91; range 0.80–0.97) compared to VSI (average *R* ^2^ = 0.90; range 0.81–0.97) based on the linear *R* ^2^ of “total” Chinook salmon which did not differ in a consistent way between methods (Figure S2). There were lower slopes for “total” and “AD” Chinook salmon using GSI compared to VSI (e.g., recent 4‐year average of 5897 vs. 6191 for “total” adult‐sized Chinook salmon using GSI vs. VSI, respectively); however, the AI Chinook salmon recent 4‐year average slope was slightly higher using GSI versus VSI (GSI slope = 4801; VSI slope = 4602; Figure S2). Although GSI did not appear to generally influence the fit of the linear regressions compared to VSI, the year 2021 that had shown the lowest level of concordance between VSI and GSI was the only year in which GSI had much greater *R* ^2^ compared to VSI (Figure S2).

### 3.5 Predictive ability of the Spring Chinook Salmon Test Fishery at fine resolution of genetic stocks

#### 3.5.1 Upriver GenStock level of resolution

The linear trends for weekly estimates of CPUE for upriver GenStocks versus their 2‐week‐lagged stock abundance at Bonneville dam were relatively good fits for the data based on high *R* ^2^ (Figure S4). For all years, the *R* ^2^ of the regressions were high (average *R* ^2^ = 0.84, range 0.78–0.89). Furthermore, the slopes of these linear relationships had overlapping ranges with the linear trends observed for the coarse level of upriver stock. For example, for the four most recent years (2018–2022) these regressions of “total” Chinook salmon (i.e., AD + AI) had slopes that ranged from 3783 to 6499 adult‐sized Chinook salmon counted at Bonneville dam per 1.0 CPUE of upriver stock in the SCTF (average slope = 5188). This average slope was smaller than the average slope for the coarse level of upriver stock (average 6053; Figure S2). Similar to the patterns observed for the coarse level of upriver stock, the year 2017 of data exhibited a linear slope of 242, which was an order of magnitude smaller than the average for the recent 4 years.

#### 3.5.2 Upriver Hatchery broodstock level of resolution

The linear trends for weekly estimates of CPUE for upriver hatchery‐clipped broodstocks maintained good fits to the Bonneville dam stock abundance data based on moderate levels of *R* ^2^ (Figure S5). For all years, the *R* ^2^ of the regressions averaged 0.69 (*R* ^2^ ranged 0.55–0.86). As observed for the GenStock level, the slopes of these linear relationships had overlapping ranges with the linear trends observed for the coarse level of upriver stock. For example, for the four most recent years (2018–2022), these regressions of hatchery‐clipped broodstocks had slopes that ranged from 3272 to 5561 adult‐sized Chinook salmon counted at Bonneville dam per 1.0 CPUE of upriver stock in the SCTF (average slope = 4428). This average slope was smaller than the average slope for the coarse level of upriver stock (average 6053; Figure S2) and the GenStock level of upriver stock (average 5188; Figure S4). Similar for the coarse level of upriver stock, the year 2017 of data exhibited a linear slope of 280 which was an order of magnitude smaller than the average for the recent 4 years.

#### 3.5.3 Lower river GenStock prediction

For the “lower” river stock, we used the same time lags as had been identified to be best fits for Bonneville dam data; that is, the time lag was 4 weeks for 2017 and for all other years we used a 2‐week time lag which appeared very similar to the time difference in weeks between the peak in lower river CPUE and the counts of adult Chinook salmon at Willamette Falls (Figure S6). Across all years (2017–2022), the average slope for “total” Chinook salmon (i.e., AD + AI) was 2106 (range 890–2943) adult‐sized Chinook salmon counted at Willamette Falls per 1.0 CPUE of lower stock in the SCTF based on VSI (Figure S7). Using GSI, this average across years for “total” Chinook salmon was 2318 (range 1292–4300); and based on the GenStock 04_WILLAM, the average slope across years for “total” Chinook salmon was 2900 (range 1380–5117). The average (and range) of *R* ^2^ of these regressions was similar across methods: 0.62 (VSI, range 0.19–0.95), 0.66 (GSI, range 0.29–0.96), and 0.64 (GenStock, range 0.26–0.93). The regressions for AD Chinook salmon were similar to the regressions for “total” Chinook salmon (Figure S7), however, for AI Chinook the regressions had lower slopes and *R* ^2^ (e.g., GenStock average slope = 1797, range 189–3894; average *R* ^2^ = 0.19, range 0.01–0.36).

### 3.6 In‐season management application

**FIGURE 4:**
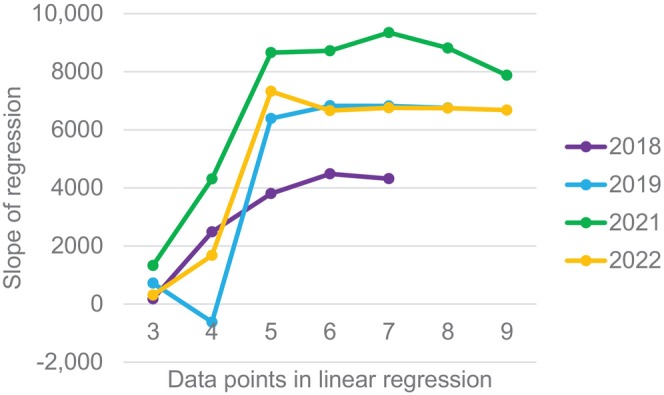
Convergence of the slopes of linear regressions with increasing data points. The slope values approximate the number of adult‐sized Chinook salmon counted at Bonneville dam per 1 upriver VSI adult Chinook caught per drift in the Spring Chinook Salmon Test Fishery.

Finally, we developed one approach that could utilize the SCTF data on a timely basis in‐season to predict the future abundance and timing of the run at Bonneville dam. We determined that five data points were required before the slope values reached convergence with the final linear trend of the CPUE and Bonneville dam counts for each year (Figure 4). We used the first five openings of the SCTF to regress with the first 5 weeks Bonneville dam counts to build the first linear regression; then we predicted up to three future weeks of abundances using the next three data points of test fishery CPUE. The mean absolute percent error (MAPE) for the cumulative predicted abundances using this method was 21.6% (range 3.0–99.6%, Table 7). The earliest predicted abundances (using 5 or 6 data points) had a MAPE of 12.4% (range 3.0–23.6%, Table 7).

**TABLE 7:** In‐season management application for predicting abundance of spring Chinook salmon passing Bonneville dam based on results from the test fishery.

Year	Data points	Slope	*R* ^2^	Predicted slope				Observed counts				Abs error
				Week 0	Week 1	Week 2	Cumulative	Week 0	Week 1	Week 2	Cumulative	
2017	3	4458.9	0.84									
	4	2380.6	0.57									
2018	3	180.8	0.32									
	4	2486.3	0.58									
	5	3803.0	0.98	18,903	16,209		35,111	24,587	16,385		40,972	14.3
	6	4483.1	0.97	18,885			18,885	16,385			16,385	15.3
	7	4316.6	0.96									
2019	3	719.8	0.64									
	4	−620.7	0.03									
	5	6390.2	0.95	16,627	9096	4075	29,797	18,447	9537	6156	34,140	12.7
	6	6827.6	0.98	9645	4280		13,925	9537	6156		15,693	11.3
	7	6820.7	0.98	4267			4267	4937			4937	13.6
	8	6754.5	0.97									
2021	3	1328.6	0.79									
	4	4311.5	0.56									
	5	8658.1	0.97	14,777	11,458	11,432	37,666	14,998	16,015	7821	38,834	3.0
	6	8721.0	0.98	11,536	11,510	5	23,052	16,015	7821	6327	30,163	23.6
	7	9348.2	0.94	12,503	171		12,675	7821	6327		14,148	10.4
	8	8814.2	0.90	27			27	6327			6327	99.6
	9	7876.9	0.81									
2022	3	308.4	0.91									
	4	1675.5	0.62									
	5	7324.6	0.87	52,216	19,572	14,375	86,164	46,605	26,081	18,422	91,108	5.4
	6	6661.9	0.95	18,223	13,496	17,867	49,586	26,081	18,422	12,946	57,449	13.7
	7	6759.0	0.93	14,602	19,037		33,638	18,422	12,946		31,368	7.2
	8	6745.6	0.92	19,507			19,507	12,946			12,946	50.7
	9	6678.2	0.90									

*Note*: Week 0 is the week in which the test fishery has partial but incomplete Bonneville dam count data and subsequent weeks 1 and 2 are the first and second weeks following that which represent future weeks. All 3 weeks were predicted based on the slope of the number of “data points” accumulated to date. The equation for the linear trend uses an *x*‐value that comes from the test fishery CPUE and the *y*‐value is the count of Chinook salmon that are predicted by the equation. The “cumulative” number of Chinook salmon are the sum of the three consecutive weeks predicted by the linear equation. The “observed” weeks are Chinook salmon counts at Bonneville dam for the same weeks predicted by the test fishery (three consecutive weeks 0–2) and the cumulative (sum across 3 weeks) was used to calculate the absolute error “abs error.”

## 4 DISCUSSION

This study demonstrated that the Spring Chinook Salmon Test Fishery (SCTF) on the Columbia River can provide means for accurate prediction of abundance and timing of the upriver adult Chinook salmon stock at a coarse level 2–4 weeks into the future. There were limitations on accuracy of prediction when discriminating upriver stock into finer categories, but results were robust for lower versus upriver stocks. The characteristics of the data from the SCTF predicted abundances broken down by VSI “lower” and “upriver” stocks, and further into adipose clipped and unclipped that determine hatchery origin and putatively natural origin. In addition, genetic analysis provided enhanced ability to break the coarse lower and upriver stocks into their genetic stock “GenStock” components, and for hatchery‐origin fish, stock‐specific resolution can be provided as fine a level as hatchery broodstock. However, fine‐scale resolution was specifically limited for natural origin upriver stocks that were rarely sampled in the test fishery.

### 4.1 How low can you go?

Sample sizes were constraining for accuracy in estimation of stock categories of low abundance. For stocks assessed in this study, the upriver stock generally had higher abundance than the lower stock, and within either stock, the AD fish had higher abundance than the AI fish. Therefore, the predictions for upriver fish that were hatchery origin had greatest potential for accuracy at the finest levels, that is, GenStock or even broodstock level. Our testing showed that composition of upriver hatchery origin stocks in the test fishery samples maintained accurate representation of the run at Bonneville dam down to the temporal scale of weeks and at the stock level of hatchery broodstock. Unfortunately, the natural‐origin stocks (a component of the AI fish) are a category of fish that include ESA listed stocks (e.g., upper Columbia River spring and Snake River spring/summer stocks), but the ability to break these stocks into ESA listed GenStock may be precluded by insufficient sample sizes in the test fishery. However, utility for estimating the proportion of natural‐origin fish as a whole may still be useful for fishery planning and is achievable with the SCTF data. For example, in years when the test fishery indicates that the natural‐origin fish are lower or higher than what the preseason forecast has estimated, these data could be used as supportive information for adjustments to scheduled harvest.

Importantly, the conversion of CPUE from the test fishery to adult Chinook salmon counted at Bonneville dam was not consistent across years and indicated that the model must be updated annually to provide accuracy necessary for predictive purposes. This CPUE conversion varied from 4317 to 7877 adult Chinook salmon at Bonneville dam in the most recent collection years (2018–2022) at the coarsest level of VSI‐defined upriver stock (i.e., “total” Chinook salmon combining AD and AI). Potential reasons for this variation in CPUE conversion across years may include differences in uniformity of the run of Chinook salmon across the river, differences in vulnerability of fish caught in the test fishery, or operational variation in timing and placement of drifts for the participating test fishery vessels. Regardless of the mechanism, a lack of consistency in CPUE conversion across years means that using a single conversion value (based on an average across linear trends from previous years) will not provide the most reliable prediction of adult Chinook salmon at Bonneville dam in a given year. This study also demonstrated that the conversion decreased at finer levels of stock discrimination (e.g., at the coarse level it was 6191 and 5897 for the VSI and GSI, respectively, but at the GenStock level, the average was 5187, and at the broodstock level it was 4428). Furthermore, the “lower” stock CPUE did not convert to a number of adult Chinook salmon counted at Willamette Falls that was in the same scale as Chinook salmon counted at Bonneville dam (e.g., the conversion at Willamette Falls for the coarse stock level averaged 2106 and 2318 for VSI and GSI, respectively; the 04_WILLAM GenStock average was 2900 Chinook salmon) and did not vary in a consistent way with year (i.e., the year in which this conversion value was maximum for Bonneville dam did not occur in the same year as the maximum conversion value for Willamette Falls). These patterns suggested that although the test fishery CPUE data can act as a proxy for abundance and was correlated with abundance at both Bonneville dam and Willamette Falls, there was no universal conversion that can be used to predict abundance in a given year and these relationships must be tuned to the data each year for precision.

### 4.2 Travel time varies within the spring season

The estimates of average travel time of 17.7 days (range 13–22 days for 2010–2015; Wargo Rub et al., 2019) have been shown to vary across years for Chinook salmon, but most dramatically within season and as little as 15–28 days difference has been estimated between early season versus later season fish within years 2011–2015. The more recent years of data (2017–2021) analyzed in this study corroborated this finding showing that average travel times could differ by as much as 28 days between groups of fish captured in the earliest weeks of the season compared to the latest weeks. A consistent trend occurred each year in which fish exhibited faster travel times as the season progressed. This trend helped to explain why the Bonneville dam counts of Chinook salmon in 2017 was lagged by a relatively long period of time (4 weeks) from the peak in CPUE of the test fishery as compared to the data from more recent years (consistently lagged 2 weeks). As a caveat, the 2017 dataset ended abruptly as CPUE was increasing and may not have captured the full peak of the run. However, alignment of the 2017 test fishery data with Bonneville dam counts was correctly lagged (4 weeks) based on how the CPUE conversion was in a similar range with the other years and information from travel rates from PIT tag data that year.

The estimates of travel time based on PIT tag data from our study and the previous study by Wargo Rub et al. (2019) were helpful in understanding why there is consistently a 2‐week lag between the test fishery CPUE and the weekly counts of spring Chinook at Bonneville dam for most years. The test fishery CPUE is essentially a pulse reading that occurs on a single day at the beginning of a statistical week and yet we determined that the week of fish counts that passed Bonneville dam between the 14th day and the 20th day after the test fishery day (i.e., the week of counts occurring 2 statistical weeks later), was the time lag that produced the best fit linear regression. This 2‐week time lag makes sense given that the average travel time has been reported as 17.7 days (Wargo Rub et al., 2019) and was estimated for the peak of the run to be 23.4 days, and so fish counted passing Bonneville dam between the 14th and 20th days after the test fishery day would represent the same pulse of fish.

One explanation for why Chinook salmon were entering the Columbia River earlier than average in 2017 and exhibiting longer travel times could also be due to higher than average river discharge that year (59% higher than the average discharge of 201 kcfs in spring period months over the years 2017–2022 in the dataset). The Columbia River (as measured by the gauge at Vancouver, WA; https://waterdata.usgs.gov/) recorded the highest discharge averaged across spring period months in 2017 (320 kcfs) compared to the average spring period discharge in other years in the dataset (range 180–274 kcfs). Although we found that a consistent use of a 2‐week time lag across most years in our dataset was sufficient to result in good fits for linear regressions, we acknowledge that a future model should take a more sophisticated approach to incorporate the information on the observed trend that travel rates accelerate through the season.

### 4.3 High concordance between VSI and GSI

The concordance of VSI and GSI was less than 100% on average across years (average of 83.3%), but close enough that VSI alone can afford similar levels of predictive ability for the strength of the upriver and lower stocks at coarse levels. This is important because VSI can be conducted in minutes and analysis of CPUE data in the test fishery can be used to generate predictions in the same day that the test fishery is executed. This fast turnaround time allows the SCTF data to be utilized in a time frame that nearly maximizes the period of time that the CPUE proxy of abundance is registered in advance of the run materializing at Bonneville dam or Willamette Falls to be enumerated there. In contrast, results from genetic analyses were processed rapidly with advanced genotyping practices but still required days rather than minutes to complete once tissues were received at the laboratory.

### 4.4 Benefits and logistical challenges for in‐season application that combines VSI and GSI

Benefits of an in‐season application of the SCTF data include providing an independent source of information to support results from existing methods used by the *U.S. v OR* Technical Advisory Committee (TAC) that provide preseason and in‐season forecasts of Spring Chinook. The potential advantage of the SCTF data over the current methods for preseason and in‐season forecasts is that it provides information on strength and timing of the upriver stock of spring Chinook salmon using a proxy of abundance (CPUE) being measured in the current year and with forecast strength at least 2 weeks in advance of the fish being enumerated at Bonneville dam. In contrast, the *U.S. v OR* TAC in‐season forecast relies on estimating the timing of the entire spring Chinook salmon run relative to the average timing of the past 5 years and then interpolates the run size according to cumulative counts of fish passing Bonneville dam to date. This method of in‐season forecasting can be sensitive to deviations in run timing of the spring Chinook salmon in the current year relative to the average run timing of the past 5 years. Therefore, the SCTF data would be a potential supplement to the *U.S. v OR* TAC preseason and in‐season forecasts, providing a means to understand the strength and timing of the run prior to it being observed at Bonneville dam. The test fishery also encounters lower river stocks that are rarely observed passing Bonneville dam and thus enables these stocks to be estimated with a combination of VSI and GSI.

Our guidance for how the test fishery VSI data could be used in‐season and possibly enhanced by GSI would be to implement the following steps: (1) calculate test fishery CPUE of VSI lower and upriver stocks each week for five statistical weeks (12–16); (2) utilize an average CPUE conversion (e.g., based on the average slope of linear regressions of upriver CPUE vs. Bonneville dam abundance for the recent years 2018–2022) to provide a preliminary weekly forecasts of abundance at Bonneville dam and Willamette Falls; (3) prior to the eighth test fishery opening (start of statistical week 19), use a linear regression on the first five data points to provide improved accuracy for predicted abundance at Bonneville dam for three future statistical weeks 19, 20, and 21; (4) genotype all test fishery samples from statistical weeks 12–19 before the end of statistical week 19 to provide stock‐specific abundance predictions at Bonneville dam for Chinook salmon that migrate above the dam approximately between statistical weeks 14–21.

Often the recreational fishery has a number of scheduled fisheries that occur prior to the preseason forecast being updated to an in‐season forecast by TAC. For example, in 2021, guidance from Oregon and Washington Fish and Wildlife commissions allowed 2234 upriver Spring Chinook (kept fish plus release mortalities) which ended up being 55% the total harvest of upriver Spring Chinook below Bonneville dam for the spring period that year (4088 total includes kept fish and release mortalities, 2022 Joint Staff Report). That year TAC updated the upriver run size in statistical week 21 on May 17 (87 k which was increased from 75,200 preseason forecast). In this example, information from the test fishery at the beginning of statistical week 19 could have been helpful timing for the point at which TAC made a decision on the estimate of the run. This example timing from 2021 shows how the test fishery data could be a useful supplement to other sources of data that TAC relies upon.

The challenge for application of these genetic analyses in a test fishery is whether their costs can be budgeted and whether they can be executed quickly enough to allow for advanced informing of fisheries. Advances in genotyping and sequencing technology (GT‐seq, Campbell et al., 2015) have allowed for cost efficiencies to budget for genetic analysis to conduct both GSI and PBT in the Columbia River Chinook salmon fisheries (Jensen et al., 2021) and allow for timely processing within 3 days of receipt of the samples. Therefore, if tissue samples from the test fishery could be sent soon after the eighth test fishery opening is conducted, it is possible to genotype the test fishery samples from statistical weeks 12 to 19 and have a relatively accurate prediction of stock‐specific abundance at Bonneville dam for Chinook salmon migrating above the dam through statistical week 21. Or if necessary, tissues from each test fishery opening could be genotyped before the end of that same week to provide a preliminary report on the composition of upriver stocks on a weekly basis that forecasts stock‐specific abundance 2 weeks in advance of their arrival at Bonneville dam. However, expedited turnaround times to process these relatively small groups of tissue samples decreases overall laboratory cost efficiencies that are gained by running large numbers of samples in high‐throughput capacities achievable with GT‐seq. Therefore, in‐season processing would have to be prioritized and balanced with existing workloads. One reason that expedited test fishery data genotyping could be given increased priority in the future is if 2‐week advanced stock‐specific prediction was found to be a useful means of forecasting ESA‐listed stocks (e.g., natural‐origin Snake River and upper Columbia River spring Chinook). Currently, the relatively low abundance of natural‐origin stocks encountered in the test fishery has precluded sufficient sample sizes to achieve the same level of accuracy for Bonneville dam abundance prediction that was demonstrated in this study at a coarser level (e.g., total upriver stock including both clipped and unclipped Chinook salmon). If larger sample sizes were obtained in the test fishery (e.g., by either increasing the fleet of test fishing boats or numbers of drifts per boat), this may provide a means to increase accuracy of predictions for stocks and groups of fish with low abundance, including ESA‐listed stocks.

## CONFLICT OF INTEREST STATEMENT

One of the co‐authors of this article helps manage contracts related to the Test Fishery but does not benefit financially or otherwise from the existence of this fishery.

## Supporting information


Appendix S1.

## Data Availability

GSI and PBT baselines that were analyzed in this study are available on FishGen as stated in the Methods. The individual genotypes and metadata associated with the mixture samples from the test fishery and Bonneville dam are available in dryad (DOI): doi: 10.5061/dryad.xwdbrv1md.
