# Supplementary material for: Visual and genetic stock identification of a test fishery to forecast Columbia River spring Chinook salmon stocks 2 weeks into the future
Source: Evol Appl. 2024 Mar 8;17(3):e13667. doi: 10.1111/eva.13667 (PMC10923652; doi:10.1111/eva.13667)
Supplement: Supplementary file 1 — Appendix S1. [file EVA-17-e13667-s001.docx]

Supplemental Material

Figures


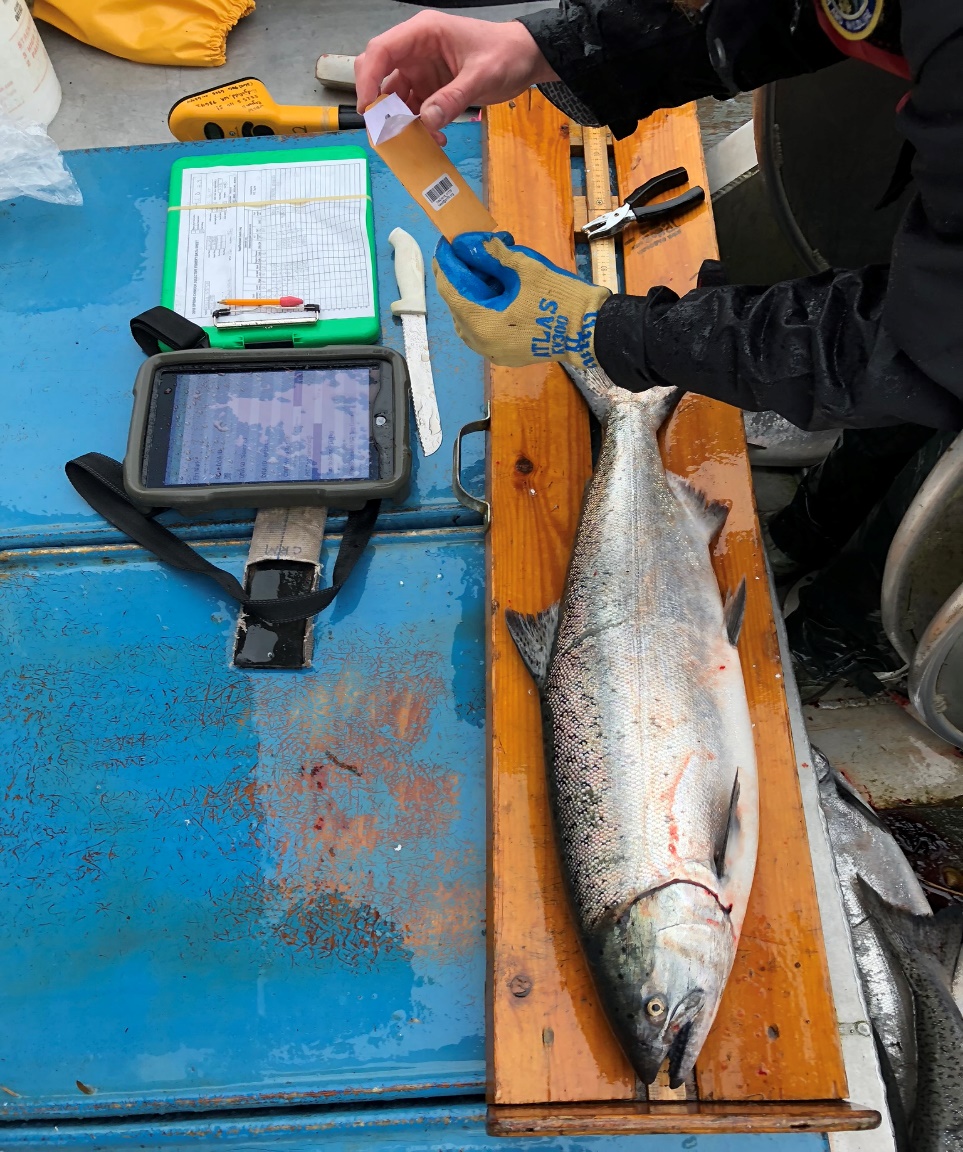


OtsZnlo_01776


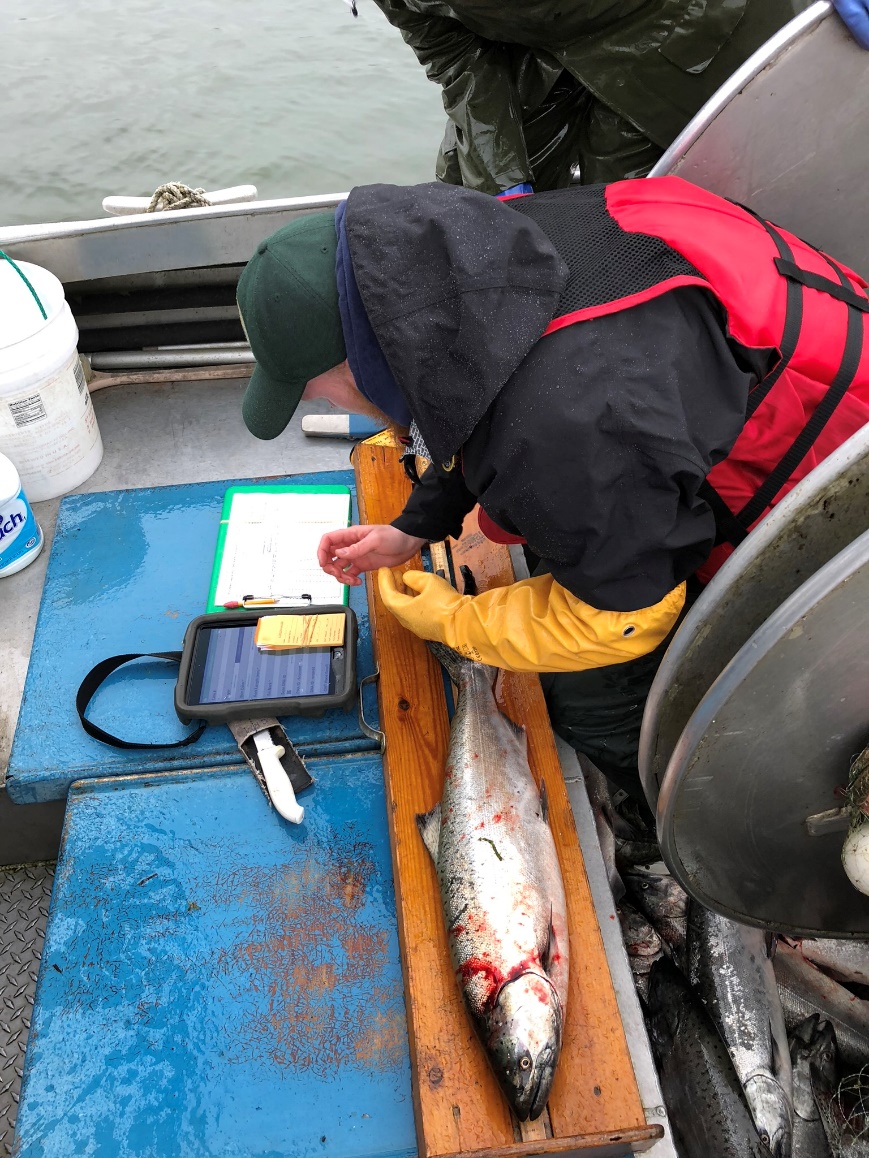


Nathan White WDFW

OtsZnlo_01782

Figure S1. Example of a visually stock identified (VSI) fish as upriver (left) and lower (right) Spring Chinook Salmon. The GSI confirmed these VSI calls and further identified the Genetic Stock as Hells Canyon (HELC) and the hatchery broodstock as Lookingglass Hatchery BY2014 (4-year-old) for the upriver fish. The Genetic Stock of the lower fish was Willamette (“04_WILLAM”) based on GSI results.

Figure S2. The linear regressions of the upriver CPUE versus Bonneville Dam adult-sized Chinook Salmon counts (lagged 2 weeks from the test fishery openings) based on VSI (left) and GSI (right) for years 2017 – 2022. Chinook Salmon are classified by adipose fin clips, i.e., clipped (AD), unclipped (AI), and total (AD + AI).

Figure S3. The linear regression of the upriver CPUE versus Bonneville dam adult-sized Chinook Salmon counts (lagged 4 weeks from the test fishery opening) based on VSI (top) and GSI (bottom) for collection year 2017. Chinook Salmon are classified by adipose fin clips, i.e., clipped (AD), unclipped (AI), and total (AD + AI).

Figure S4. The linear regression of the upriver CPUE versus Bonneville dam adult-sized Chinook Salmon counts (lagged 2 weeks from the test fishery opening) at the unit of GenStock (GSI reporting groups) for collection years 2017 – 2022. Each point represents a specific GenStock (letter codes listed in Table S3) on a specific statistical week (2-digit number “WK”). Estimates of CPUE and Bonneville Dam abundance are based on “total” Chinook (AD + AI).

Figure S5. The linear regression of the upriver CPUE versus Bonneville dam adult-sized Chinook Salmon counts (lagged 2 weeks from the test fishery opening) at the unit of hatchery clipped broodstock for collection years 2017 – 2022. Each point represents a specific hatchery clipped broodstock (3-digit “B” broodstock codes listed in Table S2) on a specific statistical week (2-digit number). Estimates of CPUE and Bonneville Dam abundance are based only on hatchery clipped Chinook Salmon (AD).

1. f) k)
2. g) l)
3. h) m)
4. i) n)
5. j) o)

Figure S6. Willamette Falls adult Chinook Salmon counts versus CPUE of lower stock in the Spring Chinook Salmon Test Fishery based on VSI (a – e) and GSI (f – j) and 04_WILLAM GenStock (k – o).

a) f) k)

b) g) l)

c) h) m)

d) i) n)

e) j) o)

Figure S7. Regressions of Willamette Falls adult Chinook Salmon counts (lagged 2 or 4 weeks, for recent years and year 2017, respectively) versus CPUE of lower stock in the Spring Chinook Salmon Test Fishery based on VSI (a – e), GSI (f – j), and 04_WILLAM GenStock (k – o).

Table S1. Stratification of Test Fishery Data

|  |  |  | VSI: Lower river | | | | | | VSI: Upriver | | | | | |  |
| --- | --- | --- | --- | --- | --- | --- | --- | --- | --- | --- | --- | --- | --- | --- | --- |
|  |  |  | AD | | | AI | | | AD | | | AI | | | Grand |
| Year | Week | Strata | GSI | PBT | Total | GSI | PBT | Total | GSI | PBT | Total | GSI | PBT | Total | Total |
| 2017 | 12 | 1 | 9 | 2 | 11 | 2 |  | 2 | 4 | 4 | 8 | 2 |  | 2 | 23 |
|  | 13 | 1 | 11 |  | 11 | 1 |  | 1 | 6 | 20 | 26 | 2 |  | 2 | 40 |
|  | 14 | 2 | 17 |  | 17 |  | 1 | 1 | 8 | 36 | 44 | 2 |  | 2 | 64 |
|  | 15 | 2 | 7 |  | 7 | 5 | 2 | 7 | 13 | 28 | 41 | 4 | 3 | 7 | 62 |
| 2017 Total |  |  | 44 | 2 | 46 | 8 | 3 | 11 | 31 | 88 | 119 | 10 | 3 | 13 | 189 |
| 2018 | 12 | 1 | 2 | 1 | 3 |  |  | 0 | 1 |  | 1 |  |  | 0 | 4 |
|  | 13 | 1 | 3 |  | 3 | 1 |  | 1 | 1 | 6 | 7 | 2 | 1 | 3 | 14 |
|  | 14 | 1 | 2 | 1 | 3 |  |  | 0 | 1 | 5 | 6 |  |  | 0 | 9 |
|  | 15 | 1 | 8 | 9 | 17 | 1 | 1 | 2 | 3 | 8 | 11 | 4 | 2 | 6 | 36 |
|  | 16 | 2 | 16 | 6 | 22 | 5 |  | 5 | 7 | 45 | 52 | 2 | 5 | 7 | 86 |
|  | 17 | 3 | 25 | 12 | 37 | 3 |  | 3 | 9 | 56 | 65 | 6 | 4 | 10 | 115 |
|  | 18 | 3 | 16 |  | 16 |  |  | 0 | 7 | 36 | 43 |  |  | 0 | 59 |
| 2018 Total |  |  | 72 | 29 | 101 | 10 | 1 | 11 | 29 | 156 | 185 | 14 | 12 | 26 | 323 |
| 2019 | 12 | 1 | 1 |  | 1 | 2 |  | 2 |  |  | 0 | 1 |  | 1 | 4 |
|  | 13 | 1 | 4 |  | 4 | 3 |  | 3 | 1 | 3 | 4 |  |  | 0 | 11 |
|  | 14 | 1 | 2 |  | 2 | 2 |  | 2 |  | 5 | 5 |  |  | 0 | 9 |
|  | 15 | 1 | 1 | 1 | 2 | 3 |  | 3 |  |  | 0 |  |  | 0 | 5 |
|  | 16 | 1 | 15 | 10 | 25 | 9 | 1 | 10 | 5 | 21 | 26 | 6 | 2 | 8 | 69 |
|  | 17 | 2 | 4 | 4 | 8 | 1 |  | 1 | 1 | 21 | 22 | 1 |  | 1 | 32 |
|  | 18 | 2 | 20 | 5 | 25 | 2 |  | 2 | 8 | 11 | 19 | 2 | 1 | 3 | 49 |
|  | 19 | 2 | 6 | 1 | 7 | 1 |  | 1 |  | 5 | 5 | 5 |  | 5 | 18 |
| 2019 Total |  |  | 53 | 21 | 74 | 23 | 1 | 24 | 15 | 66 | 81 | 15 | 3 | 18 | 197 |
| 2021 | 12 | 1 |  |  | 0 |  |  | 0 |  | 1 | 1 |  |  | 0 | 1 |
|  | 13 | 1 |  |  | 0 |  |  | 0 | 1 | 4 | 5 | 1 |  | 1 | 6 |
|  | 14 | 1 | 3 |  | 3 |  |  | 0 | 1 | 2 | 3 |  |  | 0 | 6 |
|  | 15 | 1 | 4 | 5 | 9 |  |  | 0 |  | 3 | 3 |  |  | 0 | 12 |
|  | 16 | 1 | 4 | 2 | 6 |  | 1 | 1 | 3 | 16 | 19 | 1 | 3 | 4 | 30 |
|  | 17 | 1 | 5 | 2 | 7 |  | 1 | 1 |  | 12 | 12 | 4 | 2 | 6 | 26 |
|  | 18 | 2 | 7 | 5 | 12 | 5 |  | 5 | 4 | 10 | 14 |  | 3 | 3 | 34 |
|  | 19 | 2 | 3 |  | 3 |  |  | 0 | 1 | 4 | 5 | 3 |  | 3 | 11 |
|  | 20 | 2 | 3 | 1 | 4 | 1 | 1 | 2 |  | 2 | 2 | 1 |  | 1 | 9 |
| 2021 Total |  |  | 29 | 15 | 44 | 6 | 3 | 9 | 10 | 54 | 64 | 10 | 8 | 18 | 135 |
| 2022 | 12 | 1 | 1 | 1 | 2 |  |  | 0 | 2 | 4 | 6 | 1 |  | 1 | 9 |
|  | 13 | 1 | 1 | 3 | 4 |  |  | 0 | 1 | 2 | 3 |  |  | 0 | 7 |
|  | 14 | 1 | 3 | 1 | 4 |  |  | 0 | 2 | 9 | 11 | 1 |  | 1 | 16 |
|  | 15 | 1 | 1 | 7 | 8 | 1 |  | 1 | 2 | 12 | 14 | 3 |  | 3 | 26 |
|  | 16 | 1 | 2 | 2 | 4 |  |  | 0 | 4 | 21 | 25 | 4 | 3 | 7 | 36 |
|  | 17 | 1 | 2 | 4 | 6 | 3 |  | 3 | 5 | 33 | 38 | 9 | 5 | 14 | 61 |
|  | 18 | 2 | 6 | 2 | 8 |  |  | 0 | 6 | 24 | 30 | 3 | 1 | 4 | 42 |
|  | 19 | 2 | 12 | 2 | 14 | 2 |  | 2 | 4 | 24 | 28 | 4 | 4 | 8 | 52 |
|  | 20 | 2 | 11 | 4 | 15 | 2 |  | 2 | 4 | 24 | 28 | 4 | 3 | 7 | 52 |
|  | 21 | 3 | 10 | 7 | 17 | 2 |  | 2 |  | 7 | 7 | 7 | 1 | 8 | 34 |
|  | 22 | 3 | 1 |  | 1 |  |  | 0 |  | 3 | 3 |  |  | 0 | 4 |
|  | 23 | 3 | 5 | 4 | 9 |  |  | 0 |  | 4 | 4 | 1 |  | 1 | 14 |
|  | 24 | 3 | 4 | 3 | 7 |  |  | 0 |  |  | 0 |  |  | 0 | 7 |
| 2022 Total |  |  | 59 | 40 | 99 | 10 |  | 10 | 30 | 167 | 197 | 37 | 17 | 54 | 360 |
| Grand Total |  |  | 257 | 107 | 364 | 57 | 8 | 65 | 115 | 531 | 646 | 86 | 43 | 129 | 1204 |

Note: The statistical “week” is listed for number of samples analyzed in each year that the Test Fishery was conducted. Weeks were pooled into “strata” to attain a target sample size per stratum. For each set of adipose-clipped (AD) and adipose-intact (AI) group of fish we indicate the number of samples that were assigned using “PBT” and the number of samples that had “GSI” only (PBT-unassigned). Visual stock identification “VSI” from the Test Fishery observation data was used to first classify the sample into “lower” and “upriver” stocks.

Table S2. PBT baseline hatchery broodstocks classified into expected GenStock and associated with their tag rates.

| Broodstock | Hatchery | Stock | Basin | Run | Lineage | GenStock_exp | BY | Rate | N | Code |
| --- | --- | --- | --- | --- | --- | --- | --- | --- | --- | --- |
| OtsCLWH_seg_sp08 | Clearwater Fish Hatchery | Clearwater River | Snake | 01Spring | Interior stream type | 12_HELLSC | 2008 | 0.9216 | 988 | 001 |
| OtsPOWP_seg_sp08 | Clearwater Fish Hatchery | Lochsa River (Powell Facility) | Snake | 01Spring | Interior stream type | 12_HELLSC | 2008 | 1.0000 | 864 | 002 |
| OtsDWOR_seg_sp08 | Dworshak National Fish Hatchery | Clearwater River | Snake | 01Spring | Interior stream type | 12_HELLSC | 2008 | 1.0000 | 1203 | 003 |
| OtsKLIC_seg_sp08 | Klickitat Hatchery | Klickitat River | Columbia | 01Spring | Interior stream type | 06_KLICKR | 2008 | 1.0000 | 232 | 004 |
| OtsIMNW_seg_ss08 | Lookingglass Fish Hatchery | Imnaha River | Snake | 02Spring/Summer | Interior stream type | 12_HELLSC | 2008 | 0.9518 | 246 | 005 |
| OtsLOOK_seg_sp08 | Lookingglass Fish Hatchery | Lookingglass/Catherine Creek/Grande Ronde/Lostine River | Snake | 01Spring | Interior stream type | 12_HELLSC | 2008 | 0.9968 | 624 | 006 |
| OtsLYON_seg_sp08 | Lyons Ferry Fish Hatchery | Tucannon River | Snake | 01Spring | Interior stream type | 11_TUCANO | 2008 | 1.0000 | 119 | 007 |
| OtsJHNW_int_ss08 | McCall Fish Hatchery | Johnson Creek | Snake | 02Spring/Summer | Interior stream type | 13_SFSALM | 2008 | 0.9802 | 57 | 008 |
| OtsMCCA_seg_ss08 | McCall Fish Hatchery | South Fork Salmon | Snake | 02Spring/Summer | Interior stream type | 13_SFSALM | 2008 | 1.0000 | 1881 | 009 |
| OtsNPFH_seg_sp08 | Nez Perce | Newsome Creek and Yoosa/Camp Creek | Snake | 01Spring | Interior stream type | 12_HELLSC | 2008 | 1.0000 | 191 | 010 |
| OtsPAHH_seg_ss08 | Pahsimeroi Fish Hatchery | Salmon River | Snake | 02Spring/Summer | Interior stream type | 16_UPSALM | 2008 | 1.0000 | 706 | 011 |
| OtsRAPH_seg_sp08 | Rapid River Fish Hatchery | Rapid River | Snake | 01Spring | Interior stream type | 12_HELLSC | 2008 | 0.9993 | 2796 | 012 |
| OtsSAWT_seg_ss08 | Sawtooth Fish Hatchery | Salmon River | Snake | 02Spring/Summer | Interior stream type | 16_UPSALM | 2008 | 0.9899 | 1183 | 013 |
| OtsCLWH_seg_sp09 | Clearwater Fish Hatchery | Clearwater River | Snake | 01Spring | Interior stream type | 12_HELLSC | 2009 | 0.9970 | 668 | 014 |
| OtsPOWP_seg_sp09 | Clearwater Fish Hatchery | Lochsa River (Powell Facility) | Snake | 01Spring | Interior stream type | 12_HELLSC | 2009 | 0.9927 | 545 | 015 |
| OtsDWOR_seg_sp09 | Dworshak National Fish Hatchery | Clearwater River | Snake | 01Spring | Interior stream type | 12_HELLSC | 2009 | 1.0000 | 1176 | 016 |
| OtsKLIC_seg_sp09 | Klickitat Hatchery | Klickitat River | Columbia | 01Spring | Interior stream type | 06_KLICKR | 2009 | 1.0000 | 762 | 017 |
| OtsIMNW_seg_ss09 | Lookingglass Fish Hatchery | Imnaha River | Snake | 02Spring/Summer | Interior stream type | 12_HELLSC | 2009 | 1.0000 | 223 | 018 |
| OtsLOOK_seg_sp09 | Lookingglass Fish Hatchery | Lookingglass/Catherine Creek/Grande Ronde/Lostine River | Snake | 01Spring | Interior stream type | 12_HELLSC | 2009 | 0.9886 | 687 | 019 |
| OtsLYON_seg_sp09 | Lyons Ferry Fish Hatchery | Tucannon River | Snake | 01Spring | Interior stream type | 11_TUCANO | 2009 | 0.9326 | 169 | 020 |
| OtsJHNW_int_ss09 | McCall Fish Hatchery | Johnson Creek | Snake | 02Spring/Summer | Interior stream type | 13_SFSALM | 2009 | 0.6075 | 39 | 021 |
| OtsMCCA_seg_ss09 | McCall Fish Hatchery | South Fork Salmon | Snake | 02Spring/Summer | Interior stream type | 13_SFSALM | 2009 | 0.9593 | 927 | 022 |
| OtsNPFH_seg_sp09 | Nez Perce | Newsome Creek and Yoosa/Camp Creek | Snake | 01Spring | Interior stream type | 12_HELLSC | 2009 | 1.0000 | 420 | 023 |
| OtsPAHH_seg_ss09 | Pahsimeroi Fish Hatchery | Salmon River | Snake | 02Spring/Summer | Interior stream type | 16_UPSALM | 2009 | 0.9747 | 620 | 024 |
| OtsRAPH_seg_sp09 | Rapid River Fish Hatchery | Rapid River | Snake | 01Spring | Interior stream type | 12_HELLSC | 2009 | 0.9990 | 2017 | 025 |
| OtsSAWT_seg_ss09 | Sawtooth Fish Hatchery | Salmon River | Snake | 02Spring/Summer | Interior stream type | 16_UPSALM | 2009 | 0.8236 | 893 | 026 |
| OtsCLWH_seg_sp10 | Clearwater Fish Hatchery | Clearwater River | Snake | 01Spring | Interior stream type | 12_HELLSC | 2010 | 1.0000 | 741 | 027 |
| OtsPOWP_seg_sp10 | Clearwater Fish Hatchery | Lochsa River (Powell Facility) | Snake | 01Spring | Interior stream type | 12_HELLSC | 2010 | 1.0000 | 413 | 028 |
| OtsDWOR_seg_sp10 | Dworshak National Fish Hatchery | Clearwater River | Snake | 01Spring | Interior stream type | 12_HELLSC | 2010 | 1.0000 | 1227 | 029 |
| OtsKLIC_seg_sp10 | Klickitat Hatchery | Klickitat River | Columbia | 01Spring | Interior stream type | 06_KLICKR | 2010 | 1.0000 | 673 | 030 |
| OtsIMNW_seg_ss10 | Lookingglass Fish Hatchery | Imnaha River | Snake | 02Spring/Summer | Interior stream type | 12_HELLSC | 2010 | 1.0000 | 242 | 031 |
| OtsLOOK_seg_sp10 | Lookingglass Fish Hatchery | Lookingglass/Catherine Creek/Grande Ronde/Lostine River | Snake | 01Spring | Interior stream type | 12_HELLSC | 2010 | 0.9880 | 780 | 032 |
| OtsLYON_seg_sp10 | Lyons Ferry Fish Hatchery | Tucannon River | Snake | 01Spring | Interior stream type | 11_TUCANO | 2010 | 0.9633 | 159 | 033 |
| OtsJHNW_int_ss10 | McCall Fish Hatchery | Johnson Creek | Snake | 02Spring/Summer | Interior stream type | 13_SFSALM | 2010 | 1.0000 | 68 | 034 |
| OtsMCCA_seg_ss10 | McCall Fish Hatchery | South Fork Salmon | Snake | 02Spring/Summer | Interior stream type | 13_SFSALM | 2010 | 1.0000 | 1004 | 035 |
| OtsNPFH_seg_sp10 | Nez Perce | Newsome Creek and Yoosa/Camp Creek | Snake | 01Spring | Interior stream type | 12_HELLSC | 2010 | 0.9838 | 487 | 036 |
| OtsPAHH_seg_ss10 | Pahsimeroi Fish Hatchery | Salmon River | Snake | 02Spring/Summer | Interior stream type | 16_UPSALM | 2010 | 1.0000 | 556 | 037 |
| OtsRAPH_seg_sp10 | Rapid River Fish Hatchery | Rapid River | Snake | 01Spring | Interior stream type | 12_HELLSC | 2010 | 1.0000 | 2291 | 038 |
| OtsSAWT_seg_ss10 | Sawtooth Fish Hatchery | Salmon River | Snake | 02Spring/Summer | Interior stream type | 16_UPSALM | 2010 | 0.8829 | 638 | 039 |
| OtsCLWH_seg_sp11 | Clearwater Fish Hatchery | Clearwater River | Snake | 01Spring | Interior stream type | 12_HELLSC | 2011 | 1.0000 | 566 | 040 |
| OtsPOWP_seg_sp11 | Clearwater Fish Hatchery | Lochsa River (Powell Facility) | Snake | 01Spring | Interior stream type | 12_HELLSC | 2011 | 1.0000 | 846 | 041 |
| OtsDWOR_seg_sp11 | Dworshak National Fish Hatchery | Clearwater River | Snake | 01Spring | Interior stream type | 12_HELLSC | 2011 | 1.0000 | 1411 | 042 |
| OtsKLIC_seg_sp11 | Klickitat Hatchery | Klickitat River | Columbia | 01Spring | Interior stream type | 06_KLICKR | 2011 | 1.0000 | 521 | 043 |
| OtsIMNW_seg_ss11 | Lookingglass Fish Hatchery | Imnaha River | Snake | 02Spring/Summer | Interior stream type | 12_HELLSC | 2011 | 1.0000 | 247 | 044 |
| OtsLOOK_seg_sp11 | Lookingglass Fish Hatchery | Lookingglass/Catherine Creek/Grande Ronde/Lostine River | Snake | 01Spring | Interior stream type | 12_HELLSC | 2011 | 0.9679 | 653 | 045 |
| OtsLYON_seg_fa11 | Lyons Ferry Fish Hatchery | Mixed origins (Snake River and Upper Columbia River) | Snake | 04Fall | Interior ocean type | 19_SRFALL | 2011 | 0.9670 | 1728 | 046 |
| OtsLYON_seg_sp11 | Lyons Ferry Fish Hatchery | Tucannon River | Snake | 01Spring | Interior stream type | 11_TUCANO | 2011 | 0.9760 | 164 | 047 |
| OtsJHNW_int_ss11 | McCall Fish Hatchery | Johnson Creek | Snake | 02Spring/Summer | Interior stream type | 13_SFSALM | 2011 | 1.0000 | 63 | 048 |
| OtsMCCA_seg_ss11 | McCall Fish Hatchery | South Fork Salmon | Snake | 02Spring/Summer | Interior stream type | 13_SFSALM | 2011 | 1.0000 | 919 | 049 |
| OtsNPFH_seg_fa11 | Nez Perce Tribal Fish Hatchery | Mixed origins (Snake River and Clearwater River) | Snake | 04Fall | Interior ocean type | 19_SRFALL | 2011 | 0.8888 | 640 | 050 |
| OtsNPFH_seg_sp11 | Nez Perce | Newsome Creek and Yoosa/Camp Creek | Snake | 01Spring | Interior stream type | 12_HELLSC | 2011 | 0.9834 | 356 | 051 |
| OtsPAHH_seg_ss11 | Pahsimeroi Fish Hatchery | Salmon River | Snake | 02Spring/Summer | Interior stream type | 16_UPSALM | 2011 | 1.0000 | 651 | 052 |
| OtsRAPH_seg_sp11 | Rapid River Fish Hatchery | Rapid River | Snake | 01Spring | Interior stream type | 12_HELLSC | 2011 | 0.9817 | 1878 | 053 |
| OtsSAWT_seg_ss11 | Sawtooth Fish Hatchery | Salmon River | Snake | 02Spring/Summer | Interior stream type | 16_UPSALM | 2011 | 0.9633 | 318 | 054 |
| OtsCARS_seg_sp12 | Carson National Fish Hatchery | Mixed origins (Snake and Mid-/Upper-Columbia Rivers) | Columbia | 01Spring | Interior stream type | 20_BONPOOLSP | 2012 | 0.8960 | 904 | 055 |
| OtsCLWH_seg_sp12 | Clearwater Fish Hatchery | Clearwater River | Snake | 01Spring | Interior stream type | 12_HELLSC | 2012 | 0.6828 | 606 | 056 |
| OtsPOWP_seg_sp12 | Clearwater Fish Hatchery | Lochsa River (Powell Facility) | Snake | 01Spring | Interior stream type | 12_HELLSC | 2012 | 1.0000 | 642 | 057 |
| OtsDWOR_seg_sp12 | Dworshak National Fish Hatchery | Clearwater River | Snake | 01Spring | Interior stream type | 12_HELLSC | 2012 | 1.0000 | 2086 | 058 |
| OtsEAST_seg_su12 | Eastbank Fish Hatchery | Chelan Falls, Methow River, Okanogan River, Wenatchee River | Columbia | 03Summer | Interior ocean type | 18_UCOLSF | 2012 | 1.0000 | 741 | 059 |
| OtsKLIC_seg_sp12 | Klickitat Hatchery | Klickitat River | Columbia | 01Spring | Interior stream type | 06_KLICKR | 2012 | 1.0000 | 248 | 060 |
| OtsIMNW_seg_ss12 | Lookingglass Fish Hatchery | Imnaha River | Snake | 02Spring/Summer | Interior stream type | 12_HELLSC | 2012 | 1.0000 | 242 | 061 |
| OtsLOOK_seg_sp12 | Lookingglass Fish Hatchery | Lookingglass/Catherine Creek/Grande Ronde/Lostine River | Snake | 01Spring | Interior stream type | 12_HELLSC | 2012 | 0.9685 | 502 | 062 |
| OtsLYON_seg_fa12 | Lyons Ferry Fish Hatchery | Mixed origins (Snake River and Upper Columbia River) | Snake | 04Fall | Interior ocean type | 19_SRFALL | 2012 | 0.9852 | 1729 | 063 |
| OtsLYON_seg_sp12 | Lyons Ferry Fish Hatchery | Tucannon River | Snake | 01Spring | Interior stream type | 11_TUCANO | 2012 | 0.9524 | 162 | 064 |
| OtsJHNW_int_ss12 | McCall Fish Hatchery | Johnson Creek | Snake | 02Spring/Summer | Interior stream type | 13_SFSALM | 2012 | 0.9025 | 57 | 065 |
| OtsMCCA_seg_ss12 | McCall Fish Hatchery | South Fork Salmon | Snake | 02Spring/Summer | Interior stream type | 13_SFSALM | 2012 | 0.9768 | 1018 | 066 |
| OtsMETH_seg_sp12 | Methow Fish Hatchery | Upper Methow and Twisp Rivers | Columbia | 01Spring | Interior stream type | 10_UCOLSP | 2012 | 0.9760 | 164 | 067 |
| OtsNPFH_seg_fa12 | Nez Perce Tribal Fish Hatchery | Mixed origins (Snake River and Clearwater River) | Snake | 04Fall | Interior ocean type | 19_SRFALL | 2012 | 0.9751 | 846 | 068 |
| OtsNPFH_seg_sp12 | Nez Perce | Newsome Creek and Yoosa/Camp Creek | Snake | 01Spring | Interior stream type | 12_HELLSC | 2012 | 0.9585 | 367 | 069 |
| OtsPAHH_seg_ss12 | Pahsimeroi Fish Hatchery | Salmon River | Snake | 02Spring/Summer | Interior stream type | 16_UPSALM | 2012 | 0.9903 | 402 | 070 |
| OtsPARK_seg_sp12 | Parkdale Fish Facility | Hood River | Columbia | 01Spring | Lower Columbia | 02_WCASSP | 2012 | 0.8738 | 129 | 071 |
| OtsPRIE_seg_fa12 | Priest Rapids Hatchery | Mixed origins (Mid-/Upper Columbia Rivers) | Columbia | 04Fall | Interior ocean type | 18_UCOLSF | 2012 | 1.0000 | 3894 | 072 |
| OtsRAPH_seg_sp12 | Rapid River Fish Hatchery | Rapid River | Snake | 01Spring | Interior stream type | 12_HELLSC | 2012 | 1.0000 | 1306 | 073 |
| OtsRBFH_seg_sp12 | Round Butte Fish Hatchery | Deschutes River | Columbia | 01Spring | Interior stream type | 07_DESCSP | 2012 | 0.9209 | 666 | 074 |
| OtsSAWT_seg_ss12 | Sawtooth Fish Hatchery | Salmon River | Snake | 02Spring/Summer | Interior stream type | 16_UPSALM | 2012 | 0.9980 | 965 | 075 |
| OtsUMAT_seg_fa12 | Umatilla Fish Hatchery |  | Columbia | 04Fall | Interior ocean type | 23_UMATILLAFA | 2012 | 1.0000 | 735 | 076 |
| OtsUMAT_seg_sp12 | Umatilla Fish Hatchery |  | Columbia | 01Spring | Interior stream type | 21_UMATILLASP | 2012 | 1.0000 | 391 | 077 |
| OtsWSNF_seg_sp12 | Warm Springs National Fish Hatchery |  | Columbia | 01Spring | Interior stream type | 07_DESCSP | 2012 | 0.5985 | 434 | 078 |
| OtsWELL_seg_su12 | Wells Fish Hatchery |  | Columbia | 03Summer | Interior ocean type | 18_UCOLSF | 2012 | 1.0000 | 753 | 079 |
| OtsPROS_seg_fa12 | Yakima Nation Prosser Hatchery |  | Columbia | 04Fall | Interior ocean type | 18_UCOLSF | 2012 | 1.0000 | 189 | 080 |
| OtsYRRD_int_sp12 | Yakima River Roza Dam | Integrated | Columbia | 01Spring | Interior stream type | 09_YAKIMA | 2012 | 0.9752 | 316 | 081 |
| OtsYRRD_seg_sp12 | Yakima River Roza Dam | Segregated | Columbia | 01Spring | Interior stream type | 09_YAKIMA | 2012 | 1.0000 | 344 | 082 |
| OtsCARS_seg_sp13 | Carson National Fish Hatchery | Mixed origins (Snake and Mid-/Upper-Columbia Rivers) | Columbia | 01Spring | Interior stream type | 20_BONPOOLSP | 2013 | 0.8215 | 910 | 083 |
| OtsCHJO_int_su13 | Chief Joseph Hatchery | Integrated | Columbia | 03Summer | Interior ocean type | 18_UCOLSF | 2013 | 0.6969 | 288 | 084 |
| OtsCHJO_seg_su13 | Chief Joseph Hatchery | Segregated | Columbia | 03Summer | Interior ocean type | 18_UCOLSF | 2013 | 0.8872 | 308 | 085 |
| OtsCLWH_seg_sp13 | Clearwater Fish Hatchery | Clearwater River | Snake | 01Spring | Interior stream type | 12_HELLSC | 2013 | 0.9783 | 805 | 086 |
| OtsPOWP_seg_sp13 | Clearwater Fish Hatchery | Lochsa River (Powell Facility) | Snake | 01Spring | Interior stream type | 12_HELLSC | 2013 | 1.0000 | 219 | 087 |
| OtsDWOR_seg_sp13 | Dworshak National Fish Hatchery | Clearwater River | Snake | 01Spring | Interior stream type | 12_HELLSC | 2013 | 0.9599 | 1811 | 088 |
| OtsEAST_seg_su13 | Eastbank Fish Hatchery | Chelan Falls, Methow River, Okanogan River, Wenatchee River | Columbia | 03Summer | Interior ocean type | 18_UCOLSF | 2013 | 1.0000 | 312 | 089 |
| OtsEAST_seg_sp13 | Eastbank Fish Hatchery | Chelan Falls, Methow River, Okanogan River, Wenatchee River | Columbia | 01Spring | Interior stream type | 10_UCOLSP | 2013 | 1.0000 | 172 | 090 |
| OtsENFH_seg_su13 | Entiat National Fish Hatchery | Entiat | Columbia | 03Summer | Interior ocean type | 18_UCOLSF | 2013 | 0.8964 | 267 | 091 |
| OtsKLIC_seg_sp13 | Klickitat Hatchery | Klickitat River | Columbia | 01Spring | Interior stream type | 06_KLICKR | 2013 | 1.0000 | 371 | 092 |
| OtsLNFH_seg_sp13 | Leavenworth National Fish Hatchery | Mixed origins (Upper-Columbia Rivers) | Columbia | 01Spring | Interior stream type | 10_UCOLSP | 2013 | 1.0000 | 729 | 093 |
| OtsLWSN_seg_sp13 | Little White Salmon National Fish Hatchery | Little White Salmon River | Columbia | 01Spring | Interior stream type | 20_BONPOOLSP | 2013 | 1.0000 | 622 | 094 |
| OtsLWSN_seg_fa13 | Little White Salmon National Fish Hatchery | Little White Salmon River | Columbia | 04Fall | Interior ocean type | 22_BONPOOLFA | 2013 | 1.0000 | 4979 | 095 |
| OtsIMNW_seg_ss13 | Lookingglass Fish Hatchery | Imnaha River | Snake | 02Spring/Summer | Interior stream type | 12_HELLSC | 2013 | 1.0000 | 188 | 096 |
| OtsLOOK_cap_sp13 | Lookingglass Fish Hatchery | Lookingglass Creek | Snake | 02Spring/Summer | Interior stream type | 12_HELLSC | 2013 | 0.9773 | 248 | 097 |
| OtsLOOK_seg_sp13 | Lookingglass Fish Hatchery | Lookingglass/Catherine Creek/Grande Ronde/Lostine River | Snake | 01Spring | Interior stream type | 12_HELLSC | 2013 | 0.9746 | 430 | 098 |
| OtsLYON_seg_fa13 | Lyons Ferry Fish Hatchery | Mixed origins (Snake River and Upper Columbia River) | Snake | 04Fall | Interior ocean type | 19_SRFALL | 2013 | 0.9240 | 1944 | 099 |
| OtsLYON_seg_sp13 | Lyons Ferry Fish Hatchery | Tucannon River | Snake | 01Spring | Interior stream type | 11_TUCANO | 2013 | 1.0000 | 149 | 100 |
| OtsJHNW_int_ss13 | McCall Fish Hatchery | Johnson Creek | Snake | 02Spring/Summer | Interior stream type | 13_SFSALM | 2013 | 0.9070 | 73 | 101 |
| OtsMCCA_seg_ss13 | McCall Fish Hatchery | South Fork Salmon | Snake | 02Spring/Summer | Interior stream type | 13_SFSALM | 2013 | 0.9841 | 1066 | 102 |
| OtsMETH_seg_sp13 | Methow Fish Hatchery | Upper Methow and Twisp Rivers | Columbia | 01Spring | Interior stream type | 10_UCOLSP | 2013 | 0.9415 | 98 | 103 |
| OtsNPFH_seg_fa13 | Nez Perce Tribal Fish Hatchery | Mixed origins (Snake River and Clearwater River) | Snake | 04Fall | Interior ocean type | 19_SRFALL | 2013 | 0.9910 | 865 | 104 |
| OtsNPFH_seg_sp13 | Nez Perce | Newsome Creek and Yoosa/Camp Creek | Snake | 01Spring | Interior stream type | 12_HELLSC | 2013 | 1.0000 | 391 | 105 |
| OtsPAHH_seg_ss13 | Pahsimeroi Fish Hatchery | Salmon River | Snake | 02Spring/Summer | Interior stream type | 16_UPSALM | 2013 | 0.9955 | 446 | 106 |
| OtsPARK_seg_sp13 | Parkdale Fish Facility | Hood River | Columbia | 01Spring | Lower Columbia | 02_WCASSP | 2013 | 0.6849 | 48 | 107 |
| OtsPRIE_seg_fa13 | Priest Rapids Hatchery | Mixed origins (Mid-/Upper Columbia Rivers) | Columbia | 04Fall | Interior ocean type | 18_UCOLSF | 2013 | 1.0000 | 4798 | 108 |
| OtsRAPH_seg_sp13 | Rapid River Fish Hatchery | Rapid River | Snake | 01Spring | Interior stream type | 12_HELLSC | 2013 | 0.9861 | 1864 | 109 |
| OtsRBFH_seg_sp13 | Round Butte Fish Hatchery | Deschutes River | Columbia | 01Spring | Interior stream type | 07_DESCSP | 2013 | 1.0000 | 412 | 110 |
| OtsSAWT_seg_ss13 | Sawtooth Fish Hatchery | Salmon River | Snake | 02Spring/Summer | Interior stream type | 16_UPSALM | 2013 | 0.9958 | 966 | 111 |
| OtsUMAT_seg_fa13 | Umatilla Fish Hatchery |  | Columbia | 04Fall | Interior ocean type | 23_UMATILLAFA | 2013 | 1.0000 | 800 | 112 |
| OtsUMAT_seg_sp13 | Umatilla Fish Hatchery |  | Columbia | 01Spring | Interior stream type | 21_UMATILLASP | 2013 | 1.0000 | 441 | 113 |
| OtsWSNF_seg_sp13 | Warm Springs National Fish Hatchery |  | Columbia | 01Spring | Interior stream type | 07_DESCSP | 2013 | 0.7351 | 481 | 114 |
| OtsWELL_seg_su13 | Wells Fish Hatchery |  | Columbia | 03Summer | Interior ocean type | 18_UCOLSF | 2013 | 1.0000 | 681 | 115 |
| OtsWINT_seg_sp13 | Winthrop National Fish Hatchery |  | Columbia | 01Spring | Interior stream type | 10_UCOLSP | 2013 | 0.7901 | 392 | 116 |
| OtsPROS_seg_fa13 | Yakima Nation Prosser Hatchery |  | Columbia | 04Fall | Interior ocean type | 18_UCOLSF | 2013 | 1.0000 | 279 | 117 |
| OtsYRRD_int_sp13 | Yakima River Roza Dam | Integrated | Columbia | 01Spring | Interior stream type | 09_YAKIMA | 2013 | 0.5860 | 271 | 118 |
| OtsYRRD_seg_sp13 | Yakima River Roza Dam | Segregated | Columbia | 01Spring | Interior stream type | 09_YAKIMA | 2013 | 0.3863 | 179 | 119 |
| OtsCARS_seg_sp14 | Carson National Fish Hatchery | Mixed origins (Snake and Mid-/Upper-Columbia Rivers) | Columbia | 01Spring | Interior stream type | 20_BONPOOLSP | 2014 | 0.8874 | 942 | 120 |
| OtsCHJO_int_su14 | Chief Joseph Hatchery | Integrated | Columbia | 03Summer | Interior ocean type | 18_UCOLSF | 2014 | 0.8920 | 476 | 121 |
| OtsCHJO_seg_sp14 | Chief Joseph Hatchery | Spring | Columbia | 01Spring | Interior stream type | 10_UCOLSP | 2014 | 0.8936 | 121 | 122 |
| OtsCHJO_seg_su14 | Chief Joseph Hatchery | Segregated | Columbia | 03Summer | Interior ocean type | 18_UCOLSF | 2014 | 0.4414 | 295 | 123 |
| OtsCLWH_seg_sp14 | Clearwater Fish Hatchery | Clearwater River | Snake | 01Spring | Interior stream type | 12_HELLSC | 2014 | 0.9710 | 1207 | 124 |
| OtsPOWP_seg_sp14 | Clearwater Fish Hatchery | Lochsa River (Powell Facility) | Snake | 01Spring | Interior stream type | 12_HELLSC | 2014 | 0.9905 | 618 | 125 |
| OtsDWOR_seg_sp14 | Dworshak National Fish Hatchery | Clearwater River | Snake | 01Spring | Interior stream type | 12_HELLSC | 2014 | 0.9920 | 1982 | 126 |
| OtsEAST_seg_su14 | Eastbank Fish Hatchery | Chelan Falls, Methow River, Okanogan River, Wenatchee River | Columbia | 03Summer | Interior ocean type | 18_UCOLSF | 2014 | 1.0000 | 630 | 127 |
| OtsEAST_seg_sp14 | Eastbank Fish Hatchery | Chelan Falls, Methow River, Okanogan River, Wenatchee River | Columbia | 01Spring | Interior stream type | 10_UCOLSP | 2014 | 1.0000 | 177 | 128 |
| OtsENFH_seg_su14 | Entiat National Fish Hatchery | Entiat | Columbia | 03Summer | Interior ocean type | 18_UCOLSF | 2014 | 0.7331 | 256 | 129 |
| OtsKLIC_seg_sp14 | Klickitat Hatchery | Klickitat River | Columbia | 01Spring | Interior stream type | 06_KLICKR | 2014 | 1.0000 | 399 | 130 |
| OtsLNFH_seg_sp14 | Leavenworth National Fish Hatchery | Mixed origins (Upper-Columbia Rivers) | Columbia | 01Spring | Interior stream type | 10_UCOLSP | 2014 | 1.0000 | 933 | 131 |
| OtsLWSN_seg_sp14 | Little White Salmon National Fish Hatchery | Little White Salmon River | Columbia | 01Spring | Interior stream type | 20_BONPOOLSP | 2014 | 1.0000 | 757 | 132 |
| OtsLWSN_seg_fa14 | Little White Salmon National Fish Hatchery | Little White Salmon River | Columbia | 04Fall | Interior ocean type | 22_BONPOOLFA | 2014 | 1.0000 | 6681 | 133 |
| OtsIMNW_seg_ss14 | Lookingglass Fish Hatchery | Imnaha River | Snake | 02Spring/Summer | Interior stream type | 12_HELLSC | 2014 | 0.9855 | 266 | 134 |
| OtsLOOK_cap_sp14 | Lookingglass Fish Hatchery | Lookingglass Creek | Snake | 02Spring/Summer | Interior stream type | 12_HELLSC | 2014 | 1.0000 | 245 | 135 |
| OtsLOOK_seg_sp14 | Lookingglass Fish Hatchery | Lookingglass/Catherine Creek/Grande Ronde/Lostine River | Snake | 01Spring | Interior stream type | 12_HELLSC | 2014 | 0.8707 | 514 | 136 |
| OtsLYON_seg_fa14 | Lyons Ferry Fish Hatchery | Mixed origins (Snake River and Upper Columbia River) | Snake | 04Fall | Interior ocean type | 19_SRFALL | 2014 | 0.9700 | 1749 | 137 |
| OtsLYON_seg_sp14 | Lyons Ferry Fish Hatchery | Tucannon River | Snake | 01Spring | Interior stream type | 11_TUCANO | 2014 | 0.7727 | 120 | 138 |
| OtsJHNW_int_ss14 | McCall Fish Hatchery | Johnson Creek | Snake | 02Spring/Summer | Interior stream type | 13_SFSALM | 2014 | 1.0000 | 64 | 139 |
| OtsMCCA_seg_ss14 | McCall Fish Hatchery | South Fork Salmon | Snake | 02Spring/Summer | Interior stream type | 13_SFSALM | 2014 | 0.9706 | 473 | 140 |
| OtsMETH_seg_sp14 | Methow Fish Hatchery | Upper Methow and Twisp Rivers | Columbia | 01Spring | Interior stream type | 10_UCOLSP | 2014 | 0.9830 | 116 | 141 |
| OtsNPFH_seg_fa14 | Nez Perce Tribal Fish Hatchery | Mixed origins (Snake River and Clearwater River) | Snake | 04Fall | Interior ocean type | 19_SRFALL | 2014 | 0.9800 | 802 | 142 |
| OtsNPFH_seg_sp14 | Nez Perce | Newsome Creek and Yoosa/Camp Creek | Snake | 01Spring | Interior stream type | 12_HELLSC | 2014 | 0.9926 | 381 | 143 |
| OtsPAHH_seg_ss14 | Pahsimeroi Fish Hatchery | Salmon River | Snake | 02Spring/Summer | Interior stream type | 16_UPSALM | 2014 | 0.9974 | 770 | 144 |
| OtsPARK_seg_sp14 | Parkdale Fish Facility | Hood River | Columbia | 01Spring | Lower Columbia | 02_WCASSP | 2014 | 0.9529 | 164 | 145 |
| OtsPRIE_seg_fa14 | Priest Rapids Hatchery | Mixed origins (Mid-/Upper Columbia Rivers) | Columbia | 04Fall | Interior ocean type | 18_UCOLSF | 2014 | 1.0000 | 5051 | 146 |
| OtsRAPH_seg_sp14 | Rapid River Fish Hatchery | Rapid River | Snake | 01Spring | Interior stream type | 12_HELLSC | 2014 | 0.9959 | 2379 | 147 |
| OtsRBFH_seg_sp14 | Round Butte Fish Hatchery | Deschutes River | Columbia | 01Spring | Interior stream type | 07_DESCSP | 2014 | 1.0000 | 275 | 148 |
| OtsSAWT_seg_ss14 | Sawtooth Fish Hatchery | Salmon River | Snake | 02Spring/Summer | Interior stream type | 16_UPSALM | 2014 | 1.0000 | 931 | 149 |
| OtsUMAT_seg_fa14 | Umatilla Fish Hatchery |  | Columbia | 04Fall | Interior ocean type | 23_UMATILLAFA | 2014 | 1.0000 | 387 | 150 |
| OtsUMAT_seg_sp14 | Umatilla Fish Hatchery |  | Columbia | 01Spring | Interior stream type | 21_UMATILLASP | 2014 | 1.0000 | 451 | 151 |
| OtsWSNF_seg_sp14 | Warm Springs National Fish Hatchery |  | Columbia | 01Spring | Interior stream type | 07_DESCSP | 2014 | 0.3215 | 110 | 152 |
| OtsWELL_seg_su14 | Wells Fish Hatchery |  | Columbia | 03Summer | Interior ocean type | 18_UCOLSF | 2014 | 1.0000 | 658 | 153 |
| OtsWINT_seg_sp14 | Winthrop National Fish Hatchery |  | Columbia | 01Spring | Interior stream type | 10_UCOLSP | 2014 | 0.9312 | 386 | 154 |
| OtsYRRD_int_sp14 | Yakima River Roza Dam | Integrated | Columbia | 01Spring | Interior stream type | 09_YAKIMA | 2014 | 1.0000 | 384 | 155 |
| OtsYRRD_seg_sp14 | Yakima River Roza Dam | Segregated | Columbia | 01Spring | Interior stream type | 09_YAKIMA | 2014 | 0.5301 | 83 | 156 |
| OtsBIGC_seg_fa15 | Big Creek Hatchery |  | Lower Columbia | 04Fall | Lower Columbia | 03_WCASFA | 2015 | 0.1608 | 670 | 157 |
| OtsCARS_seg_sp15 | Carson National Fish Hatchery | Mixed origins (Snake and Mid-/Upper-Columbia Rivers) | Columbia | 01Spring | Interior stream type | 20_BONPOOLSP | 2015 | 0.9722 | 986 | 158 |
| OtsCHJO_int_su15 | Chief Joseph Hatchery | Integrated | Columbia | 03Summer | Interior ocean type | 18_UCOLSF | 2015 | 0.8910 | 404 | 159 |
| OtsCHJO_seg_sp15 | Chief Joseph Hatchery | Spring | Columbia | 01Spring | Interior stream type | 10_UCOLSP | 2015 | 0.9967 | 609 | 160 |
| OtsCHJO_seg_su15 | Chief Joseph Hatchery | Segregated | Columbia | 03Summer | Interior ocean type | 18_UCOLSF | 2015 | 0.9584 | 326 | 161 |
| OtsCLWH_seg_sp15 | Clearwater Fish Hatchery | Clearwater River | Snake | 01Spring | Interior stream type | 12_HELLSC | 2015 | 0.9932 | 1149 | 162 |
| OtsPOWP_seg_sp15 | Clearwater Fish Hatchery | Lochsa River (Powell Facility) | Snake | 01Spring | Interior stream type | 12_HELLSC | 2015 | 0.9711 | 470 | 163 |
| OtsCOWL_seg_fa15 | Cowlitz Salmon Hatchery |  | Lower Columbia | 04Fall | Lower Columbia | 03_WCASFA | 2015 | 0.9700 | 1822 | 164 |
| OtsCOWL_seg_sp15 | Cowlitz Salmon Hatchery |  | Lower Columbia | 01Spring | Lower Columbia | 02_WCASSP | 2015 | 0.8814 | 1350 | 165 |
| OtsDWOR_seg_sp15 | Dworshak National Fish Hatchery | Clearwater River | Snake | 01Spring | Interior stream type | 12_HELLSC | 2015 | 0.9877 | 2190 | 166 |
| OtsEAST_seg_su15 | Eastbank Fish Hatchery | Chelan Falls, Methow River, Okanogan River, Wenatchee River | Columbia | 03Summer | Interior ocean type | 18_UCOLSF | 2015 | 1.0000 | 634 | 167 |
| OtsEAST_seg_sp15 | Eastbank Fish Hatchery | Chelan Falls, Methow River, Okanogan River, Wenatchee River | Columbia | 01Spring | Interior stream type | 10_UCOLSP | 2015 | 1.0000 | 240 | 168 |
| OtsENFH_seg_su15 | Entiat National Fish Hatchery | Entiat | Columbia | 03Summer | Interior ocean type | 18_UCOLSF | 2015 | 0.7290 | 257 | 169 |
| OtsKALA_seg_sp15 | Kalama Falls Hatchery |  | Lower Columbia | 01Spring | Lower Columbia | 02_WCASSP | 2015 | 0.9145 | 350 | 170 |
| OtsKLIC_seg_sp15 | Klickitat Hatchery | Klickitat River | Columbia | 01Spring | Interior stream type | 06_KLICKR | 2015 | 1.0000 | 427 | 171 |
| OtsLNFH_seg_sp15 | Leavenworth National Fish Hatchery | Mixed origins (Upper-Columbia Rivers) | Columbia | 01Spring | Interior stream type | 10_UCOLSP | 2015 | 0.8472 | 879 | 172 |
| OtsLWSN_seg_sp15 | Little White Salmon National Fish Hatchery | Little White Salmon River | Columbia | 01Spring | Interior stream type | 20_BONPOOLSP | 2015 | 1.0000 | 612 | 173 |
| OtsLWSN_seg_fa15 | Little White Salmon National Fish Hatchery | Little White Salmon River | Columbia | 04Fall | Interior ocean type | 22_BONPOOLFA | 2015 | 0.9514 | 8048 | 174 |
| OtsIMNW_seg_ss15 | Lookingglass Fish Hatchery | Imnaha River | Snake | 02Spring/Summer | Interior stream type | 12_HELLSC | 2015 | 0.9344 | 240 | 175 |
| OtsLOOK_seg_sp15 | Lookingglass Fish Hatchery | Lookingglass/Catherine Creek/Grande Ronde/Lostine River | Snake | 01Spring | Interior stream type | 12_HELLSC | 2015 | 0.9138 | 555 | 176 |
| OtsLYON_seg_fa15 | Lyons Ferry Fish Hatchery | Mixed origins (Snake River and Upper Columbia River) | Snake | 04Fall | Interior ocean type | 19_SRFALL | 2015 | 0.9300 | 1751 | 177 |
| OtsLYON_seg_sp15 | Lyons Ferry Fish Hatchery | Tucannon River | Snake | 01Spring | Interior stream type | 11_TUCANO | 2015 | 0.3200 | 101 | 178 |
| OtsJHNW_int_ss15 | McCall Fish Hatchery | Johnson Creek | Snake | 02Spring/Summer | Interior stream type | 13_SFSALM | 2015 | 0.9310 | 70 | 179 |
| OtsMCCA_seg_ss15 | McCall Fish Hatchery | South Fork Salmon | Snake | 02Spring/Summer | Interior stream type | 13_SFSALM | 2015 | 0.9740 | 1181 | 180 |
| OtsMETH_seg_sp15 | Methow Fish Hatchery | Upper Methow and Twisp Rivers | Columbia | 01Spring | Interior stream type | 10_UCOLSP | 2015 | 1.0000 | 117 | 181 |
| OtsNPFH_seg_fa15 | Nez Perce Tribal Fish Hatchery | Mixed origins (Snake River and Clearwater River) | Snake | 04Fall | Interior ocean type | 19_SRFALL | 2015 | 0.9660 | 822 | 182 |
| OtsNPFH_seg_sp15 | Nez Perce | Newsome Creek and Yoosa/Camp Creek | Snake | 01Spring | Interior stream type | 12_HELLSC | 2015 | 0.9553 | 448 | 183 |
| OtsNTOU_seg_fa15 | North Toutle Hatchery |  | Lower Columbia | 04Fall | Lower Columbia | 03_WCASFA | 2015 | 0.8699 | 748 | 184 |
| OtsPAHH_seg_ss15 | Pahsimeroi Fish Hatchery | Salmon River | Snake | 02Spring/Summer | Interior stream type | 16_UPSALM | 2015 | 0.9611 | 1011 | 185 |
| OtsPARK_seg_sp15 | Parkdale Fish Facility | Hood River | Columbia | 01Spring | Lower Columbia | 02_WCASSP | 2015 | 0.9564 | 222 | 186 |
| OtsPRIE_seg_fa15 | Priest Rapids Hatchery | Mixed origins (Mid-/Upper Columbia Rivers) | Columbia | 04Fall | Interior ocean type | 18_UCOLSF | 2015 | 1.0000 | 5051 | 187 |
| OtsRAPH_seg_sp15 | Rapid River Fish Hatchery | Rapid River | Snake | 01Spring | Interior stream type | 12_HELLSC | 2015 | 0.9378 | 1843 | 188 |
| OtsRBFH_seg_sp15 | Round Butte Fish Hatchery | Deschutes River | Columbia | 01Spring | Interior stream type | 07_DESCSP | 2015 | 0.9276 | 888 | 189 |
| OtsSAWT_seg_ss15 | Sawtooth Fish Hatchery | Salmon River | Snake | 02Spring/Summer | Interior stream type | 16_UPSALM | 2015 | 0.9908 | 865 | 190 |
| OtsSSAN_seg_sp15 | South Santiam Hatchery |  | Willamette | 01Spring | Lower Columbia | 04_WILLAM | 2015 | 0.8370 | 967 | 191 |
| OtsSPEE_seg_sp15 | Speelyai Fish Hatchery |  | Lower Columbia | 01Spring | Lower Columbia | 02_WCASSP | 2015 | 0.4986 | 358 | 192 |
| OtsUMAT_seg_fa15 | Umatilla Fish Hatchery |  | Columbia | 04Fall | Interior ocean type | 23_UMATILLAFA | 2015 | 0.0000 | 0 | 193 |
| OtsUMAT_seg_sp15 | Umatilla Fish Hatchery |  | Columbia | 01Spring | Interior stream type | 21_UMATILLASP | 2015 | 1.0000 | 480 | 194 |
| OtsWSNF_seg_sp15 | Warm Springs National Fish Hatchery |  | Columbia | 01Spring | Interior stream type | 07_DESCSP | 2015 | 0.1815 | 262 | 195 |
| OtsWASH_seg_fa15 | Washougal Fish Hatchery |  | Lower Columbia | 04Fall | Lower Columbia | 03_WCASFA | 2015 | 0.2195 | 1085 | 196 |
| OtsWELL_seg_su15 | Wells Fish Hatchery |  | Columbia | 03Summer | Interior ocean type | 18_UCOLSF | 2015 | 1.0000 | 713 | 197 |
| OtsWINT_seg_sp15 | Winthrop National Fish Hatchery |  | Columbia | 01Spring | Interior stream type | 10_UCOLSP | 2015 | 1.0000 | 374 | 198 |
| OtsPROS_seg_fa15 | Yakima Nation Prosser Hatchery |  | Columbia | 04Fall | Interior ocean type | 18_UCOLSF | 2015 | 1.0000 | 350 | 199 |
| OtsYRRD_int_sp15 | Yakima River Roza Dam | Integrated | Columbia | 01Spring | Interior stream type | 09_YAKIMA | 2015 | 0.9732 | 438 | 200 |
| OtsYRRD_seg_sp15 | Yakima River Roza Dam | Segregated | Columbia | 01Spring | Interior stream type | 09_YAKIMA | 2015 | 0.8948 | 35 | 201 |
| OtsSPCR_seg_fa15 | Spring Creek National Fish Hatchery |  | Columbia | 04Fall | Lower Columbia | 05_SPCRTU | 2015 | 0.9704 | 5553 | 202 |
| OtsBIGC_seg_fa16 | Big Creek Hatchery |  | Lower Columbia | 04Fall | Lower Columbia | 03_WCASFA | 2016 | 0.1987 | 575 | 203 |
| OtsCARS_seg_sp16 | Carson National Fish Hatchery | Mixed origins (Snake and Mid-/Upper-Columbia Rivers) | Columbia | 01Spring | Interior stream type | 20_BONPOOLSP | 2016 | 1.0000 | 1027 | 204 |
| OtsCHJO_int_su16 | Chief Joseph Hatchery | Integrated | Columbia | 03Summer | Interior ocean type | 18_UCOLSF | 2016 | 0.9896 | 575 | 205 |
| OtsCHJO_seg_sp16 | Chief Joseph Hatchery | Spring | Columbia | 01Spring | Interior stream type | 10_UCOLSP | 2016 | 0.9754 | 559 | 206 |
| OtsCHJO_seg_su16 | Chief Joseph Hatchery | Segregated | Columbia | 03Summer | Interior ocean type | 18_UCOLSF | 2016 | 0.9915 | 469 | 207 |
| OtsCLWH_seg_sp16 | Clearwater Fish Hatchery | Clearwater River | Snake | 01Spring | Interior stream type | 12_HELLSC | 2016 | 0.9854 | 1475 | 208 |
| OtsPOWP_seg_sp16 | Clearwater Fish Hatchery | Lochsa River (Powell Facility) | Snake | 01Spring | Interior stream type | 12_HELLSC | 2016 | 0.9921 | 341 | 209 |
| OtsCOWL_seg_fa16 | Cowlitz Salmon Hatchery |  | Lower Columbia | 04Fall | Lower Columbia | 03_WCASFA | 2016 | 0.7906 | 1532 | 210 |
| OtsCOWL_seg_sp16 | Cowlitz Salmon Hatchery |  | Lower Columbia | 01Spring | Lower Columbia | 02_WCASSP | 2016 | 0.4054 | 673 | 211 |
| OtsDWOR_seg_sp16 | Dworshak National Fish Hatchery | Clearwater River | Snake | 01Spring | Interior stream type | 12_HELLSC | 2016 | 0.9849 | 1883 | 212 |
| OtsEAST_seg_su16 | Eastbank Fish Hatchery | Chelan Falls, Methow River, Okanogan River, Wenatchee River | Columbia | 03Summer | Interior ocean type | 18_UCOLSF | 2016 | 0.9831 | 699 | 213 |
| OtsEAST_seg_sp16 | Eastbank Fish Hatchery | Chelan Falls, Methow River, Okanogan River, Wenatchee River | Columbia | 01Spring | Interior stream type | 10_UCOLSP | 2016 | 0.9032 | 249 | 214 |
| OtsENFH_seg_su16 | Entiat National Fish Hatchery | Entiat | Columbia | 03Summer | Interior ocean type | 18_UCOLSF | 2016 | 1.0000 | 292 | 215 |
| OtsKALA_seg_sp16 | Kalama Falls Hatchery |  | Lower Columbia | 01Spring | Lower Columbia | 02_WCASSP | 2016 | 0.7307 | 318 | 216 |
| OtsKALA_seg_fa16 | Kalama Falls Hatchery |  | Lower Columbia | 04Fall | Lower Columbia | 03_WCASFA | 2016 | 0.6348 | 2803 | 217 |
| OtsKLIC_seg_sp16 | Klickitat Hatchery | Klickitat River | Columbia | 01Spring | Interior stream type | 06_KLICKR | 2016 | 1.0000 | 476 | 218 |
| OtsLNFH_seg_sp16 | Leavenworth National Fish Hatchery | Mixed origins (Upper-Columbia Rivers) | Columbia | 01Spring | Interior stream type | 10_UCOLSP | 2016 | 1.0000 | 885 | 219 |
| OtsLWSN_seg_sp16 | Little White Salmon National Fish Hatchery | Little White Salmon River | Columbia | 01Spring | Interior stream type | 20_BONPOOLSP | 2016 | 0.9349 | 730 | 220 |
| OtsLWSN_seg_fa16 | Little White Salmon National Fish Hatchery | Little White Salmon River | Columbia | 04Fall | Interior ocean type | 22_BONPOOLFA | 2016 | 1.0000 | 7313 | 221 |
| OtsIMNW_seg_ss16 | Lookingglass Fish Hatchery | Imnaha River | Snake | 02Spring/Summer | Interior stream type | 12_HELLSC | 2016 | 0.9909 | 243 | 222 |
| OtsLOOK_seg_sp16 | Lookingglass Fish Hatchery | Lookingglass/Catherine Creek/Grande Ronde/Lostine River | Snake | 01Spring | Interior stream type | 12_HELLSC | 2016 | 0.8923 | 484 | 223 |
| OtsLYON_seg_fa16 | Lyons Ferry Fish Hatchery | Mixed origins (Snake River and Upper Columbia River) | Snake | 04Fall | Interior ocean type | 19_SRFALL | 2016 | 0.9435 | 1912 | 224 |
| OtsLYON_seg_sp16 | Lyons Ferry Fish Hatchery | Tucannon River | Snake | 01Spring | Interior stream type | 11_TUCANO | 2016 | 0.8472 | 122 | 225 |
| OtsJHNW_int_ss16 | McCall Fish Hatchery | Johnson Creek | Snake | 02Spring/Summer | Interior stream type | 13_SFSALM | 2016 | 0.9411 | 65 | 226 |
| OtsMCCA_seg_ss16 | McCall Fish Hatchery | South Fork Salmon | Snake | 02Spring/Summer | Interior stream type | 13_SFSALM | 2016 | 0.9889 | 844 | 227 |
| OtsMETH_seg_sp16 | Methow Fish Hatchery | Upper Methow and Twisp Rivers | Columbia | 01Spring | Interior stream type | 10_UCOLSP | 2016 | 1.0000 | 112 | 228 |
| OtsNPFH_seg_fa16 | Nez Perce Tribal Fish Hatchery | Mixed origins (Snake River and Clearwater River) | Snake | 04Fall | Interior ocean type | 19_SRFALL | 2016 | 0.9726 | 752 | 229 |
| OtsNPFH_seg_sp16 | Nez Perce | Newsome Creek and Yoosa/Camp Creek | Snake | 01Spring | Interior stream type | 12_HELLSC | 2016 | 0.9919 | 556 | 230 |
| OtsNTOU_seg_fa16 | North Toutle Hatchery |  | Lower Columbia | 04Fall | Lower Columbia | 03_WCASFA | 2016 | 0.9110 | 629 | 231 |
| OtsPAHH_seg_ss16 | Pahsimeroi Fish Hatchery | Salmon River | Snake | 02Spring/Summer | Interior stream type | 16_UPSALM | 2016 | 0.9973 | 691 | 232 |
| OtsPARK_seg_sp16 | Parkdale Fish Facility | Hood River | Columbia | 01Spring | Lower Columbia | 02_WCASSP | 2016 | 0.9450 | 279 | 233 |
| OtsPRIE_seg_fa16 | Priest Rapids Hatchery | Mixed origins (Mid-/Upper Columbia Rivers) | Columbia | 04Fall | Interior ocean type | 18_UCOLSF | 2016 | 1.0000 | 4844 | 234 |
| OtsRAPH_seg_sp16 | Rapid River Fish Hatchery | Rapid River | Snake | 01Spring | Interior stream type | 12_HELLSC | 2016 | 0.9779 | 2061 | 235 |
| OtsRBFH_seg_sp16 | Round Butte Fish Hatchery | Deschutes River | Columbia | 01Spring | Interior stream type | 07_DESCSP | 2016 | 1.0000 | 313 | 236 |
| OtsSAWT_seg_ss16 | Sawtooth Fish Hatchery | Salmon River | Snake | 02Spring/Summer | Interior stream type | 16_UPSALM | 2016 | 0.9961 | 1058 | 237 |
| OtsSSAN_seg_sp16 | South Santiam Hatchery |  | Willamette | 01Spring | Lower Columbia | 04_WILLAM | 2016 | 0.9410 | 972 | 238 |
| OtsSPEE_seg_sp16 | Speelyai Fish Hatchery |  | Lower Columbia | 01Spring | Lower Columbia | 02_WCASSP | 2016 | 0.6520 | 260 | 239 |
| OtsUMAT_seg_fa16 | Umatilla Fish Hatchery |  | Columbia | 04Fall | Interior ocean type | 23_UMATILLAFA | 2016 | 0.0000 | 0 | 240 |
| OtsUMAT_seg_sp16 | Umatilla Fish Hatchery |  | Columbia | 01Spring | Interior stream type | 21_UMATILLASP | 2016 | 1.0000 | 464 | 241 |
| OtsWSNF_seg_sp16 | Warm Springs National Fish Hatchery |  | Columbia | 01Spring | Interior stream type | 07_DESCSP | 2016 | 0.9874 | 627 | 242 |
| OtsWASH_seg_fa16 | Washougal Fish Hatchery |  | Lower Columbia | 04Fall | Lower Columbia | 03_WCASFA | 2016 | 0.4356 | 1122 | 243 |
| OtsWELL_seg_su16 | Wells Fish Hatchery |  | Columbia | 03Summer | Interior ocean type | 18_UCOLSF | 2016 | 1.0000 | 721 | 244 |
| OtsWINT_seg_sp16 | Winthrop National Fish Hatchery |  | Columbia | 01Spring | Interior stream type | 10_UCOLSP | 2016 | 1.0000 | 280 | 245 |
| OtsPROS_seg_fa16 | Yakima Nation Prosser Hatchery |  | Columbia | 04Fall | Interior ocean type | 18_UCOLSF | 2016 | 1.0000 | 257 | 246 |
| OtsYRRD_int_sp16 | Yakima River Roza Dam | Integrated | Columbia | 01Spring | Interior stream type | 09_YAKIMA | 2016 | 0.7232 | 324 | 247 |
| OtsYRRD_seg_sp16 | Yakima River Roza Dam | Segregated | Columbia | 01Spring | Interior stream type | 09_YAKIMA | 2016 | 0.6481 | 128 | 248 |
| OtsRING_seg_fa16 | Ringold Springs State Hatchery |  | Columbia | 04Fall | Interior ocean type | 18_UCOLSF | 2016 | 1.0000 | 985 | 249 |
| OtsSPCR_seg_fa16 | Spring Creek National Fish Hatchery |  | Columbia | 04Fall | Lower Columbia | 05_SPCRTU | 2016 | 0.6343 | 4652 | 250 |
| OtsBIGC_seg_fa17 | Big Creek Hatchery |  | Lower Columbia | 04Fall | Lower Columbia | 03_WCASFA | 2017 | 0.8307 | 1708 | 251 |
| OtsCARS_seg_sp17 | Carson National Fish Hatchery | Mixed origins (Snake and Mid-/Upper-Columbia Rivers) | Columbia | 01Spring | Interior stream type | 20_BONPOOLSP | 2017 | 0.9841 | 993 | 252 |
| OtsCHJO_int_su17 | Chief Joseph Hatchery | Integrated | Columbia | 03Summer | Interior ocean type | 18_UCOLSF | 2017 | 0.7549 | 318 | 253 |
| OtsCHJO_seg_sp17 | Chief Joseph Hatchery | Spring | Columbia | 01Spring | Interior stream type | 10_UCOLSP | 2017 | 0.9209 | 523 | 254 |
| OtsCHJO_seg_su17 | Chief Joseph Hatchery | Segregated | Columbia | 03Summer | Interior ocean type | 18_UCOLSF | 2017 | 0.9032 | 364 | 255 |
| OtsCLWH_seg_sp17 | Clearwater Fish Hatchery | Clearwater River | Snake | 01Spring | Interior stream type | 12_HELLSC | 2017 | 0.9897 | 1623 | 256 |
| OtsPOWP_seg_sp17 | Clearwater Fish Hatchery | Lochsa River (Powell Facility) | Snake | 01Spring | Interior stream type | 12_HELLSC | 2017 | 0.9858 | 435 | 257 |
| OtsCOWL_seg_fa17 | Cowlitz Salmon Hatchery |  | Lower Columbia | 04Fall | Lower Columbia | 03_WCASFA | 2017 | 0.8090 | 1726 | 258 |
| OtsCOWL_seg_sp17 | Cowlitz Salmon Hatchery |  | Lower Columbia | 01Spring | Lower Columbia | 02_WCASSP | 2017 | 0.9369 | 1601 | 259 |
| OtsDWOR_seg_sp17 | Dworshak National Fish Hatchery | Clearwater River | Snake | 01Spring | Interior stream type | 12_HELLSC | 2017 | 0.9924 | 969 | 260 |
| OtsKOOS_seg_sp17 | Dworshak National Fish Hatchery | Clearwater River | Snake | 01Spring | Interior stream type | 12_HELLSC | 2017 | 0.9941 | 667 | 261 |
| OtsEAST_seg_su17 | Eastbank Fish Hatchery | Chelan Falls, Methow River, Okanogan River, Wenatchee River | Columbia | 03Summer | Interior ocean type | 18_UCOLSF | 2017 | 0.9661 | 690 | 262 |
| OtsEAST_seg_sp17 | Eastbank Fish Hatchery | Chelan Falls, Methow River, Okanogan River, Wenatchee River | Columbia | 01Spring | Interior stream type | 10_UCOLSP | 2017 | 0.9811 | 209 | 263 |
| OtsENFH_seg_su17 | Entiat National Fish Hatchery | Entiat | Columbia | 03Summer | Interior ocean type | 18_UCOLSF | 2017 | 0.8393 | 273 | 264 |
| OtsKALA_seg_sp17 | Kalama Falls Hatchery |  | Lower Columbia | 01Spring | Lower Columbia | 02_WCASSP | 2017 | 0.9707 | 334 | 265 |
| OtsKALA_seg_fa17 | Kalama Falls Hatchery |  | Lower Columbia | 04Fall | Lower Columbia | 03_WCASFA | 2017 | 0.7518 | 2974 | 266 |
| OtsKLIC_seg_sp17 | Klickitat Hatchery | Klickitat River | Columbia | 01Spring | Interior stream type | 06_KLICKR | 2017 | 0.9757 | 484 | 267 |
| OtsLNFH_seg_sp17 | Leavenworth National Fish Hatchery | Mixed origins (Upper-Columbia Rivers) | Columbia | 01Spring | Interior stream type | 10_UCOLSP | 2017 | 0.8606 | 744 | 268 |
| OtsLWSN_seg_sp17 | Little White Salmon National Fish Hatchery | Little White Salmon River | Columbia | 01Spring | Interior stream type | 20_BONPOOLSP | 2017 | 0.9418 | 559 | 269 |
| OtsLWSN_seg_fa17 | Little White Salmon National Fish Hatchery | Little White Salmon River | Columbia | 04Fall | Interior ocean type | 22_BONPOOLFA | 2017 | 0.9595 | 5364 | 270 |
| OtsIMNW_seg_ss17 | Lookingglass Fish Hatchery | Imnaha River | Snake | 02Spring/Summer | Interior stream type | 12_HELLSC | 2017 | 0.9040 | 228 | 271 |
| OtsLOOK_seg_sp17 | Lookingglass Fish Hatchery | Lookingglass/Catherine Creek/Grande Ronde/Lostine River | Snake | 01Spring | Interior stream type | 12_HELLSC | 2017 | 0.8931 | 436 | 272 |
| OtsLYON_seg_fa17 | Lyons Ferry Fish Hatchery | Mixed origins (Snake River and Upper Columbia River) | Snake | 04Fall | Interior ocean type | 19_SRFALL | 2017 | 0.7641 | 1701 | 273 |
| OtsLYON_seg_sp17 | Lyons Ferry Fish Hatchery | Tucannon River | Snake | 01Spring | Interior stream type | 11_TUCANO | 2017 | 0.9455 | 115 | 274 |
| OtsJHNW_int_ss17 | McCall Fish Hatchery | Johnson Creek | Snake | 02Spring/Summer | Interior stream type | 13_SFSALM | 2017 | 1.0000 | 61 | 275 |
| OtsMCCA_seg_ss17 | McCall Fish Hatchery | South Fork Salmon | Snake | 02Spring/Summer | Interior stream type | 13_SFSALM | 2017 | 0.9775 | 806 | 276 |
| OtsMETH_seg_sp17 | Methow Fish Hatchery | Upper Methow and Twisp Rivers | Columbia | 01Spring | Interior stream type | 10_UCOLSP | 2017 | 0.9051 | 137 | 277 |
| OtsNPFH_seg_fa17 | Nez Perce Tribal Fish Hatchery | Mixed origins (Snake River and Clearwater River) | Snake | 04Fall | Interior ocean type | 19_SRFALL | 2017 | 0.9681 | 673 | 278 |
| OtsNPFH_seg_sp17 | Nez Perce | Newsome Creek and Yoosa/Camp Creek | Snake | 01Spring | Interior stream type | 12_HELLSC | 2017 | 0.8034 | 383 | 279 |
| OtsNTOU_seg_fa17 | North Toutle Hatchery |  | Lower Columbia | 04Fall | Lower Columbia | 03_WCASFA | 2017 | 0.7854 | 148 | 280 |
| OtsPAHH_seg_ss17 | Pahsimeroi Fish Hatchery | Salmon River | Snake | 02Spring/Summer | Interior stream type | 16_UPSALM | 2017 | 0.9952 | 368 | 281 |
| OtsPARK_seg_sp17 | Parkdale Fish Facility | Hood River | Columbia | 01Spring | Lower Columbia | 02_WCASSP | 2017 | 0.9341 | 173 | 282 |
| OtsPRIE_seg_fa17 | Priest Rapids Hatchery | Mixed origins (Mid-/Upper Columbia Rivers) | Columbia | 04Fall | Interior ocean type | 18_UCOLSF | 2017 | 0.9095 | 6441 | 283 |
| OtsRAPH_seg_sp17 | Rapid River Fish Hatchery | Rapid River | Snake | 01Spring | Interior stream type | 12_HELLSC | 2017 | 0.9939 | 2560 | 284 |
| OtsRBFH_seg_sp17 | Round Butte Fish Hatchery | Deschutes River | Columbia | 01Spring | Interior stream type | 07_DESCSP | 2017 | 0.8889 | 841 | 285 |
| OtsSAWT_seg_ss17 | Sawtooth Fish Hatchery | Salmon River | Snake | 02Spring/Summer | Interior stream type | 16_UPSALM | 2017 | 0.9970 | 665 | 286 |
| OtsSSAN_seg_sp17 | South Santiam Hatchery |  | Willamette | 01Spring | Lower Columbia | 04_WILLAM | 2017 | 0.0000 | 0 | 287 |
| OtsSPEE_seg_sp17 | Speelyai Fish Hatchery |  | Lower Columbia | 01Spring | Lower Columbia | 02_WCASSP | 2017 | 0.5727 | 728 | 288 |
| OtsUMAT_seg_fa17 | Umatilla Fish Hatchery |  | Columbia | 04Fall | Interior ocean type | 23_UMATILLAFA | 2017 | 0.0000 | 0 | 289 |
| OtsUMAT_seg_sp17 | Umatilla Fish Hatchery |  | Columbia | 01Spring | Interior stream type | 21_UMATILLASP | 2017 | 1.0000 | 511 | 290 |
| OtsWSNF_seg_sp17 | Warm Springs National Fish Hatchery |  | Columbia | 01Spring | Interior stream type | 07_DESCSP | 2017 | 0.7951 | 667 | 291 |
| OtsWASH_seg_fa17 | Washougal Fish Hatchery |  | Lower Columbia | 04Fall | Lower Columbia | 03_WCASFA | 2017 | 0.5004 | 822 | 292 |
| OtsWELL_seg_su17 | Wells Fish Hatchery |  | Columbia | 03Summer | Interior ocean type | 18_UCOLSF | 2017 | 0.8901 | 534 | 293 |
| OtsWINT_seg_sp17 | Winthrop National Fish Hatchery |  | Columbia | 01Spring | Interior stream type | 10_UCOLSP | 2017 | 0.9445 | 414 | 294 |
| OtsPROS_seg_fa17 | Yakima Nation Prosser Hatchery |  | Columbia | 04Fall | Interior ocean type | 18_UCOLSF | 2017 | 0.3964 | 119 | 295 |
| OtsYRRD_int_sp17 | Yakima River Roza Dam | Integrated | Columbia | 01Spring | Interior stream type | 09_YAKIMA | 2017 | 0.9943 | 350 | 296 |
| OtsYRRD_seg_sp17 | Yakima River Roza Dam | Segregated | Columbia | 01Spring | Interior stream type | 09_YAKIMA | 2017 | 0.9807 | 102 | 297 |
| OtsRING_seg_fa17 | Ringold Springs State Hatchery |  | Columbia | 04Fall | Interior ocean type | 18_UCOLSF | 2017 | 0.9340 | 1583 | 298 |
| OtsSPCR_seg_fa17 | Spring Creek National Fish Hatchery |  | Columbia | 04Fall | Lower Columbia | 05_SPCRTU | 2017 | 0.5992 | 4063 | 299 |
| OtsBIGC_seg_fa18 | Big Creek Hatchery |  | Lower Columbia | 04Fall | Lower Columbia | 03_WCASFA | 2018 | 0.7120 | 1804 | 300 |
| OtsCARS_seg_sp18 | Carson National Fish Hatchery | Mixed origins (Snake and Mid-/Upper-Columbia Rivers) | Columbia | 01Spring | Interior stream type | 20_BONPOOLSP | 2018 | 1.0000 | 929 | 301 |
| OtsCHJO_int_su18 | Chief Joseph Hatchery | Integrated | Columbia | 03Summer | Interior ocean type | 18_UCOLSF | 2018 | 1.0000 | 426 | 302 |
| OtsCHJO_seg_sp18 | Chief Joseph Hatchery | Spring | Columbia | 01Spring | Interior stream type | 10_UCOLSP | 2018 | 0.5658 | 170 | 303 |
| OtsCHJO_seg_su18 | Chief Joseph Hatchery | Segregated | Columbia | 03Summer | Interior ocean type | 18_UCOLSF | 2018 | 1.0000 | 311 | 304 |
| OtsCLWH_seg_sp18 | Clearwater Fish Hatchery | Clearwater River | Snake | 01Spring | Interior stream type | 12_HELLSC | 2018 | 1.0000 | 1075 | 305 |
| OtsPOWP_seg_sp18 | Clearwater Fish Hatchery | Lochsa River (Powell Facility) | Snake | 01Spring | Interior stream type | 12_HELLSC | 2018 | 0.9958 | 356 | 306 |
| OtsCOWL_seg_fa18 | Cowlitz Salmon Hatchery |  | Lower Columbia | 04Fall | Lower Columbia | 03_WCASFA | 2018 | 0.5904 | 783 | 307 |
| OtsCOWL_seg_sp18 | Cowlitz Salmon Hatchery |  | Lower Columbia | 01Spring | Lower Columbia | 02_WCASSP | 2018 | 0.8438 | 1399 | 308 |
| OtsDWOR_seg_sp18 | Dworshak National Fish Hatchery | Clearwater River | Snake | 01Spring | Interior stream type | 12_HELLSC | 2018 | 0.9493 | 798 | 309 |
| OtsKOOS_seg_sp18 | Dworshak National Fish Hatchery | Clearwater River | Snake | 01Spring | Interior stream type | 12_HELLSC | 2018 | 0.9429 | 820 | 310 |
| OtsEAST_seg_su18 | Eastbank Fish Hatchery | Chelan Falls, Methow River, Okanogan River, Wenatchee River | Columbia | 03Summer | Interior ocean type | 18_UCOLSF | 2018 | 0.9917 | 718 | 311 |
| OtsEAST_seg_sp18 | Eastbank Fish Hatchery | Chelan Falls, Methow River, Okanogan River, Wenatchee River | Columbia | 01Spring | Interior stream type | 10_UCOLSP | 2018 | 1.0000 | 209 | 312 |
| OtsENFH_seg_su18 | Entiat National Fish Hatchery | Entiat | Columbia | 03Summer | Interior ocean type | 18_UCOLSF | 2018 | 0.7628 | 269 | 313 |
| OtsKALA_seg_sp18 | Kalama Falls Hatchery |  | Lower Columbia | 01Spring | Lower Columbia | 02_WCASSP | 2018 | 0.5941 | 686 | 314 |
| OtsKALA_seg_fa18 | Kalama Falls Hatchery |  | Lower Columbia | 04Fall | Lower Columbia | 03_WCASFA | 2018 | 0.8585 | 3167 | 315 |
| OtsKLIC_seg_sp18 | Klickitat Hatchery | Klickitat River | Columbia | 01Spring | Interior stream type | 06_KLICKR | 2018 | 0.9665 | 291 | 316 |
| OtsKLIC_seg_fa18 | Klickitat Hatchery | Little White Salmon River | Columbia | 04Fall | Interior ocean type | 22_BONPOOLFA | 2018 | 0.5420 | 187 | 317 |
| OtsLNFH_seg_sp18 | Leavenworth National Fish Hatchery | Mixed origins (Upper-Columbia Rivers) | Columbia | 01Spring | Interior stream type | 10_UCOLSP | 2018 | 0.5506 | 535 | 318 |
| OtsLWSN_seg_sp18 | Little White Salmon National Fish Hatchery | Little White Salmon River | Columbia | 01Spring | Interior stream type | 20_BONPOOLSP | 2018 | 0.9392 | 1413 | 319 |
| OtsLWSN_seg_fa18 | Little White Salmon National Fish Hatchery | Little White Salmon River | Columbia | 04Fall | Interior ocean type | 22_BONPOOLFA | 2018 | 0.7313 | 1730 | 320 |
| OtsIMNW_seg_ss18 | Lookingglass Fish Hatchery | Imnaha River | Snake | 02Spring/Summer | Interior stream type | 12_HELLSC | 2018 | 0.9411 | 216 | 321 |
| OtsLOOK_seg_sp18 | Lookingglass Fish Hatchery | Lookingglass/Catherine Creek/Grande Ronde/Lostine River | Snake | 01Spring | Interior stream type | 12_HELLSC | 2018 | 0.9276 | 497 | 322 |
| OtsLYON_seg_fa18 | Lyons Ferry Fish Hatchery | Mixed origins (Snake River and Upper Columbia River) | Snake | 04Fall | Interior ocean type | 19_SRFALL | 2018 | 0.8055 | 1655 | 323 |
| OtsLYON_seg_sp18 | Lyons Ferry Fish Hatchery | Tucannon River | Snake | 01Spring | Interior stream type | 11_TUCANO | 2018 | 0.6447 | 131 | 324 |
| OtsJHNW_int_ss18 | McCall Fish Hatchery | Johnson Creek | Snake | 02Spring/Summer | Interior stream type | 13_SFSALM | 2018 | 1.0000 | 95 | 325 |
| OtsMCCA_seg_ss18 | McCall Fish Hatchery | South Fork Salmon | Snake | 02Spring/Summer | Interior stream type | 13_SFSALM | 2018 | 0.9879 | 741 | 326 |
| OtsMETH_seg_sp18 | Methow Fish Hatchery | Upper Methow and Twisp Rivers | Columbia | 01Spring | Interior stream type | 10_UCOLSP | 2018 | 0.7778 | 128 | 327 |
| OtsNPFH_seg_fa18 | Nez Perce Tribal Fish Hatchery | Mixed origins (Snake River and Clearwater River) | Snake | 04Fall | Interior ocean type | 19_SRFALL | 2018 | 0.8115 | 554 | 328 |
| OtsNPFH_seg_sp18 | Nez Perce | Newsome Creek and Yoosa/Camp Creek | Snake | 01Spring | Interior stream type | 12_HELLSC | 2018 | 0.9347 | 183 | 329 |
| OtsNSAN_seg_sp18 | North Santiam Hatchery |  | Willamette | 01Spring | Lower Columbia | 04_WILLAM | 2018 | 0.6524 | 777 | 330 |
| OtsNTOU_seg_fa18 | North Toutle Hatchery |  | Lower Columbia | 04Fall | Lower Columbia | 03_WCASFA | 2018 | 0.6002 | 447 | 331 |
| OtsPAHH_seg_ss18 | Pahsimeroi Fish Hatchery | Salmon River | Snake | 02Spring/Summer | Interior stream type | 16_UPSALM | 2018 | 0.9910 | 694 | 332 |
| OtsPARK_seg_sp18 | Parkdale Fish Facility | Hood River | Columbia | 01Spring | Lower Columbia | 02_WCASSP | 2018 | 0.7730 | 189 | 333 |
| OtsPRIE_seg_fa18 | Priest Rapids Hatchery | Mixed origins (Mid-/Upper Columbia Rivers) | Columbia | 04Fall | Interior ocean type | 18_UCOLSF | 2018 | 0.8617 | 6418 | 334 |
| OtsRAPH_seg_sp18 | Rapid River Fish Hatchery | Rapid River | Snake | 01Spring | Interior stream type | 12_HELLSC | 2018 | 0.8831 | 1729 | 335 |
| OtsRBFH_seg_sp18 | Round Butte Fish Hatchery | Deschutes River | Columbia | 01Spring | Interior stream type | 07_DESCSP | 2018 | 0.9603 | 684 | 336 |
| OtsSAWT_seg_ss18 | Sawtooth Fish Hatchery | Salmon River | Snake | 02Spring/Summer | Interior stream type | 16_UPSALM | 2018 | 1.0000 | 898 | 337 |
| OtsSSAN_seg_sp18 | South Santiam Hatchery |  | Willamette | 01Spring | Lower Columbia | 04_WILLAM | 2018 | 0.5355 | 805 | 338 |
| OtsSPEE_seg_sp18 | Speelyai Fish Hatchery |  | Lower Columbia | 01Spring | Lower Columbia | 02_WCASSP | 2018 | 0.8331 | 1203 | 339 |
| OtsTOUC_seg_sp18 | Little White Salmon National Fish Hatchery | #N/A | Columbia | 01Spring | Interior stream type | 11_TUCANO | 2018 | 0.9522 | 121 | 340 |
| OtsUMAT_seg_fa18 | Umatilla Fish Hatchery |  | Columbia | 04Fall | Interior ocean type | 23_UMATILLAFA | 2018 | 0.9573 | 952 | 341 |
| OtsUMAT_seg_sp18 | Umatilla Fish Hatchery |  | Columbia | 01Spring | Interior stream type | 21_UMATILLASP | 2018 | 0.9913 | 459 | 342 |
| OtsWSNF_seg_sp18 | Warm Springs National Fish Hatchery |  | Columbia | 01Spring | Interior stream type | 07_DESCSP | 2018 | 0.8985 | 291 | 343 |
| OtsWASH_seg_fa18 | Washougal Fish Hatchery |  | Lower Columbia | 04Fall | Lower Columbia | 03_WCASFA | 2018 | 0.5770 | 594 | 344 |
| OtsWELL_seg_su18 | Wells Fish Hatchery |  | Columbia | 03Summer | Interior ocean type | 18_UCOLSF | 2018 | 0.8209 | 752 | 345 |
| OtsWINT_seg_sp18 | Winthrop National Fish Hatchery |  | Columbia | 01Spring | Interior stream type | 10_UCOLSP | 2018 | 0.7993 | 346 | 346 |
| OtsPROS_seg_fa18 | Yakima Nation Prosser Hatchery |  | Columbia | 04Fall | Interior ocean type | 18_UCOLSF | 2018 | 0.8668 | 81 | 347 |
| OtsYRRD_int_sp18 | Yakima River Roza Dam | Integrated | Columbia | 01Spring | Interior stream type | 09_YAKIMA | 2018 | 0.9811 | 313 | 348 |
| OtsYRRD_seg_sp18 | Yakima River Roza Dam | Segregated | Columbia | 01Spring | Interior stream type | 09_YAKIMA | 2018 | 1.0000 | 102 | 349 |
| OtsRING_seg_fa18 | Ringold Springs State Hatchery |  | Columbia | 04Fall | Interior ocean type | 18_UCOLSF | 2018 | 0.8320 | 436 | 350 |
| OtsSPCR_seg_fa18 | Spring Creek National Fish Hatchery |  | Columbia | 04Fall | Lower Columbia | 05_SPCRTU | 2018 | 1.0000 | 6259 | 351 |
| OtsBIGC_seg_fa19 | Big Creek Hatchery |  | Lower Columbia | 04Fall | Lower Columbia | 03_WCASFA | 2019 | 0.8693 | 910 | 352 |
| OtsBONN_seg_fa19 | Bonneville Dam Hatchery |  | Columbia | 04Fall | Lower Columbia | 05_SPCRTU | 2019 | 0.5676 | 1337 | 353 |
| OtsCARS_seg_sp19 | Carson National Fish Hatchery | Mixed origins (Snake and Mid-/Upper-Columbia Rivers) | Columbia | 01Spring | Interior stream type | 20_BONPOOLSP | 2019 | 1.0000 | 824 | 354 |
| OtsCHJO_int_su19 | Chief Joseph Hatchery | Integrated | Columbia | 03Summer | Interior ocean type | 18_UCOLSF | 2019 | 0.9801 | 594 | 355 |
| OtsCHJO_seg_sp19 | Chief Joseph Hatchery | Spring | Columbia | 01Spring | Interior stream type | 10_UCOLSP | 2019 | 0.8227 | 478 | 356 |
| OtsCHJO_seg_su19 | Chief Joseph Hatchery | Segregated | Columbia | 03Summer | Interior ocean type | 18_UCOLSF | 2019 | 0.9623 | 464 | 357 |
| OtsCLWH_seg_sp19 | Clearwater Fish Hatchery | Clearwater River | Snake | 01Spring | Interior stream type | 12_HELLSC | 2019 | 0.9982 | 969 | 358 |
| OtsPOWP_seg_sp19 | Clearwater Fish Hatchery | Lochsa River (Powell Facility) | Snake | 01Spring | Interior stream type | 12_HELLSC | 2019 | 1.0000 | 81 | 359 |
| OtsCOWL_seg_fa19 | Cowlitz Salmon Hatchery |  | Lower Columbia | 04Fall | Lower Columbia | 03_WCASFA | 2019 | 0.9303 | 1088 | 360 |
| OtsCOWL_seg_sp19 | Cowlitz Salmon Hatchery |  | Lower Columbia | 01Spring | Lower Columbia | 02_WCASSP | 2019 | 0.8965 | 962 | 361 |
| OtsDWOR_seg_sp19 | Dworshak National Fish Hatchery | Clearwater River | Snake | 01Spring | Interior stream type | 12_HELLSC | 2019 | 0.9983 | 1207 | 362 |
| OtsKOOS_seg_sp19 | Dworshak National Fish Hatchery | Clearwater River | Snake | 01Spring | Interior stream type | 12_HELLSC | 2019 | 0.9373 | 667 | 363 |
| OtsEAST_seg_su19 | Eastbank Fish Hatchery | Chelan Falls, Methow River, Okanogan River, Wenatchee River | Columbia | 03Summer | Interior ocean type | 18_UCOLSF | 2019 | 1.0000 | 952 | 364 |
| OtsEAST_seg_sp19 | Eastbank Fish Hatchery | Chelan Falls, Methow River, Okanogan River, Wenatchee River | Columbia | 01Spring | Interior stream type | 10_UCOLSP | 2019 | 0.8925 | 188 | 365 |
| OtsENFH_seg_su19 | Entiat National Fish Hatchery | Entiat | Columbia | 03Summer | Interior ocean type | 18_UCOLSF | 2019 | 0.9365 | 300 | 366 |
| OtsKALA_seg_sp19 | Kalama Falls Hatchery |  | Lower Columbia | 01Spring | Lower Columbia | 02_WCASSP | 2019 | 0.8563 | 533 | 367 |
| OtsKALA_seg_fa19 | Kalama Falls Hatchery |  | Lower Columbia | 04Fall | Lower Columbia | 03_WCASFA | 2019 | 0.7950 | 2805 | 368 |
| OtsKLIC_seg_sp19 | Klickitat Hatchery | Klickitat River | Columbia | 01Spring | Interior stream type | 06_KLICKR | 2019 | 1.0000 | 93 | 369 |
| OtsKLIC_seg_fa19 | Klickitat Hatchery | Little White Salmon River | Columbia | 04Fall | Interior ocean type | 22_BONPOOLFA | 2019 | 0.5650 | 342 | 370 |
| OtsLNFH_seg_sp19 | Leavenworth National Fish Hatchery | Mixed origins (Upper-Columbia Rivers) | Columbia | 01Spring | Interior stream type | 10_UCOLSP | 2019 | 1.0000 | 663 | 371 |
| OtsLWSN_seg_sp19 | Little White Salmon National Fish Hatchery | Little White Salmon River | Columbia | 01Spring | Interior stream type | 20_BONPOOLSP | 2019 | 0.9528 | 1919 | 372 |
| OtsLWSN_seg_fa19 | Little White Salmon National Fish Hatchery | Little White Salmon River | Columbia | 04Fall | Interior ocean type | 22_BONPOOLFA | 2019 | 1.0000 | 6209 | 373 |
| OtsIMNW_seg_ss19 | Lookingglass Fish Hatchery | Imnaha River | Snake | 02Spring/Summer | Interior stream type | 12_HELLSC | 2019 | 0.9620 | 256 | 374 |
| OtsLOOK_seg_sp19 | Lookingglass Fish Hatchery | Lookingglass/Catherine Creek/Grande Ronde/Lostine River | Snake | 01Spring | Interior stream type | 12_HELLSC | 2019 | 0.9714 | 519 | 375 |
| OtsLYON_seg_fa19 | Lyons Ferry Fish Hatchery | Mixed origins (Snake River and Upper Columbia River) | Snake | 04Fall | Interior ocean type | 19_SRFALL | 2019 | 0.9597 | 1637 | 376 |
| OtsLYON_seg_sp19 | Lyons Ferry Fish Hatchery | Tucannon River | Snake | 01Spring | Interior stream type | 11_TUCANO | 2019 | 0.8653 | 80 | 377 |
| OtsJHNW_int_ss19 | McCall Fish Hatchery | Johnson Creek | Snake | 02Spring/Summer | Interior stream type | 13_SFSALM | 2019 | 1.0000 | 62 | 378 |
| OtsMCCA_seg_ss19 | McCall Fish Hatchery | South Fork Salmon | Snake | 02Spring/Summer | Interior stream type | 13_SFSALM | 2019 | 0.9748 | 1063 | 379 |
| OtsMETH_seg_sp19 | Methow Fish Hatchery | Upper Methow and Twisp Rivers | Columbia | 01Spring | Interior stream type | 10_UCOLSP | 2019 | 1.0000 | 118 | 380 |
| OtsNPFH_seg_fa19 | Nez Perce Tribal Fish Hatchery | Mixed origins (Snake River and Clearwater River) | Snake | 04Fall | Interior ocean type | 19_SRFALL | 2019 | 0.9711 | 742 | 381 |
| OtsNPFH_seg_sp19 | Nez Perce | Newsome Creek and Yoosa/Camp Creek | Snake | 01Spring | Interior stream type | 12_HELLSC | 2019 | 0.9528 | 403 | 382 |
| OtsNSAN_seg_sp19 | North Santiam Hatchery |  | Willamette | 01Spring | Lower Columbia | 04_WILLAM | 2019 | 0.5192 | 686 | 383 |
| OtsNTOU_seg_fa19 | North Toutle Hatchery |  | Lower Columbia | 04Fall | Lower Columbia | 03_WCASFA | 2019 | 0.0000 | 0 | 384 |
| OtsPAHH_seg_ss19 | Pahsimeroi Fish Hatchery | Salmon River | Snake | 02Spring/Summer | Interior stream type | 16_UPSALM | 2019 | 0.9959 | 456 | 385 |
| OtsPARK_seg_sp19 | Parkdale Fish Facility | Hood River | Columbia | 01Spring | Lower Columbia | 02_WCASSP | 2019 | 0.9797 | 194 | 386 |
| OtsPRIE_seg_fa19 | Priest Rapids Hatchery | Mixed origins (Mid-/Upper Columbia Rivers) | Columbia | 04Fall | Interior ocean type | 18_UCOLSF | 2019 | 0.9615 | 7874 | 387 |
| OtsRAPH_seg_sp19 | Rapid River Fish Hatchery | Rapid River | Snake | 01Spring | Interior stream type | 12_HELLSC | 2019 | 0.9515 | 2694 | 388 |
| OtsRBFH_seg_sp19 | Round Butte Fish Hatchery | Deschutes River | Columbia | 01Spring | Interior stream type | 07_DESCSP | 2019 | 0.9042 | 310 | 389 |
| OtsSAWT_seg_ss19 | Sawtooth Fish Hatchery | Salmon River | Snake | 02Spring/Summer | Interior stream type | 16_UPSALM | 2019 | 0.9698 | 659 | 390 |
| OtsSSAN_seg_sp19 | South Santiam Hatchery |  | Willamette | 01Spring | Lower Columbia | 04_WILLAM | 2019 | 0.7213 | 620 | 391 |
| OtsSPEE_seg_sp19 | Speelyai Fish Hatchery |  | Lower Columbia | 01Spring | Lower Columbia | 02_WCASSP | 2019 | 0.6836 | 778 | 392 |
| OtsTOUC_seg_sp19 | Little White Salmon National Fish Hatchery | #N/A | Columbia | 01Spring | Interior stream type | 11_TUCANO | 2019 | 1.0000 | 114 | 393 |
| OtsUMAT_seg_fa19 | Umatilla Fish Hatchery |  | Columbia | 04Fall | Interior ocean type | 23_UMATILLAFA | 2019 | 1.0000 | 179 | 394 |
| OtsUMAT_seg_sp19 | Umatilla Fish Hatchery |  | Columbia | 01Spring | Interior stream type | 21_UMATILLASP | 2019 | 1.0000 | 493 | 395 |
| OtsWSNF_seg_sp19 | Warm Springs National Fish Hatchery |  | Columbia | 01Spring | Interior stream type | 07_DESCSP | 2019 | 0.8849 | 238 | 396 |
| OtsWASH_seg_fa19 | Washougal Fish Hatchery |  | Lower Columbia | 04Fall | Lower Columbia | 03_WCASFA | 2019 | 0.4767 | 1151 | 397 |
| OtsWELL_seg_su19 | Wells Fish Hatchery |  | Columbia | 03Summer | Interior ocean type | 18_UCOLSF | 2019 | 1.0000 | 980 | 398 |
| OtsWINT_seg_sp19 | Winthrop National Fish Hatchery |  | Columbia | 01Spring | Interior stream type | 10_UCOLSP | 2019 | 0.9890 | 361 | 399 |
| OtsPROS_seg_fa19 | Yakima Nation Prosser Hatchery |  | Columbia | 04Fall | Interior ocean type | 18_UCOLSF | 2019 | 0.4147 | 85 | 400 |
| OtsYRRD_int_sp19 | Yakima River Roza Dam | Integrated | Columbia | 01Spring | Interior stream type | 09_YAKIMA | 2019 | 0.9936 | 311 | 401 |
| OtsYRRD_seg_sp19 | Yakima River Roza Dam | Segregated | Columbia | 01Spring | Interior stream type | 09_YAKIMA | 2019 | 1.0000 | 127 | 402 |
| OtsRING_seg_fa19 | Ringold Springs State Hatchery |  | Columbia | 04Fall | Interior ocean type | 18_UCOLSF | 2019 | 0.9602 | 830 | 403 |
| OtsSPCR_seg_fa19 | Spring Creek National Fish Hatchery |  | Columbia | 04Fall | Lower Columbia | 05_SPCRTU | 2019 | 0.9068 | 4927 | 404 |
| OtsCHJO_int_su20 | Chief Joseph Hatchery | Integrated | Columbia | 03Summer | Interior ocean type | 18_UCOLSF | 2020 | 0.6723 | 419 | 405 |
| OtsCHJO_seg_su20 | Chief Joseph Hatchery | Segregated | Columbia | 03Summer | Interior ocean type | 18_UCOLSF | 2020 | 0.8550 | 454 | 406 |
| OtsEAST_seg_su20 | Eastbank Fish Hatchery | Chelan Falls, Methow River, Okanogan River, Wenatchee River | Columbia | 03Summer | Interior ocean type | 18_UCOLSF | 2020 | 1.0000 | 744 | 407 |
| OtsENFH_seg_su20 | Entiat National Fish Hatchery | Entiat | Columbia | 03Summer | Interior ocean type | 18_UCOLSF | 2020 | 0.9088 | 286 | 408 |
| OtsPRIE_seg_fa20 | Priest Rapids Hatchery | Mixed origins (Mid-/Upper Columbia Rivers) | Columbia | 04Fall | Interior ocean type | 18_UCOLSF | 2020 | 0.7450 | 5291 | 409 |
| OtsWELL_seg_su20 | Wells Fish Hatchery |  | Columbia | 03Summer | Interior ocean type | 18_UCOLSF | 2020 | 0.9716 | 964 | 410 |
| OtsPROS_seg_fa20 | Yakima Nation Prosser Hatchery |  | Columbia | 04Fall | Interior ocean type | 18_UCOLSF | 2020 | 0.9517 | 719 | 411 |
| OtsRING_seg_fa20 | Ringold Springs State Hatchery |  | Columbia | 04Fall | Interior ocean type | 18_UCOLSF | 2020 | 0.9201 | 776 | 412 |
| OtsKLIC_seg_fa20 | Klickitat Hatchery | Klickitat River | Columbia | 04Fall | Interior ocean type | 22_BONPOOLFA | 2020 | 0.0000 | 0 | 413 |
| OtsLWSN_seg_fa20 | Little White Salmon National Fish Hatchery | Little White Salmon River | Columbia | 04Fall | Interior ocean type | 22_BONPOOLFA | 2020 | 0.9945 | 4310 | 414 |
| OtsUMAT_seg_fa20 | Umatilla Fish Hatchery |  | Columbia | 04Fall | Interior ocean type | 23_UMATILLAFA | 2020 | 0.8908 | 655 | 415 |
| OtsKLIC_seg_sp20 | Klickitat Hatchery | Klickitat River | Columbia | 01Spring | Interior stream type | 06_KLICKR | 2020 | 0.9830 | 464 | 416 |
| OtsYRRD_int_sp20 | Yakima River Roza Dam | Integrated | Columbia | 01Spring | Interior stream type | 09_YAKIMA | 2020 | 0.9907 | 425 | 417 |
| OtsYRRD_seg_sp20 | Yakima River Roza Dam | Segregated | Columbia | 01Spring | Interior stream type | 09_YAKIMA | 2020 | 1.0000 | 133 | 418 |
| OtsCHJO_seg_sp20 | Chief Joseph Hatchery | Spring | Columbia | 01Spring | Interior stream type | 10_UCOLSP | 2020 | 0.9489 | 527 | 419 |
| OtsEAST_seg_sp20 | Eastbank Fish Hatchery | Chelan Falls, Methow River, Okanogan River, Wenatchee River | Columbia | 01Spring | Interior stream type | 10_UCOLSP | 2020 | 1.0000 | 202 | 420 |
| OtsLNFH_seg_sp20 | Leavenworth National Fish Hatchery | Mixed origins (Upper-Columbia Rivers) | Columbia | 01Spring | Interior stream type | 10_UCOLSP | 2020 | 0.9393 | 974 | 421 |
| OtsMETH_seg_sp20 | Methow Fish Hatchery | Upper Methow and Twisp Rivers | Columbia | 01Spring | Interior stream type | 10_UCOLSP | 2020 | 0.3920 | 72 | 422 |
| OtsWINT_seg_sp20 | Winthrop National Fish Hatchery |  | Columbia | 01Spring | Interior stream type | 10_UCOLSP | 2020 | 0.9587 | 375 | 423 |
| OtsTOUC_seg_sp20 | Little White Salmon National Fish Hatchery | #N/A | Columbia | 01Spring | Interior stream type | 11_TUCANO | 2020 | 0.0000 | 0 | 424 |
| OtsCARS_seg_sp20 | Carson National Fish Hatchery | Mixed origins (Snake and Mid-/Upper-Columbia Rivers) | Columbia | 01Spring | Interior stream type | 20_BONPOOLSP | 2020 | 1.0000 | 696 | 425 |
| OtsLWSN_seg_sp20 | Little White Salmon National Fish Hatchery | Little White Salmon River | Columbia | 01Spring | Interior stream type | 20_BONPOOLSP | 2020 | 0.9509 | 1452 | 426 |
| OtsUMAT_seg_sp20 | Umatilla Fish Hatchery |  | Columbia | 01Spring | Interior stream type | 21_UMATILLASP | 2020 | 1.0241 | 423 | 427 |
| OtsPARK_seg_sp20 | Parkdale Fish Facility | Hood River | Columbia | 01Spring | Lower Columbia | 02_WCASSP | 2020 | 1.0000 | 259 | 428 |
| OtsBONN_seg_fa20 | Bonneville Dam Hatchery |  | Columbia | 04Fall | Lower Columbia | 05_SPCRTU | 2020 | 0.9922 | 1777 | 429 |
| OtsSPCR_seg_fa20 | Spring Creek National Fish Hatchery |  | Columbia | 04Fall | Lower Columbia | 05_SPCRTU | 2020 | 1.0000 | 5450 | 430 |
| OtsBIGC_seg_fa20 | Big Creek Hatchery |  | Lower Columbia | 04Fall | Lower Columbia | 03_WCASFA | 2020 | 0.9730 | 218 | 431 |
| OtsJHNW_int_ss20 | McCall Fish Hatchery | Johnson Creek | Snake | 02Spring/Summer | Interior stream type | 13_SFSALM | 2020 | 0.9565 | 90 | 432 |
| OtsMCKE_seg_sp20 | McKenzie Hatchery |  | Willamette | 01Spring | Lower Columbia | 04_WILLAM | 2020 | 0.9375 | 457 | 433 |
| OtsNSAN_seg_sp20 | North Santiam Hatchery |  | Willamette | 01Spring | Lower Columbia | 04_WILLAM | 2020 | 0.0000 | 0 | 434 |
| OtsSSAN_seg_sp20 | South Santiam Hatchery |  | Willamette | 01Spring | Lower Columbia | 04_WILLAM | 2020 | 0.4976 | 522 | 435 |
| OtsRBFH_seg_sp20 | Round Butte Fish Hatchery | Deschutes River | Columbia | 01Spring | Interior stream type | 07_DESCSP | 2020 | 0.9809 | 206 | 436 |
| OtsWSNF_seg_sp20 | Warm Springs National Fish Hatchery |  | Columbia | 01Spring | Interior stream type | 07_DESCSP | 2020 | 0.9590 | 331 | 437 |
| OtsCOWL_seg_sp20 | Cowlitz Salmon Hatchery |  | Lower Columbia | 01Spring | Lower Columbia | 02_WCASSP | 2020 | 0.7656 | 336 | 438 |
| OtsKALA_seg_sp20 | Kalama Falls Hatchery |  | Lower Columbia | 01Spring | Lower Columbia | 02_WCASSP | 2020 | 0.9264 | 616 | 439 |
| OtsSPEE_seg_sp20 | Speelyai Fish Hatchery |  | Lower Columbia | 01Spring | Lower Columbia | 02_WCASSP | 2020 | 0.7277 | 987 | 440 |
| OtsCOWL_seg_fa20 | Cowlitz Salmon Hatchery |  | Lower Columbia | 04Fall | Lower Columbia | 03_WCASFA | 2020 | 0.7067 | 1773 | 441 |
| OtsKALA_seg_fa20 | Kalama Falls Hatchery |  | Lower Columbia | 04Fall | Lower Columbia | 03_WCASFA | 2020 | 0.8621 | 3311 | 442 |
| OtsNTOU_seg_fa20 | North Toutle Hatchery |  | Lower Columbia | 04Fall | Lower Columbia | 03_WCASFA | 2020 | 0.0000 | 0 | 443 |
| OtsWASH_seg_fa20 | Washougal Fish Hatchery |  | Lower Columbia | 04Fall | Lower Columbia | 03_WCASFA | 2020 | 0.7237 | 923 | 444 |
| OtsLYON_seg_fa20 | Lyons Ferry Fish Hatchery | Mixed origins (Snake River and Upper Columbia River) | Snake | 04Fall | Interior ocean type | 19_SRFALL | 2020 | 0.9896 | 1719 | 445 |
| OtsNPFH_seg_fa20 | Nez Perce Tribal Fish Hatchery | Mixed origins (Snake River and Clearwater River) | Snake | 04Fall | Interior ocean type | 19_SRFALL | 2020 | 0.9632 | 687 | 446 |
| OtsLYON_seg_sp20 | Lyons Ferry Fish Hatchery | Tucannon River | Snake | 01Spring | Interior stream type | 11_TUCANO | 2020 | 1.0000 | 43 | 447 |
| OtsCLWH_seg_sp20 | Clearwater Fish Hatchery | Clearwater River | Snake | 01Spring | Interior stream type | 12_HELLSC | 2020 | 0.9951 | 1255 | 448 |
| OtsPOWP_seg_sp20 | Clearwater Fish Hatchery | Lochsa River (Powell Facility) | Snake | 01Spring | Interior stream type | 12_HELLSC | 2020 | 1.0000 | 147 | 449 |
| OtsDWOR_seg_sp20 | Dworshak National Fish Hatchery | Clearwater River | Snake | 01Spring | Interior stream type | 12_HELLSC | 2020 | 0.9131 | 1463 | 450 |
| OtsKOOS_seg_sp20 | Dworshak National Fish Hatchery | Clearwater River | Snake | 01Spring | Interior stream type | 12_HELLSC | 2020 | 0.9992 | 569 | 451 |
| OtsIMNW_seg_ss20 | Lookingglass Fish Hatchery | Imnaha River | Snake | 02Spring/Summer | Interior stream type | 12_HELLSC | 2020 | 0.7111 | 255 | 452 |
| OtsLOOK_seg_sp20 | Lookingglass Fish Hatchery | Lookingglass/Catherine Creek/Grande Ronde/Lostine River | Snake | 01Spring | Interior stream type | 12_HELLSC | 2020 | 0.8314 | 531 | 453 |
| OtsNPFH_seg_sp20 | Nez Perce | Newsome Creek and Yoosa/Camp Creek | Snake | 01Spring | Interior stream type | 12_HELLSC | 2020 | 0.9940 | 278 | 454 |
| OtsRAPH_seg_sp20 | Rapid River Fish Hatchery | Rapid River | Snake | 01Spring | Interior stream type | 12_HELLSC | 2020 | 0.9827 | 2909 | 455 |
| OtsMCCA_seg_ss20 | McCall Fish Hatchery | South Fork Salmon | Snake | 02Spring/Summer | Interior stream type | 13_SFSALM | 2020 | 0.9886 | 427 | 456 |
| OtsPAHH_seg_ss20 | Pahsimeroi Fish Hatchery | Salmon River | Snake | 02Spring/Summer | Interior stream type | 16_UPSALM | 2020 | 0.9864 | 303 | 457 |
| OtsSAWT_seg_ss20 | Sawtooth Fish Hatchery | Salmon River | Snake | 02Spring/Summer | Interior stream type | 16_UPSALM | 2020 | 0.9904 | 776 | 458 |
| OtsCHJO_int_su21 | Chief Joseph Hatchery | Integrated | Columbia | 03Summer | Interior ocean type | 18_UCOLSF | 2021 | 1.0000 | 569 | 459 |
| OtsCHJO_seg_su21 | Chief Joseph Hatchery | Segregated | Columbia | 03Summer | Interior ocean type | 18_UCOLSF | 2021 | 0.9350 | 468 | 460 |
| OtsEAST_seg_su21 | Eastbank Fish Hatchery | Chelan Falls, Methow River, Okanogan River, Wenatchee River | Columbia | 03Summer | Interior ocean type | 18_UCOLSF | 2021 | 0.8837 | 674 | 461 |
| OtsENFH_seg_su21 | Entiat National Fish Hatchery | Entiat | Columbia | 03Summer | Interior ocean type | 18_UCOLSF | 2021 | 0.8882 | 311 | 462 |
| OtsPRIE_seg_fa21 | Priest Rapids Hatchery | Mixed origins (Mid-/Upper Columbia Rivers) | Columbia | 04Fall | Interior ocean type | 18_UCOLSF | 2021 | 0.6730 | 5206 | 463 |
| OtsWELL_seg_su21 | Wells Fish Hatchery |  | Columbia | 03Summer | Interior ocean type | 18_UCOLSF | 2021 | 0.9045 | 913 | 464 |
| OtsPROS_seg_fa21 | Yakima Nation Prosser Hatchery |  | Columbia | 04Fall | Interior ocean type | 18_UCOLSF | 2021 | 0.9056 | 118 | 465 |
| OtsRING_seg_fa21 | Ringold Springs State Hatchery |  | Columbia | 04Fall | Interior ocean type | 18_UCOLSF | 2021 | 0.7621 | 756 | 466 |
| OtsKLIC_seg_fa21 | Klickitat Hatchery | Klickitat River | Columbia | 04Fall | Interior ocean type | 22_BONPOOLFA | 2021 | 0.0000 | 0 | 467 |
| OtsLWSN_seg_fa21 | Little White Salmon National Fish Hatchery | Little White Salmon River | Columbia | 04Fall | Interior ocean type | 22_BONPOOLFA | 2021 | 0.9887 | 8262 | 468 |
| OtsUMAT_seg_fa21 | Umatilla Fish Hatchery |  | Columbia | 04Fall | Interior ocean type | 23_UMATILLAFA | 2021 | 0.7975 | 434 | 469 |
| OtsKLIC_seg_sp21 | Klickitat Hatchery | Klickitat River | Columbia | 01Spring | Interior stream type | 06_KLICKR | 2021 | 0.9086 | 509 | 470 |
| OtsYRRD_int_sp21 | Yakima River Roza Dam | Integrated | Columbia | 01Spring | Interior stream type | 09_YAKIMA | 2021 | 0.9640 | 433 | 471 |
| OtsYRRD_seg_sp21 | Yakima River Roza Dam | Segregated | Columbia | 01Spring | Interior stream type | 09_YAKIMA | 2021 | 1.0000 | 113 | 472 |
| OtsCHJO_seg_sp21 | Chief Joseph Hatchery | Spring | Columbia | 01Spring | Interior stream type | 10_UCOLSP | 2021 | 0.9883 | 509 | 473 |
| OtsEAST_seg_sp21 | Eastbank Fish Hatchery | Chelan Falls, Methow River, Okanogan River, Wenatchee River | Columbia | 01Spring | Interior stream type | 10_UCOLSP | 2021 | 0.8909 | 185 | 474 |
| OtsLNFH_seg_sp21 | Leavenworth National Fish Hatchery | Mixed origins (Upper-Columbia Rivers) | Columbia | 01Spring | Interior stream type | 10_UCOLSP | 2021 | 0.8910 | 926 | 475 |
| OtsMETH_seg_sp21 | Methow Fish Hatchery | Upper Methow and Twisp Rivers | Columbia | 01Spring | Interior stream type | 10_UCOLSP | 2021 | 0.9137 | 65 | 476 |
| OtsWINT_seg_sp21 | Winthrop National Fish Hatchery |  | Columbia | 01Spring | Interior stream type | 10_UCOLSP | 2021 | 1.0000 | 424 | 477 |
| OtsTOUC_seg_sp21 | Little White Salmon National Fish Hatchery | #N/A | Columbia | 01Spring | Interior stream type | 11_TUCANO | 2021 | 0.0000 | 0 | 478 |
| OtsCARS_seg_sp21 | Carson National Fish Hatchery | Mixed origins (Snake and Mid-/Upper-Columbia Rivers) | Columbia | 01Spring | Interior stream type | 20_BONPOOLSP | 2021 | 1.0000 | 1331 | 479 |
| OtsLWSN_seg_sp21 | Little White Salmon National Fish Hatchery | Little White Salmon River | Columbia | 01Spring | Interior stream type | 20_BONPOOLSP | 2021 | 0.9454 | 1089 | 480 |
| OtsUMAT_seg_sp21 | Umatilla Fish Hatchery |  | Columbia | 01Spring | Interior stream type | 21_UMATILLASP | 2021 | 0.9555 | 478 | 481 |
| OtsPARK_seg_sp21 | Parkdale Fish Facility | Hood River | Columbia | 01Spring | Lower Columbia | 02_WCASSP | 2021 | 1.0000 | 180 | 482 |
| OtsBONN_seg_fa21 | Bonneville Dam Hatchery |  | Columbia | 04Fall | Lower Columbia | 05_SPCRTU | 2021 | 0.0000 | 0 | 483 |
| OtsSPCR_seg_fa21 | Spring Creek National Fish Hatchery |  | Columbia | 04Fall | Lower Columbia | 05_SPCRTU | 2021 | 0.9216 | 6048 | 484 |
| OtsBIGC_seg_fa21 | Big Creek Hatchery |  | Lower Columbia | 04Fall | Lower Columbia | 03_WCASFA | 2021 | 0.0000 | 0 | 485 |
| OtsJHNW_int_ss21 | McCall Fish Hatchery | Johnson Creek | Snake | 02Spring/Summer | Interior stream type | 13_SFSALM | 2021 | 0.0000 | 0 | 486 |
| OtsMCKE_seg_sp21 | McKenzie Hatchery |  | Willamette | 01Spring | Lower Columbia | 04_WILLAM | 2021 | 0.0000 | 0 | 487 |
| OtsNSAN_seg_sp21 | North Santiam Hatchery |  | Willamette | 01Spring | Lower Columbia | 04_WILLAM | 2021 | 0.8996 | 736 | 488 |
| OtsSSAN_seg_sp21 | South Santiam Hatchery |  | Willamette | 01Spring | Lower Columbia | 04_WILLAM | 2021 | 0.5665 | 560 | 489 |
| OtsRBFH_seg_sp21 | Round Butte Fish Hatchery | Deschutes River | Columbia | 01Spring | Interior stream type | 07_DESCSP | 2021 | 1.0000 | 202 | 490 |
| OtsWSNF_seg_sp21 | Warm Springs National Fish Hatchery |  | Columbia | 01Spring | Interior stream type | 07_DESCSP | 2021 | 0.4724 | 211 | 491 |
| OtsCOWL_seg_sp21 | Cowlitz Salmon Hatchery |  | Lower Columbia | 01Spring | Lower Columbia | 02_WCASSP | 2021 | 0.8261 | 1197 | 492 |
| OtsKALA_seg_sp21 | Kalama Falls Hatchery |  | Lower Columbia | 01Spring | Lower Columbia | 02_WCASSP | 2021 | 0.8417 | 767 | 493 |
| OtsSPEE_seg_sp21 | Speelyai Fish Hatchery |  | Lower Columbia | 01Spring | Lower Columbia | 02_WCASSP | 2021 | 0.6632 | 965 | 494 |
| OtsCOWL_seg_fa21 | Cowlitz Salmon Hatchery |  | Lower Columbia | 04Fall | Lower Columbia | 03_WCASFA | 2021 | 0.8054 | 1768 | 495 |
| OtsKALA_seg_fa21 | Kalama Falls Hatchery |  | Lower Columbia | 04Fall | Lower Columbia | 03_WCASFA | 2021 | 0.8597 | 2586 | 496 |
| OtsNTOU_seg_fa21 | North Toutle Hatchery |  | Lower Columbia | 04Fall | Lower Columbia | 03_WCASFA | 2021 | 0.9029 | 248 | 497 |
| OtsWASH_seg_fa21 | Washougal Fish Hatchery |  | Lower Columbia | 04Fall | Lower Columbia | 03_WCASFA | 2021 | 0.6484 | 612 | 498 |
| OtsLYON_seg_fa21 | Lyons Ferry Fish Hatchery | Mixed origins (Snake River and Upper Columbia River) | Snake | 04Fall | Interior ocean type | 19_SRFALL | 2021 | 0.9814 | 1798 | 499 |
| OtsNPFH_seg_fa21 | Nez Perce Tribal Fish Hatchery | Mixed origins (Snake River and Clearwater River) | Snake | 04Fall | Interior ocean type | 19_SRFALL | 2021 | 0.9826 | 685 | 500 |

Note: Broodstock is an abbreviation to designate species (“Ots”, *Onchorynchus tswawystcha*), the spawning hatchery (four letter code), hatchery programs that integrate natural-origin fish (“int”) vs. segregated (“seg”) lines, stock description if the hatcheries maintain multiple programs, river basin, run type (four categories according to which management period the stock typically passes Bonneville Dam), genetic lineage, expected GSI reporting group (“GenStock_exp”), brood year (“BY”), PBT tag “Rate”, number of samples (“N”), and 3-digit code used for Figure labels.

| Spring VSI stock | GenStock | GenStock name | Run | Lineage | Prop. Correct | Label |
| --- | --- | --- | --- | --- | --- | --- |
| lower | 01_YOUNGS | Youngs Bay | Spring | Lower Columbia | 1.0000 | A |
| lower | 02_WCASSP | West Cascade Spring | Spring | Lower Columbia | 0.8190 | B |
|  | 03_WCASFA | West Cascade Fall | Fall | Lower Columbia | 0.9509 | C |
| lower | 04_WILLAM | Willamette | Spring | Lower Columbia | 1.0000 | D |
|  | 05_SPCRTU | Spring Creek Tule | Fall | Lower Columbia | 0.9302 | E |
| upriver | 06_KLICKR | Klickitat | Spring | Interior stream type | 0.9714 | F |
| upriver | 07_DESCSP | Deschutes spring | Spring | Interior stream type | 0.9040 | G |
| upriver | 08_JOHNDR | John Day | Spring | Interior stream type | 0.6289 | H |
| upriver | 09_YAKIMA | Yakima | Spring | Interior stream type | 1.0000 | I |
| upriver | 10_UCOLSP | Upper Columbia spring | Spring | Interior stream type | 0.7234 | J |
| upriver | 11_TUCANO | Tucannon | Spring | Interior stream type | 0.7568 | K |
| upriver | 12_HELLSC | Hells Canyon | Spring/Summer | Interior stream type | 0.7411 | L |
| upriver | 13_SFSALM | South Fork Salmon | Spring/Summer | Interior stream type | 0.6907 | M |
| upriver | 14_CHMBLN | Chamberlain Creek | Spring/Summer | Interior stream type | 0.8614 | N |
| upriver | 15_MFSALM | Middle Fork Salmon | Spring/Summer | Interior stream type | 0.8701 | O |
| upriver | 16_UPSALM | Upper Salmon | Spring/Summer | Interior stream type | 0.8668 | P |
|  | 17_DESCFA | Deschutes fall | Fall | Interior ocean type | 0.9196 | Q |
|  | 18_UCOLSF | Upper Columbia summer/fall | Summer/Fall | Interior ocean type | 0.8732 | R |
|  | 19_SRFALL | Snake River fall | Fall | Interior ocean type | 0.6842 | S |
| upriver | 20_BONPOOLSP* | Bonneville Pool spring | Spring | Interior stream type | - | T |
| upriver | 21_UMATILLASP* | Umatilla spring | Spring | Interior stream type | - | U |
|  | 22_BONPOOLFA* | Bonneville Pool fall | Fall | Interior ocean type | - | V |
|  | 23_UMATILLAFA* | Umatilla fall | Fall | Interior ocean type | - | W |
|  | Average |  |  |  | 0.8522 |  |

Table S3. Reporting group (GenStock) definitions for the GSI baseline.

Note: This GSI baseline has been shown to provide an average of 85% correct assignment (“Prop. Correct”) to reporting groups using leave-one-out analysis (Hasselman et al. 2017). * indicates four GenStocks that are only used with fish that have been PBT assigned to hatcheries categorized in these GenStock units; GSI assignments are only observed to the 19 remaining stocks. “Label” indicates the letter code used for each stock in the Figures.

Table S4. Hatchery broodstock assignments in the Spring Chinook Salmon Test Fishery (2017 – 2022).

|  |  | 2017 | | | | | 2018 | | | | | 2019 | | | | | 2021 | | | | | 2022 | | | | | Grand Total | | | | |
| --- | --- | --- | --- | --- | --- | --- | --- | --- | --- | --- | --- | --- | --- | --- | --- | --- | --- | --- | --- | --- | --- | --- | --- | --- | --- | --- | --- | --- | --- | --- | --- |
| Gen Stock |  | Lower | | Upriver | |  | Lower | | Upriver | |  | Lower | | Upriver | |  | Lower | | Upriver | |  | Lower | | Upriver | |  | Lower | | Upriver | |  |
| Exp | Hatchery broodstock | H | HNC | H | HNC | Total | H | HNC | H | HNC | Total | H | HNC | H | HNC | Total | H | HNC | H | HNC | Total | H | HNC | H | HNC | Total | H | HNC | H | HNC | Total |
| 02_WCASSP | OtsCOWL_seg_sp15 |  |  |  |  | 0 |  |  |  |  | 0 | 1 |  |  |  | 1 |  |  |  |  | 0 |  |  |  |  | 0 | 1 | 0 | 0 | 0 | 1 |
|  | OtsCOWL_seg_sp17 |  |  |  |  | 0 |  |  |  |  | 0 |  |  |  |  | 0 | 1 |  |  |  | 1 |  |  |  |  | 0 | 1 | 0 | 0 | 0 | 1 |
|  | OtsCOWL_seg_sp18 |  |  |  |  | 0 |  |  |  |  | 0 |  |  |  |  | 0 |  |  |  |  | 0 | 4 |  | 1 |  | 5 | 4 | 0 | 1 | 0 | 5 |
|  | OtsKALA_seg_sp15 |  |  |  |  | 0 |  |  |  |  | 0 | 2 |  |  |  | 2 |  |  |  |  | 0 |  |  |  |  | 0 | 2 | 0 | 0 | 0 | 2 |
|  | OtsKALA_seg_sp16 |  |  |  |  | 0 |  |  |  |  | 0 |  |  |  |  | 0 | 1 |  |  |  | 1 |  |  |  |  | 0 | 1 | 0 | 0 | 0 | 1 |
|  | OtsKALA_seg_sp17 |  |  |  |  | 0 |  |  |  |  | 0 |  |  |  |  | 0 |  |  |  |  | 0 | 1 |  |  |  | 1 | 1 | 0 | 0 | 0 | 1 |
|  | OtsKALA_seg_sp18 |  |  |  |  | 0 |  |  |  |  | 0 |  |  |  |  | 0 |  |  |  |  | 0 | 1 |  | 1 |  | 2 | 1 | 0 | 1 | 0 | 2 |
|  | OtsLEWI_seg_sp17 |  |  |  |  | 0 |  |  |  |  | 0 |  |  |  |  | 0 |  |  | 1 |  | 1 |  |  |  |  | 0 | 0 | 0 | 1 | 0 | 1 |
|  | OtsPARK_seg_sp13 |  |  | 1 |  | 1 |  |  |  |  | 0 |  |  |  |  | 0 |  |  |  |  | 0 |  |  |  |  | 0 | 0 | 0 | 1 | 0 | 1 |
|  | OtsPARK_seg_sp14 |  |  |  |  | 0 |  |  | 2 |  | 2 |  |  |  |  | 0 |  |  |  |  | 0 |  |  |  |  | 0 | 0 | 0 | 2 | 0 | 2 |
|  | OtsPARK_seg_sp15 |  |  |  |  | 0 |  |  |  |  | 0 | 1 |  | 2 |  | 3 |  |  |  |  | 0 |  |  |  |  | 0 | 1 | 0 | 2 | 0 | 3 |
|  | OtsPARK_seg_sp18 |  |  |  |  | 0 |  |  |  |  | 0 |  |  |  |  | 0 |  |  |  |  | 0 |  |  | 4 |  | 4 | 0 | 0 | 4 | 0 | 4 |
|  | OtsSPEE_seg_sp17 |  |  |  |  | 0 |  |  |  |  | 0 |  |  |  |  | 0 |  |  |  |  | 0 | 1 |  |  |  | 1 | 1 | 0 | 0 | 0 | 1 |
|  | OtsSPEE_seg_sp18 |  |  |  |  | 0 |  |  |  |  | 0 |  |  |  |  | 0 |  |  |  |  | 0 | 5 |  | 2 |  | 7 | 5 | 0 | 2 | 0 | 7 |
| 04_WILLAM | OtsNSAN_seg_sp18 |  |  |  |  | 0 |  |  |  |  | 0 |  |  |  |  | 0 |  |  |  |  | 0 | 7 |  |  |  | 7 | 7 | 0 | 0 | 0 | 7 |
|  | OtsSSAN_seg_sp15 |  |  |  |  | 0 |  |  |  |  | 0 | 1 |  | 1 |  | 2 |  |  |  |  | 0 |  |  |  |  | 0 | 1 | 0 | 1 | 0 | 2 |
|  | OtsSSAN_seg_sp18 |  |  |  |  | 0 |  |  |  |  | 0 |  |  |  |  | 0 |  |  |  |  | 0 | 4 |  |  |  | 4 | 4 | 0 | 0 | 0 | 4 |
| 06_KLICKR | OtsKLIC_seg_sp14 |  |  |  |  | 0 |  |  | 1 |  | 1 | 1 |  |  |  | 1 |  |  |  |  | 0 |  |  |  |  | 0 | 1 | 0 | 1 | 0 | 2 |
|  | OtsKLIC_seg_sp17 |  |  |  |  | 0 |  |  |  |  | 0 |  |  |  |  | 0 |  |  | 1 |  | 1 |  |  |  |  | 0 | 0 | 0 | 1 | 0 | 1 |
|  | OtsKLIC_seg_sp18 |  |  |  |  | 0 |  |  |  |  | 0 |  |  |  |  | 0 |  |  |  |  | 0 |  |  | 4 |  | 4 | 0 | 0 | 4 | 0 | 4 |
| 07_DESCSP | OtsRBFH_seg_sp13 |  |  | 1 |  | 1 |  |  |  |  | 0 |  |  |  |  | 0 |  |  |  |  | 0 |  |  |  |  | 0 | 0 | 0 | 1 | 0 | 1 |
|  | OtsRBFH_seg_sp14 |  |  |  |  | 0 | 3 |  | 2 |  | 5 |  |  |  |  | 0 |  |  |  |  | 0 |  |  |  |  | 0 | 3 | 0 | 2 | 0 | 5 |
|  | OtsRBFH_seg_sp15 |  |  |  |  | 0 |  |  |  |  | 0 |  |  | 2 |  | 2 |  |  |  |  | 0 |  |  |  |  | 0 | 0 | 0 | 2 | 0 | 2 |
|  | OtsRBFH_seg_sp17 |  |  |  |  | 0 |  |  |  |  | 0 |  |  |  |  | 0 |  |  |  | 1 | 1 |  |  |  |  | 0 | 0 | 0 | 0 | 1 | 1 |
|  | OtsRBFH_seg_sp18 |  |  |  |  | 0 |  |  |  |  | 0 |  |  |  |  | 0 |  |  |  |  | 0 |  |  | 3 |  | 3 | 0 | 0 | 3 | 0 | 3 |
|  | OtsWSNF_seg_sp15 |  |  |  |  | 0 |  |  |  |  | 0 |  |  | 1 |  | 1 |  |  |  |  | 0 |  |  |  |  | 0 | 0 | 0 | 1 | 0 | 1 |
|  | OtsWSNF_seg_sp17 |  |  |  |  | 0 |  |  |  |  | 0 |  |  |  |  | 0 | 1 |  | 3 |  | 4 |  |  |  |  | 0 | 1 | 0 | 3 | 0 | 4 |
|  | OtsWSNF_seg_sp18 |  |  |  |  | 0 |  |  |  |  | 0 |  |  |  |  | 0 |  |  |  |  | 0 |  |  | 2 |  | 2 | 0 | 0 | 2 | 0 | 2 |
| 09_YAKIMA | OtsYRRD_int_sp14 |  |  |  |  | 0 |  |  | 3 |  | 3 |  |  | 1 |  | 1 |  |  |  |  | 0 |  |  |  |  | 0 | 0 | 0 | 4 | 0 | 4 |
|  | OtsYRRD_int_sp15 |  |  |  |  | 0 |  |  |  |  | 0 | 2 |  | 8 |  | 10 |  |  |  |  | 0 |  |  |  |  | 0 | 2 | 0 | 8 | 0 | 10 |
|  | OtsYRRD_int_sp18 |  |  |  |  | 0 |  |  |  |  | 0 |  |  |  |  | 0 |  |  |  |  | 0 | 1 |  | 4 |  | 5 | 1 | 0 | 4 | 0 | 5 |
|  | OtsYRRD_seg_sp14 |  |  |  |  | 0 |  |  | 1 |  | 1 |  |  |  |  | 0 |  |  |  |  | 0 |  |  |  |  | 0 | 0 | 0 | 1 | 0 | 1 |
|  | OtsYRRD_seg_sp17 |  |  |  |  | 0 |  |  |  |  | 0 |  |  |  |  | 0 | 1 |  |  |  | 1 |  |  |  |  | 0 | 1 | 0 | 0 | 0 | 1 |
|  | OtsYRRD_seg_sp18 |  |  |  |  | 0 |  |  |  |  | 0 |  |  |  |  | 0 |  |  |  |  | 0 |  |  | 2 |  | 2 | 0 | 0 | 2 | 0 | 2 |
| 10_UCOLSP | OtsCHJO_seg_sp14 |  |  |  |  | 0 |  |  | 1 |  | 1 |  |  |  |  | 0 |  |  |  |  | 0 |  |  |  |  | 0 | 0 | 0 | 1 | 0 | 1 |
|  | OtsCHJO_seg_sp15 |  |  |  |  | 0 |  |  |  |  | 0 | 1 |  | 2 |  | 3 |  |  |  |  | 0 |  |  |  |  | 0 | 1 | 0 | 2 | 0 | 3 |
|  | OtsCHJO_seg_sp16 |  |  |  |  | 0 |  |  |  |  | 0 |  |  |  |  | 0 |  |  | 1 |  | 1 |  |  |  |  | 0 | 0 | 0 | 1 | 0 | 1 |
|  | OtsEAST_seg_sp16 |  |  |  |  | 0 |  |  |  |  | 0 |  |  |  |  | 0 | 1 |  |  |  | 1 |  |  |  |  | 0 | 1 | 0 | 0 | 0 | 1 |
|  | OtsEAST_seg_sp17 |  |  |  |  | 0 |  |  |  |  | 0 |  |  |  |  | 0 |  | 1 | 1 |  | 2 |  |  |  |  | 0 | 0 | 1 | 1 | 0 | 2 |
|  | OtsEAST_seg_sp18 |  |  |  |  | 0 |  |  |  |  | 0 |  |  |  |  | 0 |  |  |  |  | 0 |  |  | 1 | 1 | 2 | 0 | 0 | 1 | 1 | 2 |
|  | OtsLNFH_seg_sp13 |  |  | 1 |  | 1 |  |  |  |  | 0 |  |  |  |  | 0 |  |  |  |  | 0 |  |  |  |  | 0 | 0 | 0 | 1 | 0 | 1 |
|  | OtsLNFH_seg_sp14 |  |  |  |  | 0 |  |  | 2 |  | 2 |  |  |  |  | 0 |  |  |  |  | 0 |  |  |  |  | 0 | 0 | 0 | 2 | 0 | 2 |
|  | OtsLNFH_seg_sp15 |  |  |  |  | 0 |  |  |  |  | 0 |  |  | 2 |  | 2 |  |  |  |  | 0 |  |  |  |  | 0 | 0 | 0 | 2 | 0 | 2 |
|  | OtsLNFH_seg_sp17 |  |  |  |  | 0 |  |  |  |  | 0 |  |  |  |  | 0 | 5 |  | 7 |  | 12 |  |  |  |  | 0 | 5 | 0 | 7 | 0 | 12 |
|  | OtsLNFH_seg_sp18 |  |  |  |  | 0 |  |  |  |  | 0 |  |  |  |  | 0 |  |  |  |  | 0 | 1 |  | 9 |  | 10 | 1 | 0 | 9 | 0 | 10 |
|  | OtsMETH_seg_sp18 |  |  |  |  | 0 |  |  |  |  | 0 |  |  |  |  | 0 |  |  |  |  | 0 |  |  |  | 2 | 2 | 0 | 0 | 0 | 2 | 2 |
|  | OtsMETH_seg_sp19 |  |  |  |  | 0 |  |  |  |  | 0 |  |  |  |  | 0 |  |  |  |  | 0 |  |  |  | 1 | 1 | 0 | 0 | 0 | 1 | 1 |
|  | OtsWINT_seg_sp13 |  |  | 2 |  | 2 |  |  |  |  | 0 |  |  |  |  | 0 |  |  |  |  | 0 |  |  |  |  | 0 | 0 | 0 | 2 | 0 | 2 |
|  | OtsWINT_seg_sp14 |  |  |  |  | 0 | 1 |  | 4 | 2 | 7 |  |  |  |  | 0 |  |  |  |  | 0 |  |  |  |  | 0 | 1 | 0 | 4 | 2 | 7 |
|  | OtsWINT_seg_sp15 |  |  |  |  | 0 |  |  |  |  | 0 | 3 | 1 |  |  | 4 |  |  |  |  | 0 |  |  |  |  | 0 | 3 | 1 | 0 | 0 | 4 |
|  | OtsWINT_seg_sp17 |  |  |  |  | 0 |  |  |  |  | 0 |  |  |  |  | 0 |  | 2 | 1 | 1 | 4 |  |  |  |  | 0 | 0 | 2 | 1 | 1 | 4 |
|  | OtsWINT_seg_sp18 |  |  |  |  | 0 |  |  |  |  | 0 |  |  |  |  | 0 |  |  |  |  | 0 |  |  | 3 |  | 3 | 0 | 0 | 3 | 0 | 3 |
|  | OtsWINT_seg_sp19 |  |  |  |  | 0 |  |  |  |  | 0 |  |  |  |  | 0 |  |  |  |  | 0 |  |  | 1 |  | 1 | 0 | 0 | 1 | 0 | 1 |
| 11_TUCANO | OtsLYON_seg_sp13 |  |  |  | 1 | 1 |  |  |  |  | 0 |  |  |  |  | 0 |  |  |  |  | 0 |  |  |  |  | 0 | 0 | 0 | 0 | 1 | 1 |
|  | OtsLYON_seg_sp14 |  |  |  |  | 0 |  |  |  | 2 | 2 |  |  |  |  | 0 |  |  |  |  | 0 |  |  |  |  | 0 | 0 | 0 | 0 | 2 | 2 |
| 12_HELLSC | OtsCLWH_seg_sp13 |  |  | 8 | 1 | 9 |  |  |  |  | 0 |  |  |  |  | 0 |  |  |  |  | 0 |  |  |  |  | 0 | 0 | 0 | 8 | 1 | 9 |
|  | OtsCLWH_seg_sp14 |  |  |  |  | 0 | 1 |  | 17 |  | 18 |  |  |  |  | 0 |  |  |  |  | 0 |  |  |  |  | 0 | 1 | 0 | 17 | 0 | 18 |
|  | OtsCLWH_seg_sp15 |  |  |  |  | 0 |  |  |  |  | 0 |  |  | 4 |  | 4 |  |  |  |  | 0 |  |  |  |  | 0 | 0 | 0 | 4 | 0 | 4 |
|  | OtsCLWH_seg_sp18 |  |  |  |  | 0 |  |  |  |  | 0 |  |  |  |  | 0 |  |  |  |  | 0 | 1 |  | 14 | 1 | 16 | 1 | 0 | 14 | 1 | 16 |
|  | OtsCLWH_seg_sp19 |  |  |  |  | 0 |  |  |  |  | 0 |  |  |  |  | 0 |  |  |  |  | 0 |  |  | 1 |  | 1 | 0 | 0 | 1 | 0 | 1 |
|  | OtsDWOR_seg_sp12 |  |  | 6 |  | 6 |  |  |  |  | 0 |  |  |  |  | 0 |  |  |  |  | 0 |  |  |  |  | 0 | 0 | 0 | 6 | 0 | 6 |
|  | OtsDWOR_seg_sp13 |  |  | 4 | 1 | 5 |  |  |  |  | 0 |  |  |  |  | 0 |  |  |  |  | 0 |  |  |  |  | 0 | 0 | 0 | 4 | 1 | 5 |
|  | OtsDWOR_seg_sp14 |  |  |  |  | 0 | 3 |  | 7 |  | 10 |  |  |  |  | 0 |  |  |  |  | 0 |  |  |  |  | 0 | 3 | 0 | 7 | 0 | 10 |
|  | OtsDWOR_seg_sp15 |  |  |  |  | 0 |  |  |  |  | 0 |  |  | 5 | 1 | 6 |  |  |  |  | 0 |  |  |  |  | 0 | 0 | 0 | 5 | 1 | 6 |
|  | OtsDWOR_seg_sp17 |  |  |  |  | 0 |  |  |  |  | 0 |  |  |  |  | 0 | 1 |  | 6 | 1 | 8 |  |  |  |  | 0 | 1 | 0 | 6 | 1 | 8 |
|  | OtsDWOR_seg_sp18 |  |  |  |  | 0 |  |  |  |  | 0 |  |  |  |  | 0 |  |  |  |  | 0 |  |  | 20 | 1 | 21 | 0 | 0 | 20 | 1 | 21 |
|  | OtsIMNW_seg_ss15 |  |  |  |  | 0 |  |  |  |  | 0 |  |  | 1 |  | 1 |  |  |  |  | 0 |  |  |  |  | 0 | 0 | 0 | 1 | 0 | 1 |
|  | OtsIMNW_seg_ss17 |  |  |  |  | 0 |  |  |  |  | 0 |  |  |  |  | 0 |  |  | 1 |  | 1 |  |  |  |  | 0 | 0 | 0 | 1 | 0 | 1 |
|  | OtsKOOS_seg_sp17 |  |  |  |  | 0 |  |  |  |  | 0 |  |  |  |  | 0 |  |  | 4 | 2 | 6 |  |  |  |  | 0 | 0 | 0 | 4 | 2 | 6 |
|  | OtsKOOS_seg_sp18 |  |  |  |  | 0 |  |  |  |  | 0 |  |  |  |  | 0 |  |  |  |  | 0 | 1 |  | 9 | 3 | 13 | 1 | 0 | 9 | 3 | 13 |
|  | OtsLOOK_seg_sp13 |  | 2 |  |  | 2 |  |  |  |  | 0 |  |  |  |  | 0 |  |  |  |  | 0 |  |  |  |  | 0 | 0 | 2 | 0 | 0 | 2 |
|  | OtsLOOK_seg_sp14 |  |  |  |  | 0 | 1 |  | 6 | 1 | 8 |  |  |  |  | 0 |  |  |  |  | 0 |  |  |  |  | 0 | 1 | 0 | 6 | 1 | 8 |
|  | OtsLOOK_seg_sp15 |  |  |  |  | 0 |  |  |  |  | 0 |  |  | 1 |  | 1 |  |  |  |  | 0 |  |  |  |  | 0 | 0 | 0 | 1 | 0 | 1 |
|  | OtsLOOK_seg_sp17 |  |  |  |  | 0 |  |  |  |  | 0 |  |  |  |  | 0 |  |  | 2 | 2 | 4 |  |  |  |  | 0 | 0 | 0 | 2 | 2 | 4 |
|  | OtsLOOK_seg_sp18 |  |  |  |  | 0 |  |  |  |  | 0 |  |  |  |  | 0 |  |  |  |  | 0 | 1 |  | 2 |  | 3 | 1 | 0 | 2 | 0 | 3 |
|  | OtsNPFH_seg_sp14 |  |  |  |  | 0 |  |  |  | 1 | 1 |  |  |  |  | 0 |  |  |  |  | 0 |  |  |  |  | 0 | 0 | 0 | 0 | 1 | 1 |
|  | OtsPOWP_seg_sp12 | 1 |  |  |  | 1 |  |  |  |  | 0 |  |  |  |  | 0 |  |  |  |  | 0 |  |  |  |  | 0 | 1 | 0 | 0 | 0 | 1 |
|  | OtsPOWP_seg_sp13 |  |  | 4 |  | 4 |  |  |  |  | 0 |  |  |  |  | 0 |  |  |  |  | 0 |  |  |  |  | 0 | 0 | 0 | 4 | 0 | 4 |
|  | OtsPOWP_seg_sp14 |  |  |  |  | 0 | 1 | 1 | 10 | 2 | 14 |  |  |  |  | 0 |  |  |  |  | 0 |  |  |  |  | 0 | 1 | 1 | 10 | 2 | 14 |
|  | OtsPOWP_seg_sp15 |  |  |  |  | 0 |  |  |  |  | 0 |  |  | 5 | 2 | 7 |  |  |  |  | 0 |  |  |  |  | 0 | 0 | 0 | 5 | 2 | 7 |
|  | OtsPOWP_seg_sp17 |  |  |  |  | 0 |  |  |  |  | 0 |  |  |  |  | 0 |  |  |  |  | 0 |  |  |  | 1 | 1 | 0 | 0 | 0 | 1 | 1 |
|  | OtsPOWP_seg_sp18 |  |  |  |  | 0 |  |  |  |  | 0 |  |  |  |  | 0 |  |  |  |  | 0 |  |  | 1 | 1 | 2 | 0 | 0 | 1 | 1 | 2 |
|  | OtsRAPH_seg_sp12 |  |  | 2 |  | 2 |  |  |  |  | 0 |  |  |  |  | 0 |  |  |  |  | 0 |  |  |  |  | 0 | 0 | 0 | 2 | 0 | 2 |
|  | OtsRAPH_seg_sp13 | 1 |  | 29 |  | 30 |  |  | 1 |  | 1 |  |  |  |  | 0 |  |  |  |  | 0 |  |  |  |  | 0 | 1 | 0 | 30 | 0 | 31 |
|  | OtsRAPH_seg_sp14 |  |  |  |  | 0 | 5 |  | 24 |  | 29 |  |  | 1 |  | 1 |  |  |  |  | 0 |  |  |  |  | 0 | 5 | 0 | 25 | 0 | 30 |
|  | OtsRAPH_seg_sp15 |  |  |  |  | 0 |  |  |  |  | 0 | 4 |  | 10 |  | 14 |  |  |  |  | 0 |  |  |  |  | 0 | 4 | 0 | 10 | 0 | 14 |
|  | OtsRAPH_seg_sp16 |  |  |  |  | 0 |  |  |  |  | 0 |  |  |  |  | 0 |  |  | 1 |  | 1 |  |  |  |  | 0 | 0 | 0 | 1 | 0 | 1 |
|  | OtsRAPH_seg_sp17 |  |  |  |  | 0 |  |  |  |  | 0 |  |  |  |  | 0 | 2 |  | 6 |  | 8 |  |  |  |  | 0 | 2 | 0 | 6 | 0 | 8 |
|  | OtsRAPH_seg_sp18 |  |  |  |  | 0 |  |  |  |  | 0 |  |  |  |  | 0 |  |  |  |  | 0 | 3 |  | 35 | 1 | 39 | 3 | 0 | 35 | 1 | 39 |
| 13_SFSALM | OtsMCCA_seg_ss14 |  |  |  |  | 0 |  |  | 1 |  | 1 |  |  |  |  | 0 |  |  |  |  | 0 |  |  |  |  | 0 | 0 | 0 | 1 | 0 | 1 |
|  | OtsMCCA_seg_ss15 |  |  |  |  | 0 |  |  |  |  | 0 |  |  | 1 |  | 1 |  |  |  |  | 0 |  |  |  |  | 0 | 0 | 0 | 1 | 0 | 1 |
|  | OtsMCCA_seg_ss17 |  |  |  |  | 0 |  |  |  |  | 0 |  |  |  |  | 0 |  |  |  |  | 0 |  |  | 1 |  | 1 | 0 | 0 | 1 | 0 | 1 |
|  | OtsMCCA_seg_ss18 |  |  |  |  | 0 |  |  |  |  | 0 |  |  |  |  | 0 |  |  |  |  | 0 |  |  | 9 | 2 | 11 | 0 | 0 | 9 | 2 | 11 |
| 16_UPSALM | OtsPAHH_seg_ss18 |  |  |  |  | 0 |  |  |  |  | 0 |  |  |  |  | 0 |  |  |  |  | 0 |  |  | 1 |  | 1 | 0 | 0 | 1 | 0 | 1 |
|  | OtsSAWT_seg_ss13 |  |  | 1 |  | 1 |  |  |  |  | 0 |  |  |  |  | 0 |  |  |  |  | 0 |  |  |  |  | 0 | 0 | 0 | 1 | 0 | 1 |
|  | OtsSAWT_seg_ss14 |  |  |  |  | 0 |  |  | 7 |  | 7 |  |  | 1 |  | 1 |  |  |  |  | 0 |  |  |  |  | 0 | 0 | 0 | 8 | 0 | 8 |
|  | OtsSAWT_seg_ss15 |  |  |  |  | 0 |  |  |  |  | 0 |  |  | 1 |  | 1 |  |  |  |  | 0 |  |  |  |  | 0 | 0 | 0 | 1 | 0 | 1 |
|  | OtsSAWT_seg_ss17 |  |  |  |  | 0 |  |  |  |  | 0 |  |  |  |  | 0 | 1 |  | 1 |  | 2 |  |  |  |  | 0 | 1 | 0 | 1 | 0 | 2 |
|  | OtsSAWT_seg_ss18 |  |  |  |  | 0 |  |  |  |  | 0 |  |  |  |  | 0 |  |  |  |  | 0 |  |  | 8 | 1 | 9 | 0 | 0 | 8 | 1 | 9 |
| 18_UCOLSF | OtsCHJO_seg_su17 |  |  |  |  | 0 |  |  |  |  | 0 |  |  |  |  | 0 |  |  |  |  | 0 | 1 |  |  |  | 1 | 1 | 0 | 0 | 0 | 1 |
|  | OtsEAST_seg_su17 |  |  |  |  | 0 |  |  |  |  | 0 |  |  |  |  | 0 |  |  |  |  | 0 | 3 |  |  |  | 3 | 3 | 0 | 0 | 0 | 3 |
|  | OtsEAST_seg_su18 |  |  |  |  | 0 |  |  |  |  | 0 |  |  |  |  | 0 |  |  |  |  | 0 | 1 |  |  |  | 1 | 1 | 0 | 0 | 0 | 1 |
| 20_BONPOOLSP | OtsCARS_seg_sp12 |  |  | 1 |  | 1 |  |  |  |  | 0 |  |  |  |  | 0 |  |  |  |  | 0 |  |  |  |  | 0 | 0 | 0 | 1 | 0 | 1 |
|  | OtsCARS_seg_sp13 |  | 1 | 4 |  | 5 |  |  | 1 |  | 1 |  |  |  |  | 0 |  |  |  |  | 0 |  |  |  |  | 0 | 0 | 1 | 5 | 0 | 6 |
|  | OtsCARS_seg_sp14 |  |  |  |  | 0 | 4 |  | 15 | 1 | 20 |  |  | 2 |  | 2 |  |  |  |  | 0 |  |  |  |  | 0 | 4 | 0 | 17 | 1 | 22 |
|  | OtsCARS_seg_sp15 |  |  |  |  | 0 |  |  |  |  | 0 | 3 |  | 4 |  | 7 |  |  |  |  | 0 |  |  |  |  | 0 | 3 | 0 | 4 | 0 | 7 |
|  | OtsCARS_seg_sp17 |  |  |  |  | 0 |  |  |  |  | 0 |  |  |  |  | 0 |  |  | 8 |  | 8 |  |  |  |  | 0 | 0 | 0 | 8 | 0 | 8 |
|  | OtsCARS_seg_sp18 |  |  |  |  | 0 |  |  |  |  | 0 |  |  |  |  | 0 |  |  |  |  | 0 | 1 |  | 13 |  | 14 | 1 | 0 | 13 | 0 | 14 |
|  | OtsLWSN_seg_sp13 |  |  | 9 |  | 9 | 1 |  | 2 |  | 3 |  |  |  |  | 0 |  |  |  |  | 0 |  |  |  |  | 0 | 1 | 0 | 11 | 0 | 12 |
|  | OtsLWSN_seg_sp14 |  |  |  |  | 0 | 8 |  | 35 | 1 | 44 |  |  | 2 |  | 2 |  |  |  |  | 0 |  |  |  |  | 0 | 8 | 0 | 37 | 1 | 46 |
|  | OtsLWSN_seg_sp15 |  |  |  |  | 0 |  |  |  |  | 0 | 2 |  | 8 |  | 10 |  |  |  |  | 0 |  |  |  |  | 0 | 2 | 0 | 8 | 0 | 10 |
|  | OtsLWSN_seg_sp16 |  |  |  |  | 0 |  |  |  |  | 0 |  |  |  |  | 0 |  |  | 1 |  | 1 |  |  |  |  | 0 | 0 | 0 | 1 | 0 | 1 |
|  | OtsLWSN_seg_sp17 |  |  |  |  | 0 |  |  |  |  | 0 |  |  |  |  | 0 |  |  | 2 |  | 2 |  |  | 1 |  | 1 | 0 | 0 | 3 | 0 | 3 |
|  | OtsLWSN_seg_sp18 |  |  |  |  | 0 |  |  |  |  | 0 |  |  |  |  | 0 |  |  |  |  | 0 | 3 |  | 14 | 1 | 18 | 3 | 0 | 14 | 1 | 18 |
| 21_UMATILLASP | OtsUMAT_seg_sp13 |  |  | 15 |  | 15 |  |  |  |  | 0 |  |  |  |  | 0 |  |  |  |  | 0 |  |  |  |  | 0 | 0 | 0 | 15 | 0 | 15 |
|  | OtsUMAT_seg_sp14 |  |  |  |  | 0 | 1 |  | 14 | 2 | 17 |  |  | 1 |  | 1 |  |  |  |  | 0 |  |  |  |  | 0 | 1 | 0 | 15 | 2 | 18 |
|  | OtsUMAT_seg_sp17 |  |  |  |  | 0 |  |  |  |  | 0 |  |  |  |  | 0 |  |  | 1 | 1 | 2 |  |  |  |  | 0 | 0 | 0 | 1 | 1 | 2 |
|  | OtsUMAT_seg_sp18 |  |  |  |  | 0 |  |  |  |  | 0 |  |  |  |  | 0 |  |  |  |  | 0 |  |  | 1 | 1 | 2 | 0 | 0 | 1 | 1 | 2 |

Note: Assignments were classified by year, Lower and Upriver stocks based on VSI, and hatchery origin clipped “H” and hatchery no-clip “HNC”. PBT assignments were used to identify hatchery origin fish that did not have an adipose clip (“HNC”). All hatchery broodstock were classified into GenStock Expected categories, of which, 02_WCASSP and 04_WILLAM represent Lower stocks and all others are upriver. The broodstock names are provided as codes (fully described in Table S2) and include a two-digit Spawn Year which can be used to effectively age each fish. For example, Spawn Year “18” is a 4 year old fish if it were collected in the 2021 Test Fishery.

Table S5. Estimated CPUE in units of GenStock for the Spring Chinook Salmon Test Fishery (2017 – 2022).

|  |  | 2017 | | | | | | | 2018 | | | | | | | 2019 | | | | | | |
| --- | --- | --- | --- | --- | --- | --- | --- | --- | --- | --- | --- | --- | --- | --- | --- | --- | --- | --- | --- | --- | --- | --- |
|  |  | Lower (VSI) | | | Upriver (VSI) | | |  | Lower (VSI) | | | Upriver (VSI) | | |  | Lower (VSI) | | | Upriver (VSI) | | |  |
|  | GenStock | H | HNC | W | H | HNC | W | Total | H | HNC | W | H | HNC | W | Total | H | HNC | W | H | HNC | W | Total |
| Lower | 01_YOUNGS |  |  |  |  |  |  | 0.0000 |  |  |  |  |  |  | 0.0000 | 0.0000 |  |  | 0.0307 |  |  | 0.0307 |
|  | 02_WCASSP | 2.3593 |  |  | 0.9766 |  |  | 3.3359 | 0.4233 |  | 0.1458 | 0.5769 |  | 0.0000 | 1.1460 | 0.6495 |  |  | 0.1721 |  |  | 0.8215 |
|  | 03_WCASFA |  |  |  |  |  |  | 0.0000 |  |  |  |  |  |  | 0.0000 |  |  |  |  |  |  | 0.0000 |
|  | 04_WILLAM | 1.5358 |  | 0.3538 | 0.8087 |  | 0.4351 | 3.1334 | 4.3958 |  | 0.5178 | 1.1066 |  | 0.0000 | 6.0203 | 3.4478 |  | 1.6198 | 0.5052 |  | 0.2357 | 5.8085 |
|  | Subtotal Lower GenStock | 3.8951 | 0.0000 | 0.3538 | 1.7853 | 0.0000 | 0.4351 | 6.4693 | 4.8191 | 0.0000 | 0.6636 | 1.6835 | 0.0000 | 0.0000 | 7.1662 | 4.0973 | 0.0000 | 1.6198 | 0.7080 | 0.0000 | 0.2357 | 6.6607 |
|  | 05_SPCRTU |  |  |  |  |  |  | 0.0000 |  |  |  |  |  |  | 0.0000 |  |  |  |  |  |  | 0.0000 |
| Upriver | 06_KLICKR | 0.0749 |  |  | 0.0000 |  |  | 0.0749 | 0.1504 |  |  | 0.0728 |  |  | 0.2232 | 0.0733 |  |  | 0.0000 |  |  | 0.0733 |
|  | 07_DESCSP | 0.0000 |  | 0.0000 | 0.1858 |  | 0.1471 | 0.3328 | 0.2003 |  | 0.0000 | 0.2258 |  | 0.3508 | 0.7769 | 0.0000 |  |  | 0.6080 |  |  | 0.6080 |
|  | 08_JOHNDR |  |  |  |  |  |  | 0.0000 |  |  | 0.0000 |  |  | 0.0570 | 0.0570 |  |  |  |  |  |  | 0.0000 |
|  | 09_YAKIMA | 0.0000 |  | 0.1669 | 0.1036 |  | 0.5700 | 0.8405 | 0.0000 |  | 0.1458 | 0.3737 |  | 0.4593 | 0.9788 | 0.1474 |  | 0.0000 | 0.6936 |  | 0.4714 | 1.3124 |
|  | 10_UCOLSP | 0.0749 |  | 0.1569 | 1.1603 |  | 0.1410 | 1.5330 | 0.2761 | 0.0000 | 0.0000 | 0.7043 | 0.2995 | 0.5153 | 1.7951 | 0.3607 | 0.0733 | 0.0733 | 0.4707 | 0.0000 | 0.5533 | 1.5314 |
|  | 11_TUCANO |  | 0.0000 |  |  | 0.1410 |  | 0.1410 |  | 0.0000 |  |  | 0.2225 |  | 0.2225 |  |  | 0.1467 |  |  | 0.0000 | 0.1467 |
|  | 12_HELLSC | 0.2246 | 0.1918 | 0.0000 | 5.2024 | 0.2910 | 0.1320 | 6.0418 | 0.8197 | 0.0619 | 0.0000 | 5.1113 | 0.5892 | 0.3162 | 6.8983 | 0.3109 | 0.0000 | 0.1488 | 2.2212 | 0.3327 | 0.3814 | 3.3951 |
|  | 13_SFSALM | 0.0000 |  |  | 0.0727 |  |  | 0.0727 | 0.0000 |  | 0.0625 | 0.1502 |  | 0.0000 | 0.2127 | 0.0000 |  |  | 0.0845 |  |  | 0.0845 |
|  | 14_CHMBLN |  |  |  |  |  |  | 0.0000 |  |  |  |  |  |  | 0.0000 |  |  |  |  |  |  | 0.0000 |
|  | 15_MFSALM |  |  |  |  |  |  | 0.0000 |  |  |  |  |  |  | 0.0000 |  |  | 0.0000 |  |  | 0.1468 | 0.1468 |
|  | 16_UPSALM | 0.0000 |  |  | 0.1101 |  |  | 0.1101 | 0.0000 |  |  | 0.5382 |  |  | 0.5382 | 0.0000 |  |  | 0.1654 |  |  | 0.1654 |
|  | 17_DESCFA |  |  |  |  |  |  | 0.0000 |  |  |  |  |  |  | 0.0000 |  |  |  |  |  |  | 0.0000 |
|  | 18_UCOLSF |  |  |  |  |  |  | 0.0000 |  |  |  |  |  |  | 0.0000 |  |  |  |  |  |  | 0.0000 |
|  | 19_SRFALL |  |  |  |  |  |  | 0.0000 |  |  |  |  |  |  | 0.0000 |  |  |  |  |  |  | 0.0000 |
|  | 20_BONPOOLSP | 0.0000 | 0.1138 |  | 1.5653 | 0.0000 |  | 1.6791 | 0.9394 | 0.0000 |  | 4.2307 | 0.2827 |  | 5.4528 | 0.3711 |  |  | 1.2710 |  |  | 1.6421 |
|  | 21_UMATILLASP | 0.0000 |  |  | 1.6082 |  |  | 1.6082 | 0.0632 | 0.0000 |  | 1.0635 | 0.1719 |  | 1.2986 | 0.0000 |  |  | 0.0717 |  |  | 0.0717 |
|  | Subtotal Upriver GenStock | 0.3743 | 0.3056 | 0.3237 | 10.0083 | 0.4320 | 0.9900 | 12.4341 | 2.4490 | 0.0619 | 0.2083 | 12.4704 | 1.5658 | 1.6987 | 18.4541 | 1.2635 | 0.0733 | 0.3688 | 5.5861 | 0.3327 | 1.5530 | 9.1774 |

Table S5(continued). Estimated CPUE in units of GenStock for the Spring Chinook Salmon Test Fishery (2017 – 2022).

|  |  | 2021 | | | | | | | 2022 | | | | | | |
| --- | --- | --- | --- | --- | --- | --- | --- | --- | --- | --- | --- | --- | --- | --- | --- |
|  |  | Lower (VSI) | | | Upriver (VSI) | | |  | Lower (VSI) | | | Upriver (VSI) | | |  |
|  | GenStock | H | HNC | W | H | HNC | W | Total | H | HNC | W | H | HNC | W | Total |
| Lower | 01_YOUNGS |  |  |  |  |  |  | 0.0000 |  |  |  |  |  |  | 0.0000 |
|  | 02_WCASSP | 0.3370 |  |  | 0.4775 |  |  | 0.8144 | 2.1397 |  |  | 1.3720 |  |  | 3.5117 |
|  | 03_WCASFA |  |  |  |  |  |  | 0.0000 |  |  |  |  |  |  | 0.0000 |
|  | 04_WILLAM | 2.9800 |  | 0.1034 | 0.0000 |  | 0.1311 | 3.2146 | 8.0320 |  | 0.7339 | 0.7185 |  | 0.1971 | 9.6816 |
|  | Subtotal Lower GenStock | 3.3170 | 0.0000 | 0.1034 | 0.4775 | 0.0000 | 0.1311 | 4.0290 | 10.1717 | 0.0000 | 0.7339 | 2.0905 | 0.0000 | 0.1971 | 13.1932 |
|  | 05_SPCRTU |  |  |  |  |  |  | 0.0000 |  |  |  |  |  |  | 0.0000 |
| Upriver | 06_KLICKR | 0.1220 |  |  | 0.2283 |  |  | 0.3502 | 0.0000 |  |  | 0.4651 |  |  | 0.4651 |
|  | 07_DESCSP | 0.1567 | 0.0000 |  | 0.4202 | 0.1475 |  | 0.7244 | 0.0000 |  |  | 0.7613 |  |  | 0.7613 |
|  | 08_JOHNDR | 0.0000 |  | 0.1034 | 0.1166 |  | 0.2802 | 0.5002 |  |  |  |  |  |  | 0.0000 |
|  | 09_YAKIMA | 0.1153 |  | 0.1034 | 0.0000 |  | 0.2623 | 0.4810 | 0.1605 |  | 0.0909 | 0.6830 |  | 0.2990 | 1.2334 |
|  | 10_UCOLSP | 0.8910 | 0.4013 | 0.1014 | 1.7418 | 0.1582 | 0.2386 | 3.5324 | 0.2537 | 0.0000 | 0.0000 | 3.4216 | 0.4199 | 1.2001 | 5.2953 |
|  | 11_TUCANO |  |  |  |  |  |  | 0.0000 |  |  |  |  |  |  | 0.0000 |
|  | 12_HELLSC | 0.4667 | 0.0000 | 0.1034 | 2.9918 | 0.7080 | 0.1147 | 4.3847 | 0.8713 | 0.0000 | 0.0877 | 11.1729 | 0.8569 | 1.2528 | 14.2416 |
|  | 13_SFSALM |  |  | 0.1034 |  |  | 0.1491 | 0.2525 | 0.0000 | 0.0000 | 0.0000 | 1.2408 | 0.1873 | 0.2955 | 1.7236 |
|  | 14_CHMBLN |  |  |  |  |  |  | 0.0000 |  |  |  |  |  |  | 0.0000 |
|  | 15_MFSALM |  |  | 0.0000 |  |  | 0.1491 | 0.1491 |  |  | 0.0000 |  |  | 0.2278 | 0.2278 |
|  | 16_UPSALM | 0.1250 |  |  | 0.1303 |  |  | 0.2553 | 0.0000 | 0.0000 | 0.0000 | 1.1406 | 0.0832 | 0.2037 | 1.4275 |
|  | 17_DESCFA |  |  |  |  |  |  | 0.0000 |  |  |  |  |  |  | 0.0000 |
|  | 18_UCOLSF |  |  |  |  |  |  | 0.0000 | 0.9381 |  |  | 0.0000 |  |  | 0.9381 |
|  | 19_SRFALL |  |  |  |  |  |  | 0.0000 |  |  |  |  |  |  | 0.0000 |
|  | 20_BONPOOLSP | 0.0000 |  |  | 1.2996 |  |  | 1.2996 | 0.5859 | 0.0000 |  | 3.9764 | 0.1213 |  | 4.6836 |
|  | 21_UMATILLASP | 0.0000 | 0.0000 |  | 0.1114 | 0.1494 |  | 0.2607 | 0.0000 | 0.0000 |  | 0.1543 | 0.1149 |  | 0.2692 |
|  | Subtotal Upriver GenStock | 1.8767 | 0.4013 | 0.5152 | 7.0400 | 1.1630 | 1.1939 | 12.1902 | 2.8094 | 0.0000 | 0.1787 | 23.0160 | 1.7835 | 3.4788 | 31.2665 |

Note: The estimated CPUE is listed under the units of lower and upriver stocks classified by VSI and then further parsed into hatchery clipped (H), hatchery unclipped (HNC), and natural origin/wild (W) based on the information from genetic analysis.

Table S6. The Bonneville Dam GenStock assignments

|  |  | 2017 | | | | 2018 | | | | 2019 | | | | 2021 | | | | 2022 | | | |
| --- | --- | --- | --- | --- | --- | --- | --- | --- | --- | --- | --- | --- | --- | --- | --- | --- | --- | --- | --- | --- | --- |
|  | GenStock | H | HNC | W | Total | H | HNC | W | Total | H | HNC | W | Total | H | HNC | W | Total | H | HNC | W | Total |
| Lower | 01_YOUNGS |  |  |  |  | 1 |  |  | 1 |  |  |  |  |  |  |  |  | 1 |  |  | 1 |
|  | 02_WCASSP | 8 |  | 1 | 9 | 26 | 1 |  | 27 | 16 | 1 |  | 17 | 8 |  |  | 8 | 15 | 1 |  | 16 |
|  | 03_WCASFA |  |  | 1 | 1 |  |  |  |  |  |  |  |  |  |  |  |  |  |  |  |  |
|  | 04_WILLAM | 3 |  | 1 | 4 | 3 |  |  | 3 | 10 |  |  | 10 | 1 |  |  | 1 | 3 |  | 3 | 6 |
|  | Subtotal Lower GenStock | 11 | 0 | 3 | 14 | 30 | 1 | 0 | 31 | 26 | 1 | 0 | 27 | 9 | 0 | 0 | 9 | 19 | 1 | 3 | 23 |
|  | 05_SPCRTU |  |  |  |  |  |  |  |  |  |  |  |  |  |  |  |  |  |  |  |  |
| Upriver | 06_KLICKR | 28 |  | 2 | 30 | 11 |  | 1 | 12 | 2 |  |  | 2 | 22 |  | 2 | 24 | 13 | 1 | 2 | 16 |
|  | 07_DESCSP | 59 | 2 | 1 | 62 | 26 | 2 | 11 | 39 | 20 | 2 | 9 | 31 | 64 | 3 | 5 | 72 | 37 |  | 6 | 43 |
|  | 08_JOHNDR | 1 |  | 4 | 5 |  |  | 10 | 10 |  |  | 9 | 9 | 1 |  | 4 | 5 |  |  | 3 | 3 |
|  | 09_YAKIMA | 22 |  | 24 | 46 | 38 |  | 37 | 75 | 15 | 1 | 18 | 34 | 20 | 2 | 26 | 48 | 15 | 1 | 16 | 32 |
|  | 10_UCOLSP | 57 | 6 | 15 | 78 | 70 | 20 | 48 | 138 | 118 | 40 | 47 | 205 | 126 | 49 | 39 | 214 | 102 | 32 | 58 | 192 |
|  | 11_TUCANO |  | 4 |  | 4 |  | 7 | 4 | 11 |  | 2 | 2 | 4 |  |  |  |  |  |  | 1 | 1 |
|  | 12_HELLSC | 265 | 16 | 22 | 303 | 374 | 37 | 91 | 502 | 358 | 26 | 70 | 454 | 403 | 36 | 41 | 480 | 281 | 35 | 78 | 394 |
|  | 13_SFSALM | 32 | 14 | 6 | 52 | 36 | 11 | 22 | 69 | 43 | 14 | 20 | 77 | 58 | 20 | 21 | 99 | 29 | 10 | 44 | 83 |
|  | 14_CHMBLN |  |  |  |  |  |  | 4 | 4 |  |  |  |  |  |  | 2 | 2 | 1 |  | 6 | 7 |
|  | 15_MFSALM |  |  | 2 | 2 |  |  | 11 | 11 |  |  | 6 | 6 | 1 |  | 10 | 11 | 1 |  | 19 | 20 |
|  | 16_UPSALM | 31 | 1 | 12 | 44 | 90 | 6 | 11 | 107 | 35 |  | 18 | 53 | 20 | 8 | 26 | 54 | 48 | 7 | 29 | 84 |
|  | 17_DESCFA |  |  |  |  |  |  |  |  |  |  |  |  |  |  |  |  |  |  |  |  |
|  | 18_UCOLSF | 98 | 5 | 115 | 218 | 126 | 11 | 45 | 182 | 203 | 9 | 45 | 257 | 202 | 19 | 84 | 305 | 117 | 14 | 49 | 180 |
|  | 19_SRFALL | 2 |  | 2 | 4 | 1 |  | 1 | 2 |  |  | 2 | 2 |  |  | 1 | 1 |  |  |  |  |
|  | 20_BONPOOLSP | 108 | 7 |  | 115 | 141 | 9 |  | 150 | 128 | 4 |  | 132 | 114 | 4 |  | 118 | 111 | 9 |  | 120 |
|  | 21_UMATILLASP | 64 | 3 |  | 67 | 78 | 12 |  | 90 | 5 | 12 |  | 17 | 27 | 2 |  | 29 | 21 | 5 |  | 26 |
|  | Subtotal Upriver GenStock | 767 | 58 | 205 | 1030 | 991 | 115 | 296 | 1402 | 927 | 110 | 246 | 1283 | 1058 | 143 | 261 | 1462 | 776 | 114 | 311 | 1201 |

Note: The assignments are parsed into hatchery clipped (H), hatchery unclipped (HNC), and natural origin/wild (W) based on the information from genetic analysis

Table S7. Bonneville Dam Broodstock assignments (2017 – 2022).

| Gen Stock |  | 2017 | | | 2018 | | | 2019 | | | 2021 | | | 2022 | | | Grand Total | | |
| --- | --- | --- | --- | --- | --- | --- | --- | --- | --- | --- | --- | --- | --- | --- | --- | --- | --- | --- | --- |
| Exp | Hatchery broodstock | H | HNC | Total | H | HNC | Total | H | HNC | Total | H | HNC | Total | H | HNC | Total | H | HNC | Total |
| 02_WCASSP | OtsPARK_seg_sp13 | 2 |  | 2 |  |  | 0 |  |  | 0 |  |  | 0 |  |  | 0 | 2 | 0 | 2 |
|  | OtsPARK_seg_sp14 | 5 |  | 5 | 26 | 1 | 27 | 1 |  | 1 |  |  | 0 |  |  | 0 | 32 | 1 | 33 |
|  | OtsPARK_seg_sp15 |  |  | 0 |  |  | 0 | 14 | 1 | 15 |  |  | 0 |  |  | 0 | 14 | 1 | 15 |
|  | OtsPARK_seg_sp16 |  |  | 0 |  |  | 0 |  |  | 0 | 1 |  | 1 |  |  | 0 | 1 | 0 | 1 |
|  | OtsPARK_seg_sp17 |  |  | 0 |  |  | 0 |  |  | 0 | 6 |  | 6 |  |  | 0 | 6 | 0 | 6 |
|  | OtsPARK_seg_sp18 |  |  | 0 |  |  | 0 |  |  | 0 | 1 |  | 1 | 15 | 1 | 16 | 16 | 1 | 17 |
| 06_KLICKR | OtsKLIC_seg_sp12 | 3 |  | 3 |  |  | 0 |  |  | 0 |  |  | 0 |  |  | 0 | 3 | 0 | 3 |
|  | OtsKLIC_seg_sp13 | 23 |  | 23 | 2 |  | 2 |  |  | 0 |  |  | 0 |  |  | 0 | 25 | 0 | 25 |
|  | OtsKLIC_seg_sp14 | 2 |  | 2 | 9 |  | 9 | 1 |  | 1 |  |  | 0 |  |  | 0 | 12 | 0 | 12 |
|  | OtsKLIC_seg_sp16 |  |  | 0 |  |  | 0 |  |  | 0 | 4 |  | 4 |  |  | 0 | 4 | 0 | 4 |
|  | OtsKLIC_seg_sp17 |  |  | 0 |  |  | 0 |  |  | 0 | 14 |  | 14 |  |  | 0 | 14 | 0 | 14 |
|  | OtsKLIC_seg_sp18 |  |  | 0 |  |  | 0 |  |  | 0 | 1 |  | 1 | 13 | 1 | 14 | 14 | 1 | 15 |
| 07_DESCSP | OtsRBFH_seg_sp13 | 23 | 1 | 24 |  |  | 0 |  |  | 0 |  |  | 0 |  |  | 0 | 23 | 1 | 24 |
|  | OtsRBFH_seg_sp14 | 9 |  | 9 | 16 | 1 | 17 |  |  | 0 |  |  | 0 |  |  | 0 | 25 | 1 | 26 |
|  | OtsRBFH_seg_sp15 |  |  | 0 |  |  | 0 | 8 | 2 | 10 |  |  | 0 |  |  | 0 | 8 | 2 | 10 |
|  | OtsRBFH_seg_sp17 |  |  | 0 |  |  | 0 |  |  | 0 | 7 | 1 | 8 |  |  | 0 | 7 | 1 | 8 |
|  | OtsRBFH_seg_sp18 |  |  | 0 |  |  | 0 |  |  | 0 | 3 | 1 | 4 | 20 |  | 20 | 23 | 1 | 24 |
|  | OtsWSNF_seg_sp13 | 19 | 1 | 20 |  |  | 0 |  |  | 0 |  |  | 0 |  |  | 0 | 19 | 1 | 20 |
|  | OtsWSNF_seg_sp14 |  |  | 0 |  | 1 | 1 |  |  | 0 |  |  | 0 |  |  | 0 | 0 | 1 | 1 |
|  | OtsWSNF_seg_sp15 |  |  | 0 |  |  | 0 | 3 |  | 3 |  |  | 0 |  |  | 0 | 3 | 0 | 3 |
|  | OtsWSNF_seg_sp17 |  |  | 0 |  |  | 0 |  |  | 0 | 50 | 1 | 51 | 1 |  | 1 | 51 | 1 | 52 |
|  | OtsWSNF_seg_sp18 |  |  | 0 |  |  | 0 |  |  | 0 |  |  | 0 | 12 |  | 12 | 12 | 0 | 12 |
| 09_YAKIMA | OtsYRRD_int_sp13 | 4 |  | 4 |  |  | 0 |  |  | 0 |  |  | 0 |  |  | 0 | 4 | 0 | 4 |
|  | OtsYRRD_int_sp14 | 2 |  | 2 | 32 |  | 32 |  |  | 0 |  |  | 0 |  |  | 0 | 34 | 0 | 34 |
|  | OtsYRRD_int_sp15 |  |  | 0 |  |  | 0 | 13 | 1 | 14 |  |  | 0 |  |  | 0 | 13 | 1 | 14 |
|  | OtsYRRD_int_sp17 |  |  | 0 |  |  | 0 |  |  | 0 | 14 | 1 | 15 |  |  | 0 | 14 | 1 | 15 |
|  | OtsYRRD_int_sp18 |  |  | 0 |  |  | 0 |  |  | 0 |  |  | 0 | 10 | 1 | 11 | 10 | 1 | 11 |
|  | OtsYRRD_seg_sp13 | 3 |  | 3 |  |  | 0 |  |  | 0 |  |  | 0 |  |  | 0 | 3 | 0 | 3 |
|  | OtsYRRD_seg_sp14 |  |  | 0 | 4 |  | 4 |  |  | 0 |  |  | 0 |  |  | 0 | 4 | 0 | 4 |
|  | OtsYRRD_seg_sp15 |  |  | 0 |  |  | 0 | 2 |  | 2 |  |  | 0 |  |  | 0 | 2 | 0 | 2 |
|  | OtsYRRD_seg_sp17 |  |  | 0 |  |  | 0 |  |  | 0 | 3 | 1 | 4 |  |  | 0 | 3 | 1 | 4 |
|  | OtsYRRD_seg_sp18 |  |  | 0 |  |  | 0 |  |  | 0 |  |  | 0 | 3 |  | 3 | 3 | 0 | 3 |
| 10_UCOLSP | OtsCHJO_seg_sp14 |  |  | 0 | 6 | 2 | 8 |  |  | 0 |  |  | 0 |  |  | 0 | 6 | 2 | 8 |
|  | OtsCHJO_seg_sp15 |  |  | 0 |  |  | 0 | 31 | 1 | 32 |  |  | 0 |  |  | 0 | 31 | 1 | 32 |
|  | OtsCHJO_seg_sp17 |  |  | 0 |  |  | 0 |  |  | 0 | 7 |  | 7 |  |  | 0 | 7 | 0 | 7 |
|  | OtsEAST_seg_sp13 | 8 | 1 | 9 |  |  | 0 |  |  | 0 |  |  | 0 |  |  | 0 | 8 | 1 | 9 |
|  | OtsEAST_seg_sp14 |  | 2 | 2 | 7 | 3 | 10 |  | 1 | 1 |  |  | 0 |  |  | 0 | 7 | 6 | 13 |
|  | OtsEAST_seg_sp15 |  |  | 0 |  | 1 | 1 | 5 | 14 | 19 |  |  | 0 |  |  | 0 | 5 | 15 | 20 |
|  | OtsEAST_seg_sp16 |  |  | 0 |  |  | 0 |  | 1 | 1 |  |  | 0 |  |  | 0 | 0 | 1 | 1 |
|  | OtsEAST_seg_sp17 |  |  | 0 |  |  | 0 |  |  | 0 | 11 | 18 | 29 |  |  | 0 | 11 | 18 | 29 |
|  | OtsEAST_seg_sp18 |  |  | 0 |  |  | 0 |  |  | 0 |  |  | 0 | 9 | 21 | 30 | 9 | 21 | 30 |
|  | OtsEAST_seg_sp19 |  |  | 0 |  |  | 0 |  |  | 0 |  |  | 0 |  | 1 | 1 | 0 | 1 | 1 |
|  | OtsLNFH_seg_sp13 | 11 |  | 11 |  |  | 0 |  |  | 0 |  |  | 0 |  |  | 0 | 11 | 0 | 11 |
|  | OtsLNFH_seg_sp14 |  |  | 0 | 14 | 1 | 15 |  |  | 0 |  |  | 0 |  |  | 0 | 14 | 1 | 15 |
|  | OtsLNFH_seg_sp15 |  |  | 0 |  |  | 0 | 29 | 1 | 30 |  |  | 0 |  |  | 0 | 29 | 1 | 30 |
|  | OtsLNFH_seg_sp16 |  |  | 0 |  |  | 0 |  |  | 0 | 1 |  | 1 |  |  | 0 | 1 | 0 | 1 |
|  | OtsLNFH_seg_sp17 |  |  | 0 |  |  | 0 |  |  | 0 | 69 | 6 | 75 | 1 |  | 1 | 70 | 6 | 76 |
|  | OtsLNFH_seg_sp18 |  |  | 0 |  |  | 0 |  |  | 0 |  |  | 0 | 37 | 3 | 40 | 37 | 3 | 40 |
|  | OtsMETH_seg_sp14 |  |  | 0 |  | 4 | 4 |  | 1 | 1 |  |  | 0 |  |  | 0 | 0 | 5 | 5 |
|  | OtsMETH_seg_sp15 |  |  | 0 |  |  | 0 |  | 5 | 5 |  |  | 0 |  |  | 0 | 0 | 5 | 5 |
|  | OtsMETH_seg_sp16 |  |  | 0 |  |  | 0 |  |  | 0 |  | 1 | 1 |  |  | 0 | 0 | 1 | 1 |
|  | OtsMETH_seg_sp17 |  |  | 0 |  |  | 0 |  |  | 0 |  | 6 | 6 |  |  | 0 | 0 | 6 | 6 |
|  | OtsMETH_seg_sp18 |  |  | 0 |  |  | 0 |  |  | 0 |  |  | 0 | 1 | 6 | 7 | 1 | 6 | 7 |
|  | OtsWINT_seg_sp13 | 8 | 1 | 9 | 1 |  | 1 |  |  | 0 |  |  | 0 |  |  | 0 | 9 | 1 | 10 |
|  | OtsWINT_seg_sp14 | 1 | 2 | 3 | 20 | 9 | 29 |  |  | 0 |  |  | 0 |  |  | 0 | 21 | 11 | 32 |
|  | OtsWINT_seg_sp15 |  |  | 0 |  |  | 0 | 30 | 16 | 46 |  |  | 0 |  |  | 0 | 30 | 16 | 46 |
|  | OtsWINT_seg_sp17 |  |  | 0 |  |  | 0 |  |  | 0 | 5 | 18 | 23 |  |  | 0 | 5 | 18 | 23 |
|  | OtsWINT_seg_sp18 |  |  | 0 |  |  | 0 |  |  | 0 | 1 |  | 1 | 14 | 1 | 15 | 15 | 1 | 16 |
| 11_TUCANO | OtsLYON_seg_sp13 |  | 4 | 4 |  |  | 0 |  |  | 0 |  |  | 0 |  |  | 0 | 0 | 4 | 4 |
|  | OtsLYON_seg_sp14 |  |  | 0 |  | 7 | 7 |  |  | 0 |  |  | 0 |  |  | 0 | 0 | 7 | 7 |
|  | OtsLYON_seg_sp15 |  |  | 0 |  |  | 0 |  | 2 | 2 |  |  | 0 |  |  | 0 | 0 | 2 | 2 |
| 12_HELLSC | OtsCLWH_seg_sp13 | 29 | 3 | 32 |  |  | 0 |  |  | 0 |  |  | 0 |  |  | 0 | 29 | 3 | 32 |
|  | OtsCLWH_seg_sp14 | 10 | 3 | 13 | 88 | 14 | 102 | 6 |  | 6 |  |  | 0 |  |  | 0 | 104 | 17 | 121 |
|  | OtsCLWH_seg_sp15 |  |  | 0 |  |  | 0 | 51 | 4 | 55 |  |  | 0 |  |  | 0 | 51 | 4 | 55 |
|  | OtsCLWH_seg_sp16 |  |  | 0 |  |  | 0 |  |  | 0 | 2 |  | 2 |  |  | 0 | 2 | 0 | 2 |
|  | OtsCLWH_seg_sp17 |  |  | 0 |  |  | 0 |  |  | 0 | 40 | 7 | 47 |  |  | 0 | 40 | 7 | 47 |
|  | OtsCLWH_seg_sp18 |  |  | 0 |  |  | 0 |  |  | 0 |  |  | 0 | 35 | 3 | 38 | 35 | 3 | 38 |
|  | OtsCLWH_seg_sp19 |  |  | 0 |  |  | 0 |  |  | 0 |  |  | 0 | 1 |  | 1 | 1 | 0 | 1 |
|  | OtsDWOR_seg_sp12 | 4 |  | 4 |  |  | 0 |  |  | 0 |  |  | 0 |  |  | 0 | 4 | 0 | 4 |
|  | OtsDWOR_seg_sp13 | 20 | 1 | 21 |  |  | 0 |  |  | 0 |  |  | 0 |  |  | 0 | 20 | 1 | 21 |
|  | OtsDWOR_seg_sp14 | 2 |  | 2 | 44 | 3 | 47 | 3 |  | 3 |  |  | 0 |  |  | 0 | 49 | 3 | 52 |
|  | OtsDWOR_seg_sp15 |  |  | 0 |  |  | 0 | 48 | 3 | 51 |  |  | 0 |  |  | 0 | 48 | 3 | 51 |
|  | OtsDWOR_seg_sp17 |  |  | 0 |  |  | 0 |  |  | 0 | 81 | 11 | 92 |  | 1 | 1 | 81 | 12 | 93 |
|  | OtsDWOR_seg_sp18 |  |  | 0 |  |  | 0 |  |  | 0 |  |  | 0 | 66 | 8 | 74 | 66 | 8 | 74 |
|  | OtsIMNW_seg_ss13 | 7 |  | 7 |  |  | 0 |  |  | 0 |  |  | 0 |  |  | 0 | 7 | 0 | 7 |
|  | OtsIMNW_seg_ss14 |  |  | 0 | 8 |  | 8 | 2 |  | 2 |  |  | 0 |  |  | 0 | 10 | 0 | 10 |
|  | OtsIMNW_seg_ss15 |  |  | 0 |  |  | 0 | 8 | 1 | 9 |  |  | 0 |  |  | 0 | 8 | 1 | 9 |
|  | OtsIMNW_seg_ss16 |  |  | 0 |  |  | 0 | 1 |  | 1 | 2 |  | 2 |  |  | 0 | 3 | 0 | 3 |
|  | OtsIMNW_seg_ss17 |  |  | 0 |  |  | 0 |  |  | 0 | 17 | 1 | 18 | 2 |  | 2 | 19 | 1 | 20 |
|  | OtsIMNW_seg_ss18 |  |  | 0 |  |  | 0 |  |  | 0 |  |  | 0 | 4 |  | 4 | 4 | 0 | 4 |
|  | OtsIMNW_seg_ss19 |  |  | 0 |  |  | 0 |  |  | 0 |  |  | 0 | 1 |  | 1 | 1 | 0 | 1 |
|  | OtsKOOS_seg_sp17 |  |  | 0 |  |  | 0 |  |  | 0 | 23 | 2 | 25 |  |  | 0 | 23 | 2 | 25 |
|  | OtsKOOS_seg_sp18 |  |  | 0 |  |  | 0 |  |  | 0 |  |  | 0 | 47 | 7 | 54 | 47 | 7 | 54 |
|  | OtsKOOS_seg_sp19 |  |  | 0 |  |  | 0 |  |  | 0 |  |  | 0 | 1 |  | 1 | 1 | 0 | 1 |
|  | OtsLOOK_seg_sp13 | 11 | 2 | 13 |  |  | 0 |  |  | 0 |  |  | 0 |  |  | 0 | 11 | 2 | 13 |
|  | OtsLOOK_seg_sp14 | 1 |  | 1 | 22 | 7 | 29 | 2 |  | 2 |  |  | 0 |  |  | 0 | 25 | 7 | 32 |
|  | OtsLOOK_seg_sp15 |  |  | 0 |  |  | 0 | 23 | 4 | 27 |  |  | 0 |  |  | 0 | 23 | 4 | 27 |
|  | OtsLOOK_seg_sp17 |  |  | 0 |  |  | 0 |  |  | 0 | 32 | 4 | 36 |  |  | 0 | 32 | 4 | 36 |
|  | OtsLOOK_seg_sp18 |  |  | 0 |  |  | 0 |  |  | 0 |  |  | 0 | 20 | 3 | 23 | 20 | 3 | 23 |
|  | OtsNPFH_seg_sp13 |  | 2 | 2 |  |  | 0 |  |  | 0 |  |  | 0 |  |  | 0 | 0 | 2 | 2 |
|  | OtsNPFH_seg_sp14 |  |  | 0 |  | 3 | 3 |  |  | 0 |  |  | 0 |  |  | 0 | 0 | 3 | 3 |
|  | OtsNPFH_seg_sp17 |  |  | 0 |  |  | 0 |  |  | 0 |  | 3 | 3 |  |  | 0 | 0 | 3 | 3 |
|  | OtsNPFH_seg_sp18 |  |  | 0 |  |  | 0 |  |  | 0 |  |  | 0 |  | 2 | 2 | 0 | 2 | 2 |
|  | OtsPOWP_seg_sp13 | 8 | 1 | 9 | 1 |  | 1 |  |  | 0 |  |  | 0 |  |  | 0 | 9 | 1 | 10 |
|  | OtsPOWP_seg_sp14 | 2 |  | 2 | 43 | 7 | 50 | 2 |  | 2 |  |  | 0 |  |  | 0 | 47 | 7 | 54 |
|  | OtsPOWP_seg_sp15 |  |  | 0 |  |  | 0 | 29 | 10 | 39 |  |  | 0 |  |  | 0 | 29 | 10 | 39 |
|  | OtsPOWP_seg_sp17 |  |  | 0 |  |  | 0 |  |  | 0 | 1 | 2 | 3 |  |  | 0 | 1 | 2 | 3 |
|  | OtsPOWP_seg_sp18 |  |  | 0 |  |  | 0 |  |  | 0 | 1 |  | 1 | 2 | 6 | 8 | 3 | 6 | 9 |
|  | OtsRAPH_seg_sp12 | 6 |  | 6 |  |  | 0 |  |  | 0 |  |  | 0 |  |  | 0 | 6 | 0 | 6 |
|  | OtsRAPH_seg_sp13 | 149 | 4 | 153 | 3 |  | 3 |  |  | 0 |  |  | 0 |  |  | 0 | 152 | 4 | 156 |
|  | OtsRAPH_seg_sp14 | 3 |  | 3 | 150 | 3 | 153 | 9 |  | 9 |  |  | 0 |  |  | 0 | 162 | 3 | 165 |
|  | OtsRAPH_seg_sp15 |  |  | 0 |  |  | 0 | 146 | 4 | 150 |  |  | 0 |  |  | 0 | 146 | 4 | 150 |
|  | OtsRAPH_seg_sp16 |  |  | 0 |  |  | 0 |  |  | 0 | 1 |  | 1 |  |  | 0 | 1 | 0 | 1 |
|  | OtsRAPH_seg_sp17 |  |  | 0 |  |  | 0 |  |  | 0 | 184 | 6 | 190 | 3 |  | 3 | 187 | 6 | 193 |
|  | OtsRAPH_seg_sp18 |  |  | 0 |  |  | 0 |  |  | 0 |  |  | 0 | 82 | 5 | 87 | 82 | 5 | 87 |
| 13_SFSALM | OtsJHNW_int_ss14 |  |  | 0 |  | 3 | 3 |  |  | 0 |  |  | 0 |  |  | 0 | 0 | 3 | 3 |
|  | OtsJHNW_int_ss17 |  |  | 0 |  |  | 0 |  |  | 0 |  | 2 | 2 |  |  | 0 | 0 | 2 | 2 |
|  | OtsJHNW_int_ss18 |  |  | 0 |  |  | 0 |  |  | 0 |  |  | 0 |  | 2 | 2 | 0 | 2 | 2 |
|  | OtsMCCA_seg_ss12 |  | 1 | 1 |  |  | 0 |  |  | 0 |  |  | 0 |  |  | 0 | 0 | 1 | 1 |
|  | OtsMCCA_seg_ss13 | 28 | 13 | 41 | 1 |  | 1 |  |  | 0 |  |  | 0 |  |  | 0 | 29 | 13 | 42 |
|  | OtsMCCA_seg_ss14 |  |  | 0 | 31 | 8 | 39 |  |  | 0 |  |  | 0 |  |  | 0 | 31 | 8 | 39 |
|  | OtsMCCA_seg_ss15 |  |  | 0 |  |  | 0 | 41 | 14 | 55 |  |  | 0 |  |  | 0 | 41 | 14 | 55 |
|  | OtsMCCA_seg_ss17 |  |  | 0 |  |  | 0 |  |  | 0 | 56 | 18 | 74 |  |  | 0 | 56 | 18 | 74 |
|  | OtsMCCA_seg_ss18 |  |  | 0 |  |  | 0 |  |  | 0 |  |  | 0 | 28 | 7 | 35 | 28 | 7 | 35 |
|  | OtsMCCA_seg_ss19 |  |  | 0 |  |  | 0 |  |  | 0 |  |  | 0 |  | 1 | 1 | 0 | 1 | 1 |
| 16_UPSALM | OtsPAHH_seg_ss13 | 6 | 1 | 7 |  |  | 0 |  |  | 0 |  |  | 0 |  |  | 0 | 6 | 1 | 7 |
|  | OtsPAHH_seg_ss14 |  |  | 0 | 24 |  | 24 | 1 |  | 1 |  |  | 0 |  |  | 0 | 25 | 0 | 25 |
|  | OtsPAHH_seg_ss15 |  |  | 0 |  |  | 0 | 12 |  | 12 |  |  | 0 |  |  | 0 | 12 | 0 | 12 |
|  | OtsPAHH_seg_ss17 |  |  | 0 |  |  | 0 |  |  | 0 | 1 |  | 1 |  |  | 0 | 1 | 0 | 1 |
|  | OtsPAHH_seg_ss18 |  |  | 0 |  |  | 0 |  |  | 0 |  |  | 0 | 21 | 4 | 25 | 21 | 4 | 25 |
|  | OtsSAWT_seg_ss12 | 1 |  | 1 |  |  | 0 |  |  | 0 |  |  | 0 |  |  | 0 | 1 | 0 | 1 |
|  | OtsSAWT_seg_ss13 | 22 |  | 22 | 1 |  | 1 |  |  | 0 |  |  | 0 |  |  | 0 | 23 | 0 | 23 |
|  | OtsSAWT_seg_ss14 | 1 |  | 1 | 64 | 6 | 70 | 3 |  | 3 |  |  | 0 |  |  | 0 | 68 | 6 | 74 |
|  | OtsSAWT_seg_ss15 |  |  | 0 |  |  | 0 | 19 |  | 19 |  |  | 0 |  |  | 0 | 19 | 0 | 19 |
|  | OtsSAWT_seg_ss17 |  |  | 0 |  |  | 0 |  |  | 0 | 19 | 7 | 26 |  |  | 0 | 19 | 7 | 26 |
|  | OtsSAWT_seg_ss18 |  |  | 0 |  |  | 0 |  |  | 0 |  | 1 | 1 | 24 | 3 | 27 | 24 | 4 | 28 |
| 18_UCOLSF | OtsCHJO_int_su13 | 3 |  | 3 | 1 |  | 1 |  |  | 0 |  |  | 0 |  |  | 0 | 4 | 0 | 4 |
|  | OtsCHJO_int_su14 |  |  | 0 | 7 | 2 | 9 | 19 | 1 | 20 |  |  | 0 |  |  | 0 | 26 | 3 | 29 |
|  | OtsCHJO_int_su15 |  |  | 0 |  |  | 0 | 7 | 1 | 8 | 1 |  | 1 |  |  | 0 | 8 | 1 | 9 |
|  | OtsCHJO_int_su16 |  |  | 0 |  |  | 0 |  |  | 0 | 11 |  | 11 |  |  | 0 | 11 | 0 | 11 |
|  | OtsCHJO_int_su17 |  |  | 0 |  |  | 0 |  |  | 0 | 2 |  | 2 | 10 | 3 | 13 | 12 | 3 | 15 |
|  | OtsCHJO_seg_su13 | 1 |  | 1 | 3 |  | 3 | 1 |  | 1 |  |  | 0 |  |  | 0 | 5 | 0 | 5 |
|  | OtsCHJO_seg_su14 |  |  | 0 | 7 |  | 7 | 9 |  | 9 |  |  | 0 |  |  | 0 | 16 | 0 | 16 |
|  | OtsCHJO_seg_su15 |  |  | 0 |  |  | 0 | 7 | 1 | 8 |  |  | 0 |  |  | 0 | 7 | 1 | 8 |
|  | OtsCHJO_seg_su16 |  |  | 0 |  |  | 0 |  |  | 0 | 24 | 2 | 26 | 1 |  | 1 | 25 | 2 | 27 |
|  | OtsCHJO_seg_su17 |  |  | 0 |  |  | 0 |  |  | 0 | 11 | 3 | 14 | 10 | 2 | 12 | 21 | 5 | 26 |
|  | OtsEAST_seg_su12 | 19 | 1 | 20 | 3 |  | 3 |  |  | 0 |  |  | 0 |  |  | 0 | 22 | 1 | 23 |
|  | OtsEAST_seg_su13 | 3 |  | 3 | 6 | 1 | 7 | 1 |  | 1 |  |  | 0 |  |  | 0 | 10 | 1 | 11 |
|  | OtsEAST_seg_su14 |  |  | 0 | 18 | 2 | 20 | 50 | 1 | 51 |  |  | 0 |  |  | 0 | 68 | 3 | 71 |
|  | OtsEAST_seg_su15 |  |  | 0 |  |  | 0 | 12 | 1 | 13 |  |  | 0 |  |  | 0 | 12 | 1 | 13 |
|  | OtsEAST_seg_su16 |  |  | 0 |  |  | 0 |  |  | 0 | 51 | 3 | 54 |  |  | 0 | 51 | 3 | 54 |
|  | OtsEAST_seg_su17 |  |  | 0 |  |  | 0 |  |  | 0 | 20 | 1 | 21 | 24 |  | 24 | 44 | 1 | 45 |
|  | OtsEAST_seg_su18 |  |  | 0 |  |  | 0 |  |  | 0 |  |  | 0 | 16 | 2 | 18 | 16 | 2 | 18 |
|  | OtsENFH_seg_su14 |  |  | 0 | 8 | 2 | 10 | 9 |  | 9 |  |  | 0 |  |  | 0 | 17 | 2 | 19 |
|  | OtsENFH_seg_su15 |  |  | 0 |  |  | 0 | 12 |  | 12 |  |  | 0 |  |  | 0 | 12 | 0 | 12 |
|  | OtsENFH_seg_su16 |  |  | 0 |  |  | 0 |  |  | 0 | 14 | 1 | 15 |  |  | 0 | 14 | 1 | 15 |
|  | OtsENFH_seg_su17 |  |  | 0 |  |  | 0 |  |  | 0 | 14 |  | 14 | 19 |  | 19 | 33 | 0 | 33 |
|  | OtsENFH_seg_su18 |  |  | 0 |  |  | 0 |  |  | 0 |  |  | 0 | 11 |  | 11 | 11 | 0 | 11 |
|  | OtsWELL_seg_su12 | 18 | 2 | 20 |  | 1 | 1 |  |  | 0 |  |  | 0 |  |  | 0 | 18 | 3 | 21 |
|  | OtsWELL_seg_su13 | 19 | 2 | 21 | 22 | 1 | 23 |  |  | 0 |  |  | 0 |  |  | 0 | 41 | 3 | 44 |
|  | OtsWELL_seg_su14 |  |  | 0 | 15 | 1 | 16 | 42 | 1 | 43 |  |  | 0 |  |  | 0 | 57 | 2 | 59 |
|  | OtsWELL_seg_su15 |  |  | 0 | 1 |  | 1 | 15 | 3 | 18 |  |  | 0 |  |  | 0 | 16 | 3 | 19 |
|  | OtsWELL_seg_su16 |  |  | 0 |  |  | 0 |  |  | 0 | 32 | 7 | 39 | 1 |  | 1 | 33 | 7 | 40 |
|  | OtsWELL_seg_su17 |  |  | 0 |  |  | 0 |  |  | 0 | 14 | 1 | 15 | 8 | 3 | 11 | 22 | 4 | 26 |
|  | OtsWELL_seg_su18 |  |  | 0 |  |  | 0 |  |  | 0 |  | 1 | 1 | 5 | 4 | 9 | 5 | 5 | 10 |
|  | OtsWELL_seg_su19 |  |  | 0 |  |  | 0 |  |  | 0 |  |  | 0 | 2 |  | 2 | 2 | 0 | 2 |
| 20_BONPOOLSP | OtsCARS_seg_sp13 | 45 | 3 | 48 | 1 |  | 1 |  |  | 0 |  |  | 0 |  |  | 0 | 46 | 3 | 49 |
|  | OtsCARS_seg_sp14 | 2 |  | 2 | 44 | 4 | 48 | 4 |  | 4 |  |  | 0 |  |  | 0 | 50 | 4 | 54 |
|  | OtsCARS_seg_sp15 |  |  | 0 |  |  | 0 | 56 | 3 | 59 |  |  | 0 |  |  | 0 | 56 | 3 | 59 |
|  | OtsCARS_seg_sp16 |  |  | 0 |  |  | 0 |  |  | 0 | 1 |  | 1 |  |  | 0 | 1 | 0 | 1 |
|  | OtsCARS_seg_sp17 |  |  | 0 |  |  | 0 |  |  | 0 | 73 | 1 | 74 | 1 |  | 1 | 74 | 1 | 75 |
|  | OtsCARS_seg_sp18 |  |  | 0 |  |  | 0 |  |  | 0 |  |  | 0 | 60 | 6 | 66 | 60 | 6 | 66 |
|  | OtsLWSN_seg_sp13 | 58 | 4 | 62 | 3 |  | 3 |  |  | 0 |  |  | 0 |  |  | 0 | 61 | 4 | 65 |
|  | OtsLWSN_seg_sp14 | 3 |  | 3 | 93 | 5 | 98 | 8 |  | 8 |  |  | 0 |  |  | 0 | 104 | 5 | 109 |
|  | OtsLWSN_seg_sp15 |  |  | 0 |  |  | 0 | 60 | 1 | 61 |  |  | 0 |  |  | 0 | 60 | 1 | 61 |
|  | OtsLWSN_seg_sp16 |  |  | 0 |  |  | 0 |  |  | 0 | 1 | 1 | 2 |  |  | 0 | 1 | 1 | 2 |
|  | OtsLWSN_seg_sp17 |  |  | 0 |  |  | 0 |  |  | 0 | 39 | 2 | 41 |  |  | 0 | 39 | 2 | 41 |
|  | OtsLWSN_seg_sp18 |  |  | 0 |  |  | 0 |  |  | 0 |  |  | 0 | 50 | 3 | 53 | 50 | 3 | 53 |
| 21_UMATILLASP | OtsUMAT_seg_sp12 | 1 |  | 1 |  |  | 0 |  |  | 0 |  |  | 0 |  |  | 0 | 1 | 0 | 1 |
|  | OtsUMAT_seg_sp13 | 53 | 2 | 55 | 2 | 1 | 3 |  |  | 0 |  |  | 0 |  |  | 0 | 55 | 3 | 58 |
|  | OtsUMAT_seg_sp14 | 10 | 1 | 11 | 76 | 11 | 87 | 1 |  | 1 |  |  | 0 |  |  | 0 | 87 | 12 | 99 |
|  | OtsUMAT_seg_sp15 |  |  | 0 |  |  | 0 | 4 | 12 | 16 |  |  | 0 |  |  | 0 | 4 | 12 | 16 |
|  | OtsUMAT_seg_sp17 |  |  | 0 |  |  | 0 |  |  | 0 | 27 | 2 | 29 |  |  | 0 | 27 | 2 | 29 |
|  | OtsUMAT_seg_sp18 |  |  | 0 |  |  | 0 |  |  | 0 |  |  | 0 | 21 | 4 | 25 | 21 | 4 | 25 |
|  | OtsUMAT_seg_sp19 |  |  | 0 |  |  | 0 |  |  | 0 |  |  | 0 |  | 1 | 1 | 0 | 1 | 1 |
|  | Total | 668 | 58 | 726 | 927 | 115 | 1042 | 860 | 111 | 971 | 993 | 143 | 1136 | 712 | 115 | 827 | 4160 | 542 | 4702 |

Table S8. Broodstock abundances at Bonneville Dam (2017 – 2022).

| Gen Stock |  | 2017 | | | 2018 | | | 2019 | | | 2021 | | | 2022 | | | Grand Total | | |
| --- | --- | --- | --- | --- | --- | --- | --- | --- | --- | --- | --- | --- | --- | --- | --- | --- | --- | --- | --- |
| Exp | Hatchery broodstock | H | HNC | Total | H | HNC | Total | H | HNC | Total | H | HNC | Total | H | HNC | Total | H | HNC | Total |
| 02_WCASSP | OtsPARK_seg_sp13 | 235 |  | 235 |  |  | 0 |  |  | 0 |  |  | 0 |  |  | 0 | 235 | 0 | 235 |
|  | OtsPARK_seg_sp14 | 533 |  | 533 | 2,126 | 55 | 2,181 | 70 |  | 70 |  |  | 0 |  |  | 0 | 2,729 | 55 | 2,784 |
|  | OtsPARK_seg_sp15 |  |  | 0 |  |  | 0 | 717 | 43 | 760 |  |  | 0 |  |  | 0 | 717 | 43 | 760 |
|  | OtsPARK_seg_sp16 |  |  | 0 |  |  | 0 |  |  | 0 | 52 |  | 52 |  |  | 0 | 52 | 0 | 52 |
|  | OtsPARK_seg_sp17 |  |  | 0 |  |  | 0 |  |  | 0 | 390 |  | 390 |  |  | 0 | 390 | 0 | 390 |
|  | OtsPARK_seg_sp18 |  |  | 0 |  |  | 0 |  |  | 0 | 88 | 0 | 88 | 2,615 | 235 | 2,850 | 2,703 | 235 | 2,937 |
| 06_KLICKR | OtsKLIC_seg_sp12 | 452 |  | 452 |  |  | 0 |  |  | 0 |  |  | 0 |  |  | 0 | 452 | 0 | 452 |
|  | OtsKLIC_seg_sp13 | 2,341 |  | 2,341 | 157 |  | 157 |  |  | 0 |  |  | 0 |  |  | 0 | 2,497 | 0 | 2,497 |
|  | OtsKLIC_seg_sp14 | 262 |  | 262 | 664 |  | 664 | 66 |  | 66 |  |  | 0 |  |  | 0 | 992 | 0 | 992 |
|  | OtsKLIC_seg_sp16 |  |  | 0 |  |  | 0 |  |  | 0 | 238 |  | 238 |  |  | 0 | 238 | 0 | 238 |
|  | OtsKLIC_seg_sp17 |  |  | 0 |  |  | 0 |  |  | 0 | 896 |  | 896 |  |  | 0 | 896 | 0 | 896 |
|  | OtsKLIC_seg_sp18 |  |  | 0 |  |  | 0 |  |  | 0 | 58 |  | 58 | 1,772 | 123 | 1,895 | 1,830 | 123 | 1,953 |
| 07_DESCSP | OtsRBFH_seg_sp13 | 3,101 | 142 | 3,242 |  |  | 0 |  |  | 0 |  |  | 0 |  |  | 0 | 3,101 | 142 | 3,242 |
|  | OtsRBFH_seg_sp14 | 953 |  | 953 | 1,223 | 52 | 1,276 |  |  | 0 |  |  | 0 |  |  | 0 | 2,177 | 52 | 2,229 |
|  | OtsRBFH_seg_sp15 |  |  | 0 |  |  | 0 | 450 | 109 | 559 |  |  | 0 |  |  | 0 | 450 | 109 | 559 |
|  | OtsRBFH_seg_sp17 |  |  | 0 |  |  | 0 |  |  | 0 | 502 | 65 | 568 |  |  | 0 | 502 | 65 | 568 |
|  | OtsRBFH_seg_sp18 |  |  | 0 |  |  | 0 |  |  | 0 | 166 | 57 | 223 | 3,409 |  | 3,409 | 3,576 | 57 | 3,633 |
|  | OtsWSNF_seg_sp13 | 2,715 | 193 | 2,908 |  |  | 0 |  |  | 0 |  |  | 0 |  |  | 0 | 2,715 | 193 | 2,908 |
|  | OtsWSNF_seg_sp14 |  |  | 0 |  | 208 | 208 |  |  | 0 |  |  | 0 |  |  | 0 | 0 | 208 | 208 |
|  | OtsWSNF_seg_sp15 |  |  | 0 |  |  | 0 | 754 |  | 754 |  |  | 0 |  |  | 0 | 754 | 0 | 754 |
|  | OtsWSNF_seg_sp17 |  |  | 0 |  |  | 0 |  |  | 0 | 3,668 | 78 | 3,746 | 214 |  | 214 | 3,882 | 78 | 3,960 |
|  | OtsWSNF_seg_sp18 |  |  | 0 |  |  | 0 |  |  | 0 |  | 0 | 0 | 2,042 |  | 2,042 | 2,042 | 0 | 2,042 |
| 09_YAKIMA | OtsYRRD_int_sp13 | 739 |  | 739 |  |  | 0 |  |  | 0 |  |  | 0 |  |  | 0 | 739 | 0 | 739 |
|  | OtsYRRD_int_sp14 | 267 |  | 267 | 2,544 |  | 2,544 |  |  | 0 |  |  | 0 |  |  | 0 | 2,811 | 0 | 2,811 |
|  | OtsYRRD_int_sp15 |  |  | 0 |  |  | 0 | 644 | 42 | 686 |  |  | 0 |  |  | 0 | 644 | 42 | 686 |
|  | OtsYRRD_int_sp17 |  |  | 0 |  |  | 0 |  |  | 0 | 936 | 58 | 994 |  |  | 0 | 936 | 58 | 994 |
|  | OtsYRRD_int_sp18 |  |  | 0 |  |  | 0 |  |  | 0 | 0 |  | 0 | 1,689 | 121 | 1,810 | 1,689 | 121 | 1,810 |
|  | OtsYRRD_seg_sp13 | 917 |  | 917 |  |  | 0 |  |  | 0 |  |  | 0 |  |  | 0 | 917 | 0 | 917 |
|  | OtsYRRD_seg_sp14 |  |  | 0 | 605 |  | 605 |  |  | 0 |  |  | 0 |  |  | 0 | 605 | 0 | 605 |
|  | OtsYRRD_seg_sp15 |  |  | 0 |  |  | 0 | 131 |  | 131 |  |  | 0 |  |  | 0 | 131 | 0 | 131 |
|  | OtsYRRD_seg_sp17 |  |  | 0 |  |  | 0 |  |  | 0 | 193 | 45 | 237 |  |  | 0 | 193 | 45 | 237 |
|  | OtsYRRD_seg_sp18 |  |  | 0 |  |  | 0 |  |  | 0 | 0 |  | 0 | 358 |  | 358 | 358 | 0 | 358 |
| 10_UCOLSP | OtsCHJO_seg_sp14 |  |  | 0 | 590 | 126 | 716 |  |  | 0 |  |  | 0 |  |  | 0 | 590 | 126 | 716 |
|  | OtsCHJO_seg_sp15 |  |  | 0 |  |  | 0 | 1,608 | 85 | 1,693 |  |  | 0 |  |  | 0 | 1,608 | 85 | 1,693 |
|  | OtsCHJO_seg_sp17 |  |  | 0 |  |  | 0 |  |  | 0 | 548 |  | 548 |  |  | 0 | 548 | 0 | 548 |
|  | OtsEAST_seg_sp13 | 741 | 142 | 883 |  |  | 0 |  |  | 0 |  |  | 0 |  |  | 0 | 741 | 142 | 883 |
|  | OtsEAST_seg_sp14 |  | 99 | 99 | 526 | 175 | 701 |  | 85 | 85 |  |  | 0 |  |  | 0 | 526 | 359 | 885 |
|  | OtsEAST_seg_sp15 |  |  | 0 |  | 67 | 67 | 243 | 708 | 952 |  |  | 0 |  |  | 0 | 243 | 775 | 1,019 |
|  | OtsEAST_seg_sp16 |  |  | 0 |  |  | 0 |  | 64 | 64 |  |  | 0 |  |  | 0 | 0 | 64 | 64 |
|  | OtsEAST_seg_sp17 |  |  | 0 |  |  | 0 |  |  | 0 | 657 | 981 | 1,638 |  |  | 0 | 657 | 981 | 1,638 |
|  | OtsEAST_seg_sp18 |  |  | 0 |  |  | 0 |  |  | 0 |  |  | 0 | 1,381 | 2,002 | 3,383 | 1,381 | 2,002 | 3,383 |
|  | OtsEAST_seg_sp19 |  |  | 0 |  |  | 0 |  |  | 0 |  |  | 0 |  | 133 | 133 | 0 | 133 | 133 |
|  | OtsLNFH_seg_sp13 | 1,280 |  | 1,280 |  |  | 0 |  |  | 0 |  |  | 0 |  |  | 0 | 1,280 | 0 | 1,280 |
|  | OtsLNFH_seg_sp14 |  |  | 0 | 1,184 | 52 | 1,236 |  |  | 0 |  |  | 0 |  |  | 0 | 1,184 | 52 | 1,236 |
|  | OtsLNFH_seg_sp15 |  |  | 0 |  |  | 0 | 1,765 | 53 | 1,818 |  |  | 0 |  |  | 0 | 1,765 | 53 | 1,818 |
|  | OtsLNFH_seg_sp16 |  |  | 0 |  |  | 0 |  |  | 0 | 32 |  | 32 |  |  | 0 | 32 | 0 | 32 |
|  | OtsLNFH_seg_sp17 |  |  | 0 |  |  | 0 |  |  | 0 | 4,903 | 484 | 5,387 | 305 |  | 305 | 5,208 | 484 | 5,693 |
|  | OtsLNFH_seg_sp18 |  |  | 0 |  |  | 0 |  |  | 0 |  |  | 0 | 11,665 | 534 | 12,200 | 11,665 | 534 | 12,200 |
|  | OtsMETH_seg_sp14 |  |  | 0 |  | 223 | 223 |  | 46 | 46 |  |  | 0 |  |  | 0 | 0 | 269 | 269 |
|  | OtsMETH_seg_sp15 |  |  | 0 |  |  | 0 |  | 281 | 281 |  |  | 0 |  |  | 0 | 0 | 281 | 281 |
|  | OtsMETH_seg_sp16 |  |  | 0 |  |  | 0 |  |  | 0 |  | 58 | 58 |  |  | 0 | 0 | 58 | 58 |
|  | OtsMETH_seg_sp17 |  |  | 0 |  |  | 0 |  |  | 0 |  | 423 | 423 |  |  | 0 | 0 | 423 | 423 |
|  | OtsMETH_seg_sp18 |  |  | 0 |  |  | 0 |  |  | 0 |  |  | 0 | 219 | 934 | 1,152 | 219 | 934 | 1,152 |
|  | OtsWINT_seg_sp13 | 1,034 | 88 | 1,122 | 132 |  | 132 |  |  | 0 |  |  | 0 |  |  | 0 | 1,166 | 88 | 1,254 |
|  | OtsWINT_seg_sp14 | 197 | 232 | 428 | 1,757 | 546 | 2,303 |  |  | 0 |  |  | 0 |  |  | 0 | 1,953 | 777 | 2,731 |
|  | OtsWINT_seg_sp15 |  |  | 0 |  |  | 0 | 1,515 | 952 | 2,466 |  |  | 0 |  |  | 0 | 1,515 | 952 | 2,466 |
|  | OtsWINT_seg_sp17 |  |  | 0 |  |  | 0 |  |  | 0 | 344 | 1,183 | 1,526 |  |  | 0 | 344 | 1,183 | 1,526 |
|  | OtsWINT_seg_sp18 |  |  | 0 |  |  | 0 |  |  | 0 | 90 |  | 90 | 3,638 | 64 | 3,701 | 3,727 | 64 | 3,791 |
| 11_TUCANO | OtsLYON_seg_sp13 |  | 564 | 564 |  |  | 0 |  |  | 0 |  |  | 0 |  |  | 0 | 0 | 564 | 564 |
|  | OtsLYON_seg_sp14 |  |  | 0 |  | 498 | 498 |  |  | 0 |  |  | 0 |  |  | 0 | 0 | 498 | 498 |
|  | OtsLYON_seg_sp15 |  |  | 0 |  |  | 0 |  | 268 | 268 |  |  | 0 |  |  | 0 | 0 | 268 | 268 |
| 12_HELLSC | OtsCLWH_seg_sp13 | 3,421 | 365 | 3,787 |  |  | 0 |  |  | 0 |  |  | 0 |  |  | 0 | 3,421 | 365 | 3,787 |
|  | OtsCLWH_seg_sp14 | 1,068 | 178 | 1,247 | 7,742 | 862 | 8,603 | 297 |  | 297 |  |  | 0 |  |  | 0 | 9,107 | 1,040 | 10,147 |
|  | OtsCLWH_seg_sp15 |  |  | 0 |  |  | 0 | 2,800 | 198 | 2,997 |  |  | 0 |  |  | 0 | 2,800 | 198 | 2,997 |
|  | OtsCLWH_seg_sp16 |  |  | 0 |  |  | 0 |  |  | 0 | 106 |  | 106 |  |  | 0 | 106 | 0 | 106 |
|  | OtsCLWH_seg_sp17 |  |  | 0 |  |  | 0 |  |  | 0 | 2,447 | 457 | 2,905 |  |  | 0 | 2,447 | 457 | 2,905 |
|  | OtsCLWH_seg_sp18 |  |  | 0 |  |  | 0 |  |  | 0 |  |  | 0 | 6,093 | 365 | 6,458 | 6,093 | 365 | 6,458 |
|  | OtsCLWH_seg_sp19 |  |  | 0 |  |  | 0 |  |  | 0 |  |  | 0 | 137 |  | 137 | 137 | 0 | 137 |
|  | OtsDWOR_seg_sp12 | 437 |  | 437 |  |  | 0 |  |  | 0 |  |  | 0 |  |  | 0 | 437 | 0 | 437 |
|  | OtsDWOR_seg_sp13 | 2,738 | 77 | 2,815 |  |  | 0 |  |  | 0 |  |  | 0 |  |  | 0 | 2,738 | 77 | 2,815 |
|  | OtsDWOR_seg_sp14 | 269 |  | 269 | 3,904 | 168 | 4,072 | 153 |  | 153 |  |  | 0 |  |  | 0 | 4,326 | 168 | 4,494 |
|  | OtsDWOR_seg_sp15 |  |  | 0 |  |  | 0 | 2,565 | 153 | 2,718 |  |  | 0 |  |  | 0 | 2,565 | 153 | 2,718 |
|  | OtsDWOR_seg_sp17 |  |  | 0 |  |  | 0 |  |  | 0 | 5,107 | 705 | 5,812 |  | 51 | 51 | 5,107 | 756 | 5,863 |
|  | OtsDWOR_seg_sp18 |  |  | 0 |  |  | 0 |  |  | 0 |  |  | 0 | 11,826 | 953 | 12,779 | 11,826 | 953 | 12,779 |
|  | OtsIMNW_seg_ss13 | 665 |  | 665 |  |  | 0 |  |  | 0 |  |  | 0 |  |  | 0 | 665 | 0 | 665 |
|  | OtsIMNW_seg_ss14 |  |  | 0 | 592 |  | 592 | 77 |  | 77 |  |  | 0 |  |  | 0 | 669 | 0 | 669 |
|  | OtsIMNW_seg_ss15 |  |  | 0 |  |  | 0 | 370 | 44 | 413 |  |  | 0 |  |  | 0 | 370 | 44 | 413 |
|  | OtsIMNW_seg_ss16 |  |  | 0 |  |  | 0 | 61 |  | 61 | 103 |  | 103 |  |  | 0 | 163 | 0 | 163 |
|  | OtsIMNW_seg_ss17 |  |  | 0 |  |  | 0 |  |  | 0 | 1,138 | 64 | 1,202 | 325 |  | 325 | 1,462 | 64 | 1,526 |
|  | OtsIMNW_seg_ss18 |  |  | 0 |  |  | 0 |  |  | 0 |  |  | 0 | 508 |  | 508 | 508 | 0 | 508 |
|  | OtsIMNW_seg_ss19 |  |  | 0 |  |  | 0 |  |  | 0 |  |  | 0 | 133 |  | 133 | 133 | 0 | 133 |
|  | OtsKOOS_seg_sp17 |  |  | 0 |  |  | 0 |  |  | 0 | 1,500 | 114 | 1,615 |  |  | 0 | 1,500 | 114 | 1,615 |
|  | OtsKOOS_seg_sp18 |  |  | 0 |  |  | 0 |  |  | 0 |  |  | 0 | 9,432 | 836 | 10,268 | 9,432 | 836 | 10,268 |
|  | OtsKOOS_seg_sp19 |  |  | 0 |  |  | 0 |  |  | 0 |  |  | 0 | 98 |  | 98 | 98 | 0 | 98 |
|  | OtsLOOK_seg_sp13 | 1,099 | 217 | 1,316 |  |  | 0 |  |  | 0 |  |  | 0 |  |  | 0 | 1,099 | 217 | 1,316 |
|  | OtsLOOK_seg_sp14 | 98 |  | 98 | 2,077 | 468 | 2,544 | 98 |  | 98 |  |  | 0 |  |  | 0 | 2,273 | 468 | 2,741 |
|  | OtsLOOK_seg_sp15 |  |  | 0 |  |  | 0 | 1,490 | 302 | 1,792 |  |  | 0 |  |  | 0 | 1,490 | 302 | 1,792 |
|  | OtsLOOK_seg_sp17 |  |  | 0 |  |  | 0 |  |  | 0 | 2,081 | 297 | 2,378 |  |  | 0 | 2,081 | 297 | 2,378 |
|  | OtsLOOK_seg_sp18 |  |  | 0 |  |  | 0 |  |  | 0 |  |  | 0 | 3,917 | 479 | 4,397 | 3,917 | 479 | 4,397 |
|  | OtsNPFH_seg_sp13 |  | 281 | 281 |  |  | 0 |  |  | 0 |  |  | 0 |  |  | 0 | 0 | 281 | 281 |
|  | OtsNPFH_seg_sp14 |  |  | 0 |  | 187 | 187 |  |  | 0 |  |  | 0 |  |  | 0 | 0 | 187 | 187 |
|  | OtsNPFH_seg_sp17 |  |  | 0 |  |  | 0 |  |  | 0 |  | 219 | 219 |  |  | 0 | 0 | 219 | 219 |
|  | OtsNPFH_seg_sp18 |  |  | 0 |  |  | 0 |  |  | 0 |  |  | 0 |  | 296 | 296 | 0 | 296 | 296 |
|  | OtsPOWP_seg_sp13 | 824 | 130 | 954 | 104 |  | 104 |  |  | 0 |  |  | 0 |  |  | 0 | 928 | 130 | 1,059 |
|  | OtsPOWP_seg_sp14 | 222 |  | 222 | 3,837 | 423 | 4,260 | 118 |  | 118 |  |  | 0 |  |  | 0 | 4,177 | 423 | 4,600 |
|  | OtsPOWP_seg_sp15 |  |  | 0 |  |  | 0 | 1,563 | 588 | 2,151 |  |  | 0 |  |  | 0 | 1,563 | 588 | 2,151 |
|  | OtsPOWP_seg_sp17 |  |  | 0 |  |  | 0 |  |  | 0 | 73 | 109 | 181 |  |  | 0 | 73 | 109 | 181 |
|  | OtsPOWP_seg_sp18 |  |  | 0 |  |  | 0 |  |  | 0 | 51 | 0 | 51 | 266 | 613 | 879 | 317 | 613 | 930 |
|  | OtsRAPH_seg_sp12 | 610 |  | 610 |  |  | 0 |  |  | 0 |  |  | 0 |  |  | 0 | 610 | 0 | 610 |
|  | OtsRAPH_seg_sp13 | 17,142 | 507 | 17,650 | 232 |  | 232 |  |  | 0 |  |  | 0 |  |  | 0 | 17,375 | 507 | 17,882 |
|  | OtsRAPH_seg_sp14 | 354 |  | 354 | 12,937 | 178 | 13,115 | 490 |  | 490 |  |  | 0 |  |  | 0 | 13,781 | 178 | 13,959 |
|  | OtsRAPH_seg_sp15 |  |  | 0 |  |  | 0 | 8,413 | 311 | 8,724 |  |  | 0 |  |  | 0 | 8,413 | 311 | 8,724 |
|  | OtsRAPH_seg_sp16 |  |  | 0 |  |  | 0 |  |  | 0 | 51 |  | 51 |  |  | 0 | 51 | 0 | 51 |
|  | OtsRAPH_seg_sp17 |  |  | 0 |  |  | 0 |  |  | 0 | 10,947 | 418 | 11,365 | 700 |  | 700 | 11,647 | 418 | 12,065 |
|  | OtsRAPH_seg_sp18 |  |  | 0 |  |  | 0 |  |  | 0 | 0 |  | 0 | 16,301 | 659 | 16,960 | 16,301 | 659 | 16,960 |
| 13_SFSALM | OtsJHNW_int_ss14 |  | 0 | 0 |  | 201 | 201 |  |  | 0 |  |  | 0 |  |  | 0 | 0 | 201 | 201 |
|  | OtsJHNW_int_ss17 |  |  | 0 |  |  | 0 |  |  | 0 |  | 103 | 103 |  |  | 0 | 0 | 103 | 103 |
|  | OtsJHNW_int_ss18 |  |  | 0 |  |  | 0 |  |  | 0 |  |  | 0 |  | 136 | 136 | 0 | 136 | 136 |
|  | OtsMCCA_seg_ss12 |  | 71 | 71 |  |  | 0 |  |  | 0 |  |  | 0 |  |  | 0 | 0 | 71 | 71 |
|  | OtsMCCA_seg_ss13 | 3,011 | 1,190 | 4,201 | 76 |  | 76 |  |  | 0 |  |  | 0 |  |  | 0 | 3,087 | 1,190 | 4,277 |
|  | OtsMCCA_seg_ss14 | 0 | 0 | 0 | 2,346 | 517 | 2,862 |  |  | 0 |  |  | 0 |  |  | 0 | 2,346 | 517 | 2,862 |
|  | OtsMCCA_seg_ss15 |  |  | 0 |  |  | 0 | 2,006 | 799 | 2,805 |  |  | 0 |  |  | 0 | 2,006 | 799 | 2,805 |
|  | OtsMCCA_seg_ss17 |  |  | 0 |  |  | 0 |  |  | 0 | 3,488 | 1,060 | 4,548 |  |  | 0 | 3,488 | 1,060 | 4,548 |
|  | OtsMCCA_seg_ss18 |  |  | 0 |  |  | 0 |  |  | 0 | 0 |  | 0 | 3,222 | 485 | 3,708 | 3,222 | 485 | 3,708 |
|  | OtsMCCA_seg_ss19 |  |  | 0 |  |  | 0 |  |  | 0 |  |  | 0 |  | 122 | 122 | 0 | 122 | 122 |
| 16_UPSALM | OtsPAHH_seg_ss13 | 479 | 50 | 529 |  |  | 0 |  |  | 0 |  |  | 0 |  |  | 0 | 479 | 50 | 529 |
|  | OtsPAHH_seg_ss14 | 0 |  | 0 | 1,790 |  | 1,790 | 51 |  | 51 |  |  | 0 |  |  | 0 | 1,841 | 0 | 1,841 |
|  | OtsPAHH_seg_ss15 |  |  | 0 |  |  | 0 | 600 |  | 600 |  |  | 0 |  |  | 0 | 600 | 0 | 600 |
|  | OtsPAHH_seg_ss17 |  |  | 0 |  |  | 0 |  |  | 0 | 51 |  | 51 |  |  | 0 | 51 | 0 | 51 |
|  | OtsPAHH_seg_ss18 |  |  | 0 |  |  | 0 |  |  | 0 |  |  | 0 | 2,368 | 250 | 2,618 | 2,368 | 250 | 2,618 |
|  | OtsSAWT_seg_ss12 | 184 |  | 184 |  |  | 0 |  |  | 0 |  |  | 0 |  |  | 0 | 184 | 0 | 184 |
|  | OtsSAWT_seg_ss13 | 2,301 |  | 2,301 | 73 |  | 73 |  |  | 0 |  |  | 0 |  |  | 0 | 2,374 | 0 | 2,374 |
|  | OtsSAWT_seg_ss14 | 84 |  | 84 | 5,142 | 329 | 5,471 | 148 |  | 148 |  |  | 0 |  |  | 0 | 5,374 | 329 | 5,704 |
|  | OtsSAWT_seg_ss15 |  |  | 0 |  |  | 0 | 925 |  | 925 |  |  | 0 |  |  | 0 | 925 | 0 | 925 |
|  | OtsSAWT_seg_ss17 |  |  | 0 |  |  | 0 |  |  | 0 | 1,231 | 436 | 1,666 |  |  | 0 | 1,231 | 436 | 1,666 |
|  | OtsSAWT_seg_ss18 |  |  | 0 |  |  | 0 |  |  | 0 |  | 63 | 63 | 3,309 | 288 | 3,597 | 3,309 | 351 | 3,660 |
| 18_UCOLSF | OtsCHJO_int_su13 | 355 |  | 355 | 107 |  | 107 |  |  | 0 |  |  | 0 |  |  | 0 | 461 | 0 | 461 |
|  | OtsCHJO_int_su14 | 0 |  | 0 | 581 | 150 | 731 | 1,263 | 64 | 1,328 |  |  | 0 |  |  | 0 | 1,844 | 215 | 2,059 |
|  | OtsCHJO_int_su15 |  |  | 0 | 0 |  | 0 | 501 | 53 | 554 | 63 |  | 63 |  |  | 0 | 563 | 53 | 617 |
|  | OtsCHJO_int_su16 |  |  | 0 |  |  | 0 | 0 | 0 | 0 | 710 | 0 | 710 |  |  | 0 | 710 | 0 | 710 |
|  | OtsCHJO_int_su17 |  |  | 0 |  |  | 0 |  |  | 0 | 172 | 0 | 172 | 1,565 | 382 | 1,947 | 1,737 | 382 | 2,119 |
|  | OtsCHJO_seg_su13 | 93 | 0 | 93 | 252 | 0 | 252 | 68 |  | 68 |  |  | 0 |  |  | 0 | 412 | 0 | 412 |
|  | OtsCHJO_seg_su14 | 0 |  | 0 | 1,173 |  | 1,173 | 984 |  | 984 |  |  | 0 |  |  | 0 | 2,157 | 0 | 2,157 |
|  | OtsCHJO_seg_su15 |  |  | 0 |  |  | 0 | 470 | 60 | 529 |  |  | 0 |  |  | 0 | 470 | 60 | 529 |
|  | OtsCHJO_seg_su16 |  |  | 0 |  |  | 0 | 0 |  | 0 | 1,427 | 85 | 1,512 | 92 |  | 92 | 1,520 | 85 | 1,604 |
|  | OtsCHJO_seg_su17 |  |  | 0 |  |  | 0 |  |  | 0 | 726 | 156 | 882 | 1,337 | 225 | 1,562 | 2,063 | 382 | 2,444 |
|  | OtsEAST_seg_su12 | 1,569 | 70 | 1,639 | 223 |  | 223 |  |  | 0 |  |  | 0 |  |  | 0 | 1,793 | 70 | 1,862 |
|  | OtsEAST_seg_su13 | 250 |  | 250 | 444 | 67 | 511 | 68 |  | 68 |  |  | 0 |  |  | 0 | 761 | 67 | 828 |
|  | OtsEAST_seg_su14 |  |  | 0 | 1,336 | 134 | 1,470 | 3,145 | 57 | 3,202 |  |  | 0 |  |  | 0 | 4,481 | 191 | 4,672 |
|  | OtsEAST_seg_su15 |  |  | 0 |  |  | 0 | 737 | 48 | 785 | 0 |  | 0 |  |  | 0 | 737 | 48 | 785 |
|  | OtsEAST_seg_su16 |  |  | 0 |  |  | 0 |  |  | 0 | 3,283 | 144 | 3,426 | 0 |  | 0 | 3,283 | 144 | 3,426 |
|  | OtsEAST_seg_su17 |  |  | 0 |  |  | 0 |  |  | 0 | 1,196 | 59 | 1,255 | 2,772 | 0 | 2,772 | 3,968 | 59 | 4,027 |
|  | OtsEAST_seg_su18 |  |  | 0 |  |  | 0 |  |  | 0 | 0 |  | 0 | 1,815 | 205 | 2,020 | 1,815 | 205 | 2,020 |
|  | OtsENFH_seg_su14 |  |  | 0 | 812 | 163 | 975 | 725 |  | 725 |  |  | 0 |  |  | 0 | 1,537 | 163 | 1,700 |
|  | OtsENFH_seg_su15 |  |  | 0 |  |  | 0 | 939 |  | 939 |  |  | 0 |  |  | 0 | 939 | 0 | 939 |
|  | OtsENFH_seg_su16 |  |  | 0 |  |  | 0 |  |  | 0 | 862 | 57 | 919 |  |  | 0 | 862 | 57 | 919 |
|  | OtsENFH_seg_su17 |  |  | 0 |  |  | 0 |  |  | 0 | 971 |  | 971 | 2,447 |  | 2,447 | 3,418 | 0 | 3,418 |
|  | OtsENFH_seg_su18 |  |  | 0 |  |  | 0 |  |  | 0 |  |  | 0 | 1,665 | 0 | 1,665 | 1,665 | 0 | 1,665 |
|  | OtsWELL_seg_su12 | 1,572 | 216 | 1,787 |  | 67 | 67 |  |  | 0 |  |  | 0 |  |  | 0 | 1,572 | 283 | 1,854 |
|  | OtsWELL_seg_su13 | 1,553 | 124 | 1,677 | 1,617 | 67 | 1,684 |  |  | 0 |  |  | 0 |  |  | 0 | 3,170 | 191 | 3,361 |
|  | OtsWELL_seg_su14 | 0 | 0 | 0 | 1,110 | 67 | 1,177 | 2,504 | 48 | 2,552 |  |  | 0 |  |  | 0 | 3,614 | 115 | 3,729 |
|  | OtsWELL_seg_su15 |  |  | 0 | 74 |  | 74 | 850 | 153 | 1,003 |  |  | 0 |  |  | 0 | 925 | 153 | 1,077 |
|  | OtsWELL_seg_su16 |  |  | 0 |  |  | 0 |  |  | 0 | 1,842 | 332 | 2,174 | 91 |  | 91 | 1,934 | 332 | 2,266 |
|  | OtsWELL_seg_su17 |  |  | 0 |  |  | 0 |  |  | 0 | 963 | 49 | 1,012 | 910 | 362 | 1,272 | 1,872 | 411 | 2,283 |
|  | OtsWELL_seg_su18 |  |  | 0 |  |  | 0 |  |  | 0 |  | 53 | 53 | 738 | 454 | 1,193 | 738 | 508 | 1,246 |
|  | OtsWELL_seg_su19 |  |  | 0 |  |  | 0 |  |  | 0 |  |  | 0 | 256 | 0 | 256 | 256 | 0 | 256 |
| 20_BONPOOLSP | OtsCARS_seg_sp13 | 5,942 | 477 | 6,419 | 127 |  | 127 |  |  | 0 |  |  | 0 |  |  | 0 | 6,069 | 477 | 6,546 |
|  | OtsCARS_seg_sp14 | 185 |  | 185 | 4,185 | 267 | 4,452 | 236 |  | 236 |  |  | 0 |  |  | 0 | 4,606 | 267 | 4,873 |
|  | OtsCARS_seg_sp15 |  |  | 0 |  |  | 0 | 3,151 | 139 | 3,290 |  |  | 0 |  |  | 0 | 3,151 | 139 | 3,290 |
|  | OtsCARS_seg_sp16 |  |  | 0 |  |  | 0 |  |  | 0 | 90 |  | 90 |  |  | 0 | 90 | 0 | 90 |
|  | OtsCARS_seg_sp17 |  |  | 0 |  |  | 0 |  |  | 0 | 4,499 | 85 | 4,585 | 173 |  | 173 | 4,672 | 85 | 4,757 |
|  | OtsCARS_seg_sp18 |  |  | 0 |  |  | 0 |  |  | 0 |  |  | 0 | 12,073 | 849 | 12,922 | 12,073 | 849 | 12,922 |
|  | OtsLWSN_seg_sp13 | 6,398 | 411 | 6,809 | 254 |  | 254 |  |  | 0 |  |  | 0 |  |  | 0 | 6,652 | 411 | 7,063 |
|  | OtsLWSN_seg_sp14 | 450 |  | 450 | 7,944 | 297 | 8,240 | 422 |  | 422 |  |  | 0 |  |  | 0 | 8,815 | 297 | 9,112 |
|  | OtsLWSN_seg_sp15 |  |  | 0 |  |  | 0 | 2,973 | 45 | 3,018 |  |  | 0 |  |  | 0 | 2,973 | 45 | 3,018 |
|  | OtsLWSN_seg_sp16 |  |  | 0 |  |  | 0 |  |  | 0 | 73 | 90 | 162 |  |  | 0 | 73 | 90 | 162 |
|  | OtsLWSN_seg_sp17 |  |  | 0 |  |  | 0 |  |  | 0 | 2,354 | 125 | 2,479 |  |  | 0 | 2,354 | 125 | 2,479 |
|  | OtsLWSN_seg_sp18 |  |  | 0 |  |  | 0 |  |  | 0 |  |  | 0 | 10,177 | 389 | 10,566 | 10,177 | 389 | 10,566 |
| 21_UMATILLASP | OtsUMAT_seg_sp12 | 86 |  | 86 |  |  | 0 |  |  | 0 |  |  | 0 |  |  | 0 | 86 | 0 | 86 |
|  | OtsUMAT_seg_sp13 | 6,176 | 272 | 6,448 | 150 | 52 | 202 |  |  | 0 |  |  | 0 |  |  | 0 | 6,326 | 325 | 6,650 |
|  | OtsUMAT_seg_sp14 | 1,434 | 142 | 1,576 | 6,556 | 618 | 7,175 | 66 |  | 66 |  |  | 0 |  |  | 0 | 8,057 | 760 | 8,817 |
|  | OtsUMAT_seg_sp15 |  |  | 0 |  |  | 0 | 215 | 779 | 993 |  |  | 0 |  |  | 0 | 215 | 779 | 993 |
|  | OtsUMAT_seg_sp17 |  |  | 0 |  |  | 0 |  |  | 0 | 1,553 | 168 | 1,721 |  |  | 0 | 1,553 | 168 | 1,721 |
|  | OtsUMAT_seg_sp18 |  |  | 0 |  |  | 0 |  |  | 0 |  |  | 0 | 4,126 | 534 | 4,660 | 4,126 | 534 | 4,660 |
|  | OtsUMAT_seg_sp19 |  |  | 0 |  |  | 0 |  |  | 0 |  |  | 0 |  | 96 | 96 | 0 | 96 | 96 |
|  | Total | 76,833 | 6,238 | 83,071 | 79,304 | 7,285 | 86,589 | 49,505 | 6,573 | 56,078 | 62,919 | 8,882 | 71,801 | 128,176 | 13,177 | 141,353 | 396,736 | 42,155 | 438,891 |
